# Kukhtin–Ramirez-Reaction-Inspired Deprotection of Sulfamidates for the Synthesis of Amino Sugars

**DOI:** 10.3390/molecules28010182

**Published:** 2022-12-25

**Authors:** Ting Li, Bingbing Xu, Dengxian Fu, Qian Wan, Jing Zeng

**Affiliations:** 1 Hubei Key Laboratory of Natural Medicinal Chemistry and Resource Evaluation, School of Pharmacy, Huazhong University of Science and Technology, Wuhan 430030, China; 2 Shandong Provincial Key Laboratory of Carbohydrate Chemistry and Glycobiology, Shandong University, Qingdao 266237, China

**Keywords:** amino alcohols, 3-aminosugars, sulfamidates, deprotection, Kukhtin–Ramirez reaction

## Abstract

Herein, we present a mild strategy for deprotecting cyclic sulfamidates via the Kukhtin–Ramirez reaction to access amino sugars. The method features the removal of the sulfonic group of cyclic sulfamidates, which occurs through an N-H insertion reaction that implicates the Kukhtin–Ramirez adducts, followed by a base-promoted reductive N-S bond cleavage. The mild reaction conditions of the protocol enable the formation of amino alcohols including analogs that bear multiple functional groups.

## 1. Introduction

**Figure 1:**
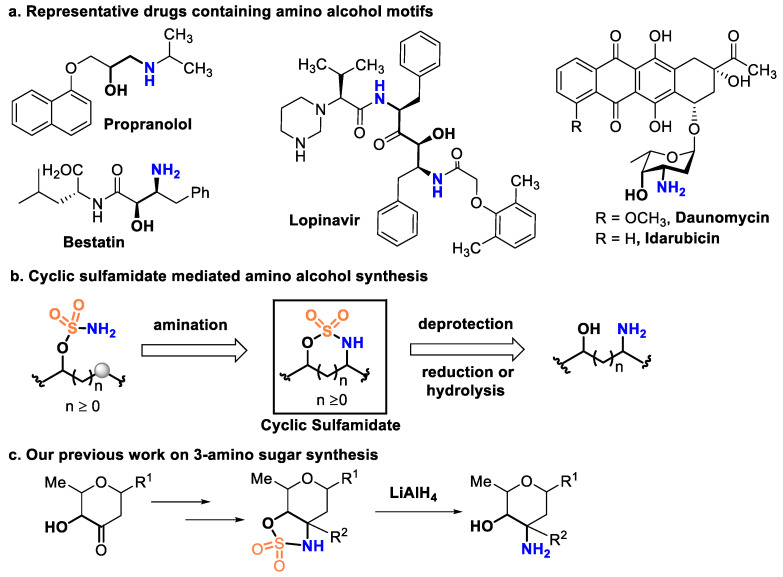
Representative drugs containing amino alcohol motifs and established approaches to synthesizing amino alcohols.

Amino alcohols are important skeletons which are widely distributed in pharmaceuticals and biologically active natural products (Figure 1a) [1,2,3]. Amino alcohols also play important roles in organic synthesis as synthons, ligands, auxiliaries, and chiral catalysts [4,5,6,7,8]. This significance has inspired tremendous efforts to devise elegant synthetic methods for the construction of amino alcohols [9,10,11,12,13,14,15]. Among them, the utility of sulfamate esters [16,17,18,19,20,21] as precursors of amino groups to form cyclic sulfamidate [22,23,24,25] via substitution [26,27], condensation [28,29,30], C-H amination [31,32,33,34], C-H aziridination [35], etc. [36,37,38], has been well established. This has emerged as one of the most prominent methods to produce amino alcohols, due to the ready availability of the materials, the high efficiency of transformations, as well as the well-controlled regioselectivity and stereoselectivity (Figure 1b).

3-Amino deoxy sugars represent a special type of amino alcohols found in many carbohydrate-based antibiotics [39,40]. This strategy has also been incorporated into our study to prepare various 3-amino deoxy sugars (Figure 1c) [41,42,43]. However, the subsequent removal of the SO_2_ group of the cyclic sulfamidate to deliver free amino alcohols presented a notable challenge. Conventional deprotection methods employ strong reducing reagents such as LiAlH_4_, AlH_3,_ and so on [30,44]. Apparently, the functional group tolerance is largely hampered by these conditions, wherein esters, ketones, aldehydes, and so on must be avoided altogether. Another common deprotection method is hydrolysis under acidic or alkaline conditions, but epimerization is always encountered for secondary alcohols [26,45]. To address these limitations and, more importantly, to gain expedite access to diversified 3-amino sugars, we have developed a new deprotection method for the SO_2_ group of sulfamidates under mild conditions.

## 2. Results and Discussion

**Scheme 1:**
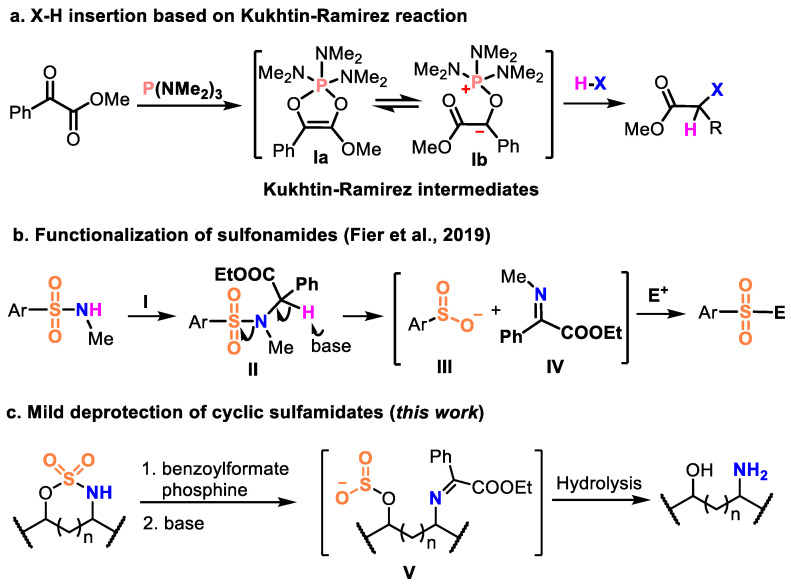
Application of Kukhtin–Ramirez reaction [61].

Our reaction design was inspired by a three-decade-old reaction known as the Kukhtin–Ramirez reaction, which was independently discovered by Kukhtin [46] and Ramirez [47,48]. In this reaction, the redox condensation of a 1,2-dicarbonyl compound with a trivalent phosphorus derivative produces a pentacoordinate dioxaphospholene **Ia**, which exists in equilibrium with a tetracoordinate oxyphosphonium enolate **Ib** (Scheme 1a). Due to their unique properties, these species that are known as the Kukhtin–Ramirez adducts have been well explored in X-H insertion [49,50,51,52], reductive addition [53], cycloaddition [54,55,56], etc. [57,58,59,60]. Very recently, Fier et al. described an ingenious solution to degrade secondary sulfonamides into the corresponding sulfinates by virtue of the Kukhtin–Ramirez adducts [61]. This chemistry involves the addition of sulfonamides onto the Kukhtin–Ramirez adducts to form an N-H insertion intermediate **II**, which undergoes further degradation through a base-promoted reductive cleavage of the N-S bond (Scheme 1b). This unprecedented example of the cleavage of a strong sulfonamide S-N bond led us to envision a similar protocol that might be amenable to cleave the sulfamidate S-N bond to deliver an intermediate (**V**) containing both sulfinate and imine functionalities. The corresponding amino alcohol would be revealed upon hydrolysis (Scheme 1c).

With this idea in mind, our investigation commenced with the deprotection of disaccharide **1a** as the model reaction. The requisite cyclic sulfamidate **1a** used in this study was prepared with the application of the corresponding glycals as starting materials [43]. Initially, disaccharide **1a** was subjected to the Kukhtin–Ramirez intermediate formed from ethyl benzoylformate and tris(dimethylamino)phosphorus (Table 1, entry 1). To our delight, the N-H insertion reaction proceeded smoothly to generate N-sulfonyl phenylglycine ester **3a** in 95% yield, which set the stage for the deprotection reaction. Subsequently, BTMG was added into the system as a base to facilitate the S-N bond cleavage (Table 1, entry 2). As expected, the S-N bond was efficiently cleaved with the removal of the SO_2_ group, but imino ester **4a** was obtained in 79% yield instead of the target free amino alcohols **2a**. This indicated the occurrence of intramolecular esterification prior to the hydrolysis. To avoid this competing reaction, the second step was carried out in an aqueous solution of THF (THF:H_2_O, 1:1). Following this modification, the desired free amino sugar **2a** was obtained in 88% yield. Other than BTMG, DBU and KOH could also yield the target product in 86% and 80% yields, respectively. However, weak bases such as K_2_CO_3_ and Et_3_N lead to dramatic decreases in yield. Interestingly, the basic anion exchange resin Ambersep^®^ 900 (OH) could cleave the S-N bond effectively. This could simplify the product isolation, although the yield of **2a** would be slightly compromised. Considering the product isolation convenience and the price of the reagent (especially in large-scale preparations), DBU was selected as the base. In principle, the 1,2-dicarbonyl entity could be fully recovered, but the ethyl benzoylformate used in this reaction was hydrolyzed under the strong basic conditions. Several other 1,2-dicarbonyl reagents [62,63,64] were subsequently examined to circumvent this process. Unfortunately, none of the examined reagents could promote the preceding N-H insertion reaction (see the SI for the screening of 1,2-dicarbonyl compounds).

**Scheme 2:**
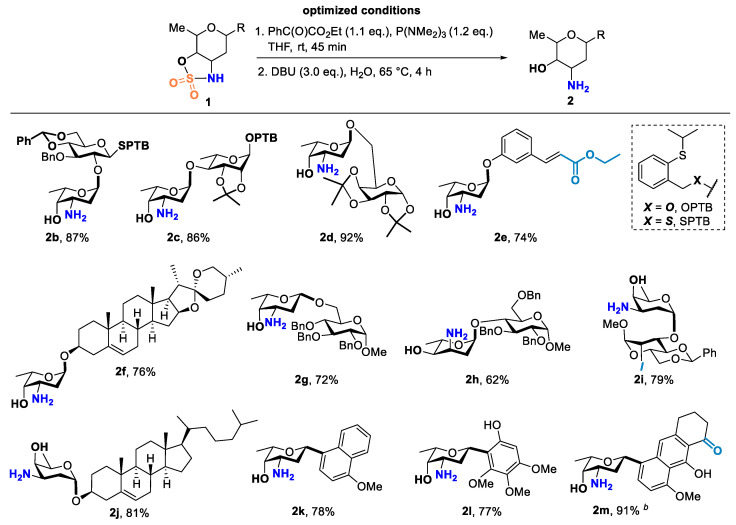
Substrate scope *^a^*. *^a^* Yield of isolated product. *^b^* c = 0.05 M.

With the optimized conditions in place, we then surveyed the scope and limitations of the method, especially in attaining our ultimate goal to prepare 3-aminosugars (Scheme 2). After examining a series of 3-aminosugars, it could be concluded that: (1) The glycosidic bonds (including both O- and C-analogs) were left intact and the optical purities of the α- and β-glycosidic bonds were not eroded at all. (2) All d- and l-sugars of the 3,5-cis or 3,5-trans configuration could undertake the transformation smoothly to produce the cis amino sugars in good yields. (3) Acid-labile groups such as benzylidine acetals (**2b**, **2i**), isopropylidene ketals (**2c**, **2d**), and other ketals (**2f**) as well as alkenes (**2e**) were well tolerated. (4) Functional groups that are generally sensitive to reductive conditions such as esters (**2e**), ketones (**2m**), and iodine (**2i**) endured the established conditions. However, it is worth mentioning that the ester group was hydrolyzed under the strong basic conditions, with the exception of an α,β-unsaturated ester that could furnish **2e** in good yield. (5) The latent glycosyl donors SPTB ((*S*-2-(2-propylthio)benzyl, **2b**) and OPTB (*O*-2-(2-propylthio)benzyl, **2c**) featured in the interrupted Pummer reaction mediated (IPRm) glycosylations were well compatible [65,66,67,68,69], and could be transformed into the corresponding active SPSB/OPSB glycosyl donors via oxidation, indicating the potential for the further elongation of the sugar chain.

**Scheme 3:**
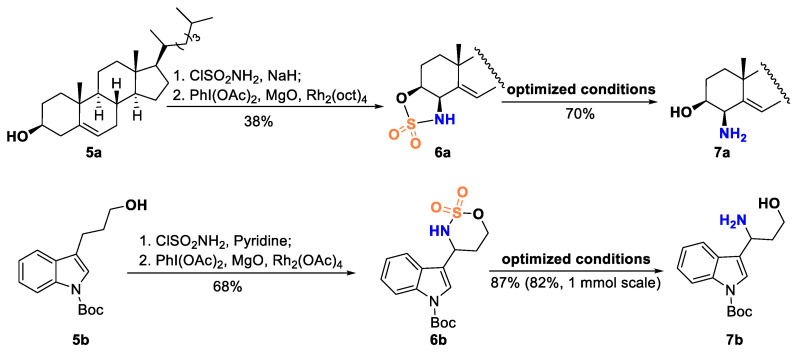
Amino alcohol synthesis through cyclic sulfamidate.

To verify the general synthetic utility of this protocol, we sought to merge the well-defined alcohol-induced amination reactions [31,32,33,34] with this deprotection protocol to modify naturally occurring or biologically important alcohols by introducing amino groups at nearby positions (Scheme 3). As an example, cholesterol was subjected to in-situ-generated sulfamoyl chloride [16,18], followed by Rh-catalyzed C-H amination conditions [32]. This sequence produced the five-membered cyclic sulfamidate **6a** in 38% yield. Subsequently, the application of the optimized deprotection conditions gave rise to the β-amino alcohol analog of cholesterol **7a** in 70% yield. The implementation of a similar protocol for the synthetic modification of indole-3-propanol also successfully introduced the amino group at the γ-position. To further showcase the applicability, a 1 mmol scale reaction of **6b** was performed under the optimized conditions. The desired amino alcohol **7b** was obtained in a comparable yield (82%).

## 3. Experimental Section

### 3.1. General

All the commercially available chemicals were purchased from Alfa, Innochem, and Adamas and used without further purification. The solvents for the reactions were dried on an Innovative Technologies Pure Solv400 solvent purifier. All the reactions were monitored using thin-layer chromatography over silica-gel-coated TLC plates (Yantai Chemical Industry Research Institute). The spots on the TLC were visualized by warming 10% H_2_SO_4_-(10% H_2_SO_4_ in ethanol) or 10% phosphomolybdic-acid (10% phosphomolybdic acid in ethanol) -sprayed plates on a hot plate. Column chromatography was performed using silica gel (Qingdao Marine Chemical Inc., Qingdao, China). NMR spectra were recorded with a Bruker AM-400 spectrometer (400 MHz) or Bruker Ascend TM-600 spectrometer (600 MHz). The ^1^H and ^13^C NMR chemical shifts were referenced against the solvent or solvent impurity peaks for CDCl_3_ at *δ*_H_ 7.24 and *δ*_C_ 77.23, for CD_2_Cl_2_ at *δ*_H_ 5.32 and *δ*_C_ 53.80, and for DMSO-*d*_6_ at *δ*_H_ 2.50 and *δ*_C_ 39.52 ppm, respectively. Optical rotations were measured at 25 °C with a Rudolph Autopol IV automatic polarimeter using a quartz cell with a 2 mL capacity and a 1 dm path length. Concentrations (c) are given in g/100 mL. High-resolution mass spectra were recorded with a Bruker micrOTOF II spectrometer using electrospray ionization (ESI). The copies of ^1^H and ^13^C NMR spectra of the new compounds are provided in the Supplementary Material.

### 3.2. Procedures for Compound ***3a*** and ***4a***

#### 3.2.1. Procedures for Compound **3a**

Ethyl 2-((3aS,4*S*,6*R*,7a*S*)-4-methyl-2,2-dioxido-6-(((2*R*,3*R*,4*S*,5*R*,6*S*)-3,4,5-tris(benzyloxy)-6-methoxytetrahydro-2*H*-pyran-2-yl)methoxy)tetrahydropyrano [4,3-*d*][1,2,3]oxathiazol-1(4*H*)-yl)-2-phenylacsetate (**3a**).

To a solution of **1a** (20.0 mg, 0.031 mmol) in THF (0.31 mL, C = 0.1 M), Ph(CO)CO_2_Et(5.3 μL, 0.034 mmol) and P(NMe_2_)_3_ (6.7 μL, 0.037 mmol) were added sequentially. After stirring for 45 min at room temperature, the mixture was concentrated and purified using silica gel chromatography to obtain **3a** (23.8 mg, 95%) as a colorless syrup. The major isomer: R*_f_* = 0.71 (petroleum ether-EtOAc 2:1). [α${]}_{D}^{25}$ −19.6 (c, 1.34 in CHCl_3_). The readings for ^1^H NMR spectra (400 MHz, CDCl_3_) were *δ* 7.37–7.25 (m, 18H, Ar-H), 7.14 (dd, *J* = 7.6, 2.8 Hz, 2H, Ar-H), 5.28 (s, 1H, CH), 4.98–4.95 (m, 2H, PhCH_2_, H-4′), 4.78 (d, *J* = 11.2 Hz, 3H, PhCH_2_), 4.66 (d, *J* = 12.0 Hz, 1H, PhCH_2_), 4.53 (d, *J* = 3.6 Hz, 2H, H-1, H-1′), 4.36–4.21 (m, 4H, CH_2_, PhCH_2_, H-3′), 4.02 (dq, *J* = 6.4, 1.6 Hz, 1H, H-5′), 3.93 (t, *J* = 9.2 Hz, 1H, H-3), 3.66 (d, *J* = 11.6 Hz, 1H, H-6a), 3.61 (dd, *J* = 10.4, 5.2 Hz, 1H, H-5), 3.46 (dd, *J* = 9.6, 3.6 Hz, 1H, H-2), 3.30 (dd, *J* = 10.8, 5.6 Hz, 1H, H-6b), 3.26–3.21 (m, 4H, H-4, OMe), 1.77–1.69 (m, 1H, H-2′a), 1.29–1.25 (m, 6H, H-6′, Me), and 1.05 (dd, *J* = 13.2, 6.4 Hz, 1H, H-2′b). The readings for the ^13^C NMR spectra (100 MHz, CDCl_3_) were *δ* 170.8, 138.9, 138.3, 133.6, 129.4, 129.3, 128.7, 128.6, 128.3, 128.2, 128.2, 128.0, 127.9, 127.9, 98.0, 96.8, 82.3, 81.5, 80.3, 78.1, 76.0, 75.2, 73.6, 70.1, 66.8, 62.7, 62.2, 61.2, 55.1, 53.0, 32.4, 16.8, and 14.2. The HRMS calculation for C_44_H_51_NO_12_S was [M + Na]^+^: 840.3024, found: 840.3041. The minor isomer: R*_f_* = 0.58 (petroleum ether-EtOAc 2:1). [α${]}_{D}^{25}$ −43.8 (c, 1.17 in CHCl_3_). The readings for the ^1^H NMR spectra (400 MHz, CDCl_3_) were *δ* 7.44–7.42 (m, 2H, Ar-H), 7.36–7.26 (m, 16H, Ar-H), 7.21–7.18 (m, 2H, Ar-H), 5.15 (s, 1H, CH), 4.97 (d, *J* = 10.8 Hz, 1H, PhCH_2_), 4.82 (d, *J* = 10.8 Hz, 1H, PhCH_2_), 4.80–4.75 (m, 3H, PhCH_2_, H-1′), 4.65 (d, *J* = 12.4 Hz, 1H, PhCH_2_), 4.51 (d, *J* = 3.6 Hz, 1H, H-1), 4.41 (d, *J* = 11.2 Hz, PhCH_2_), 4.26 (dd, *J* = 4.0, 1.2 Hz, 1H, H-4′), 4.23–4.12 (m, 2H, CH_2_), 3.92 (t, *J* = 9.2 Hz, 1H, H-3), 3.86 (qd, *J* = 6.4, 1.6 Hz, 1H, H-5′), 3.76 (ddd, *J* = 11.2, 6.4, 4.4 Hz, 1H, H-3′), 3.68 (dd, *J* = 10.4, 0.8 Hz, 1H, H-6a), 3.61 (dd, *J* = 10.4, 5.6 Hz, 1H, H-5), 3.41 (dd, *J* = 9.6, 3.2 Hz, 1H, H-2), 3.38 (dd, *J* = 10.8, 6.0 Hz, 1H, H-6b), 3.26 (t, *J* = 9.2 Hz, 1H, H-4), 3.23 (s, 3H, OMe), 2.23–2.16 (m, 1H, H-2′a), 1.98 (dd, *J* = 14.0, 6.4 Hz, 1H, H-2′b), and 1.22–1.18 (m, 6H, H-6′, Me). The readings for the ^13^C NMR spectra (100 MHz, CDCl_3_) were *δ* 168.9, 138.9, 138.3, 138.2, 133.3, 129.9, 129.3, 129.1, 128.7, 128.7, 128.6, 128.3, 128.2, 128.1, 128.0, 127.9, 98.0, 96.8, 82.3, 80.3, 80.2, 78.0, 76.0, 75.3, 73.5, 70.1, 66.6, 64.0, 62.6, 62.3, 55.2, 54.3, 30.8, 16.8, and 14.1. The HRMS calculation for C_44_H_51_NO_12_S was [M + Na]^+^: 840.3024, found: 840.3051.

#### 3.2.2. Procedures for Compound **4a**

(4a*S*,5*S*,7*R*,8a*S*)-5-methyl-2-phenyl-7-(((2*R*,3*R*,4*S*,5*R*,6*S*)-3,4,5-tris(benzyloxy)-6-methoxytetrahydro-2*H*-pyran-2-yl)methoxy)-4a,7,8,8a-tetrahydropyrano [3,4-*b*][1,4]oxazin-3(5*H*)-one (**4a**).

To a solution of **1a** (20.0 mg, 0.031 mmol) in THF (0.31 mL, C = 0.1 M), Ph(CO)CO_2_Et(5.3 μL, 0.034 mmol) and P(NMe_2_)_3_ (6.7 μL, 0.037 mmol) were added sequentially. After stirring for 45 min at room temperature, BTMG was added, and the mixture was stirred for 4 h at 65 °C. The mixture was concentrated and purified using silica gel chromatography to obtain **4a** (17.0 mg, 79%) as a white solid. R*_f_* = 0.61 (petroleum ether-EtOAc 2:1), m.p. 159–160 °C. [α${]}_{D}^{25}$ −128.3 (c, 1.36 in CHCl_3_). The readings for the ^1^H NMR spectra (400 MHz, CDCl_3_) were *δ* 7.95–7.82 (m, 2H, Ar-H), 7.47–7.26 (m, 18H, Ar-H), 5.00 (d, *J* = 11.2 Hz, 1H, PhCH_2_), 4.90 (d, *J* = 11.2 Hz, 1H, PhCH_2_), 4.84–4.77 (m, 3H, PhCH_2_, H-1), 4.66 (d, *J* = 12.0 Hz, 1H, PhCH_2_), 4.58 (d, *J* = 3.6 Hz, 1H, H-1′), 4.56 (d, *J* = 11.2 Hz, PhCH_2_), 4.52 (ddd, *J* = 10.8, 4.8, 3.2 Hz, 1H, H-3′), 4.29 (d, *J* = 1.6 Hz, 1H, H-4′), 4.07 (q, *J* = 6.4 Hz, 1H, H-5′), 4.01 (t, *J* = 9.2 Hz, 1H, H-3), 3.85 (dd, *J* = 10.8, 1.6 Hz, 1H, H-6a), 3.76 (ddd, *J* = 10.0, 4.8, 1.2 Hz, 1H, H-5), 3.55 (dd, *J* = 10.8, 5.2 Hz, 1H, H-6b), 3.52–3.46 (m, 2H, H-2, H-4), 3.38 (s, 3H, OMe), 2.15 (dd, *J* = 13.2, 4.8 Hz, 1H, H-2′a), 1.42 (td, *J* = 12.8, 3.2 Hz, 1H, H-2′b), and 1.33 (d, *J* = 6.4 Hz, 3H, H-6′). The readings for the ^13^C NMR spectra (100 MHz, CDCl_3_) were *δ* 158.8, 155.9, 138.9, 138.4, 138.4, 134.3, 131.4, 129.0, 128.7, 128.6, 128.5, 128.3, 128.2, 128.1, 127.8, 98.2, 96.7, 82.4, 80.3, 78.0, 75.9, 75.3, 75.0, 73.6, 70.2, 66.6, 63.6, 55.3, 51.5, 30.7, and 15.9. The HRMS calculation for C_42_H_45_NO_9_ [M + Na]^+^: 730.2987, found: 730.2989.

### 3.3. General Procedure for Deprotection and Characterization of the Products

To a solution of the substrate (1.0 equiv) in THF (C = 0.1 M), Ph(CO)CO_2_Et(1.1 equiv) and P(NMe_2_)_3_ (1.2 equiv) were added sequentially. After stirring for 45 min at room temperature, DBU (3.0 equiv relative to the starting substrate) and H_2_O (the amount of water was equal to that of the THF) were added sequentially, and the mixture was stirred for 4 h at 65 °C. The mixture was extracted with CH_2_Cl_2_ after removing the THF by concentration. The organic layer was washed with saturated NaHCO_3_ and brine, dried over Na_2_SO_4_, and concentrated in vacuo. The residue was purified with column chromatography on silica gel (dichloromethane-methanol gradient elution, with 0.5% or 1% NH_3_·H_2_O) to obtain the desired product.

(2*S*,3*S*,4*S*,6*R*)-4-amino-2-methyl-6-(((2*R*,3*R*,4*S*,5*R*,6*S*)-3,4,5-tris(benzyloxy)-6-methoxytetrahydro-2*H*-pyran-2-yl)methoxy)tetrahydro-2*H*-pyran-3-ol (**2a**).

According to the General Procedure, **1a** (20.0 mg, 0.031 mmol) was used to obtain **2a** as a white solid in 86% yield. R*_f_* = 0.43 (CH_2_Cl_2_-MeOH 10:1), m.p. 181–182 °C. [α${]}_{D}^{25}$ −29.6 (c, 0.45 in CHCl_3_).The readings for the ^1^H NMR spectra (400 MHz, CDCl_3_) were *δ* 7.27–7.24 (m, 4H, Ar-H), 7.23–7.14 (m, 11H, Ar-H), 4.89 (d, *J* = 10.8 Hz, 1H, PhCH_2_), 4.78 (d, *J* = 11.2 Hz, 1H, PhCH_2_), 4.71 (d, *J* = 10.8 Hz, 1H, PhCH_2_), 4.69 (d, *J* = 12.0 Hz, 1H, PhCH_2_), 4.64 (br s, 1H, H-1′), 4.56 (d, *J* = 12.0 Hz, PhCH_2_), 4.48 (d, *J* = 3.6 Hz, 1H, H-1), 4.44 (d, *J* = 11.2 Hz, 1H, PhCH_2_), 3.89 (t, *J* = 9.2 Hz, 1H, H-3), 3.77 (q, *J* = 6.4 Hz, 1H, H-5′), 3.72 (dd, *J* = 10.8, 1.6 Hz, 1H, H-6a), 3.65 (ddd, *J* = 10.0, 4.8, 1.2 Hz, 1H, H-5), 3.42–3.37 (m, 3H, H-2, H-4, H-6b), 3.28–3.25 (m, 4H, H-4′, OMe), 3.15–3.08 (m, 1H, H-3′), 1.52 (dd, *J* = 9.2, 2.4 Hz, 2H, H-2′a, H-2′b), and 1.11 (d, *J* = 6.8 Hz, 3H, H-6′). The readings for the ^13^C NMR spectra (100 MHz, CDCl_3_) were *δ* 138.9, 138.4, 138.4, 128.7, 128.6, 128.3, 128.2, 128.1, 128.0, 127.9, 98.1, 97.6, 82.3, 80.2, 78.1, 76.0, 75.2, 73.5, 71.1, 70.2, 66.3, 66.0, 55.3, 46.4, 32.8, and 17.1. The HRMS calculation for C_36_H_45_NO_9_ was [M + Na]^+^: 658.2987, found: 658.3006.

(2*S*,3*S*,4*S*,6*S*)-4-amino-6-(((2*R*,4a*R*,6*S*,7*R*,8*S*,8a*R*)-8-(benzyloxy)-6-((2-(isopropylthio)benzyl)thio)-2-phenylhexahydropyrano [3,2-*d*][1,3]dioxin-7-yl)oxy)-2-methyltetrahydro-2*H*-pyran-3-ol (**2b**).

According to the General Procedure, **1b** (20.0 mg, 0.027 mmol) was used to obtain **2b** as a white solid in 87% yield. R*_f_* = 0.13 (CH_2_Cl_2_-MeOH 15:1), m.p. 57–58 °C. [α${]}_{D}^{25}$ −121.5 (c, 0.61 in CHCl_3_). The readings for the ^1^H NMR spectra (400 MHz, CDCl_3_) were *δ* 7.46–7.41 (m, 3H, Ar-H), 7.36–7.34 (m, 3H, Ar-H), 7.30–7.25 (m, 6H, Ar-H), 7.21–7.14 (m, 2H, Ar-H), 5.54 (s, 1H, PhCHO_2_), 5.35 (d, *J* = 3.2 Hz, 1H, H-1′), 4.93 (d, *J* = 11.2 Hz, 1H, PhCH_2_), 4.60 (d, *J* = 11.2 Hz, 1H, PhCH_2_), 4.36–4.31 (m, 3H, H-1), 4.14 (d, *J* = 13.2 Hz, 1H, PhCH_2_S), 4.01 (d, *J* = 13.2 Hz, PhCH_2_S), 3.78–3.66 (m, 4H), 3.40–3.33 (m, 3H), 3.03 (d, *J* = 10.8 Hz, 1H), 1.61 (dd, *J* = 13.2, 4.0 Hz, 1H, H-2′a), 1.46–1.40 (m, 1H, H-2′b), 1.28–1.23 (m, 6H, (CH_3_)_2_CH), and 1.18 (d, *J* = 6.8 Hz, 3H, H-6′). The readings for the ^13^C NMR spectra (100 MHz, CDCl_3_) were *δ* 139.7, 138.1, 137.3, 135.5, 132.8, 130.0, 129.0, 128.5, 128.3, 128.0, 127.8, 127.7, 126.8, 126.0, 101.2, 98.4, 84.1, 84.0, 81.8, 75.1, 71.2, 70.0, 68.8, 66.9, 46.3, 38.8, 33.1, 29.8, 23.3, 23.1, and 16.8. The HRMS calculation for C_36_H_45_NO_7_S_2_ was [M + H]^+^: 668.2710, found: 668.2688.

(2*S*,3*S*,4*S*,6*S*)-4-amino-6-(((3a*R*,4*R*,6*S*,7*S*,7a*R*)-4-((2-(isopropylthio)benzyl)oxy)-2,2,6-trimethyltetrahydro-4*H*-[1,3]dioxolo [4,5-*c*]pyran-7-yl)oxy)-2-methyltetrahydro-2*H*-pyran-3-ol (**2c**). 

According to the General Procedure, **1c** (20.0 mg, 0.036 mmol) was used to obtain **2c** as a colorless syrup in 86% yield. R*_f_* = 0.30 (CH_2_Cl_2_-MeOH 10:1). [α${]}_{D}^{25}$ −93.9 (c, 1.20 in CHCl_3_).The readings for the ^1^H NMR spectra (400 MHz, CDCl_3_) were *δ* 7.43 (dd, *J* = 8.0, 1.6 Hz, 1H, Ar-H), 7.39 (dd, *J* = 7.2, 1.6 Hz, 1H, Ar-H), 7.27–7.24 (m, 1H, Ar-H), 7.23–7.19 (m, 1H, Ar-H), 5.45 (d, *J* = 2.4 Hz, 1H, H-1′), 5.07 (s, 1H, H-1), 4.85 (d, *J* = 12.0 Hz, 1H, PhCH_2_), 4.62 (d, *J* = 11.6 Hz, 1H, PhCH_2_), 4.19–4.15 (m, 1H), 4.12 (d, *J* = 5.2 Hz, 1H), 3.88 (q, *J* = 6.8 Hz, H-5′), 3.78–3.71 (m, 1H), 3.52 (dd, *J* = 10.0, 7.2 Hz, 1H, H-4), 3.40 (br s, 1H), 3.38–3.31 (m, 1H), 3.16 (br s, 1H), 1.73–1.61 (m, 4H, H-2′a, H-2′b, NH_2_), 1.52 (s, 3H, Me), 1.31 (s, 3H, Me), and 1.28–1.19 (m, 12H, H-6, H-6′, (CH_3_)_2_CH),. The readings for the ^13^C NMR spectra (100 MHz, CDCl_3_) were *δ* 138.8, 135.4, 132.8, 129.5, 128.5, 127.1, 109.5, 96.8, 95.6, 79.2, 76.5, 76.4, 71.4, 67.8, 66.5, 64.7, 46.5, 38.9, 33.1, 28.1, 26.7, 23.4, 23.3, 18.7, 18.2, and 17.2. The HRMS calculation for C_25_H_39_NO_7_S was [M + H]^+^: 498.2520, found: 498.2541.

(2*S*,3*S*,4*S*,6*R*)-4-amino-2-methyl-6-(((3a*R*,5*R*,5a*S*,8a*S*,8b*R*)-2,2,7,7-tetramethyltetrahydro-5*H*-bis([1,3]dioxolo)[4,5-*b*:4′,5’-*d*]pyran-5-yl)methoxy)tetrahydro-2*H*-pyran-3-ol (**2d**). 

According to the General Procedure, **1d** (15.5 mg, 0.034 mmol) was used to obtain **2d** as a colorless syrup in 92% yield. R*_f_* = 0.33 (CH_2_Cl_2_-MeOH 10:1). [α${]}_{D}^{25}$ −99.5 (c, 1.17 in CHCl_3_). The readings for the ^1^H NMR spectra (400 MHz, CDCl_3_) were *δ* 5.51 (d, *J* = 5.2 Hz, 1H, H-1), 4.88 (d, *J* = 2.8 Hz, 1H, H-1′), 4.58 (dd, *J* = 7.6, 2.4 Hz, 1H), 4.29 (dd, *J* = 5.2, 2.4 Hz, 1H), 4.21 (dd, *J* = 8.0, 1.6 Hz, 1H), 3.97–3.90 (m, 2H), 3.79 (dd, *J* = 10.0, 6.0 Hz, 1H), 3.54 (dd, *J* = 10.0, 6.8 Hz, 1H), 3.38 (d, *J* = 2.4 Hz, 1H), 3.21 (d, *J* = 8.8 Hz, 1H), 1.72–1.59 (m, 4H, H-2′a, H-2′b, NH_2_), 1.51 (s, 3H, Me), 1.42 (s, 3H, Me), 1.31 (s, 3H, Me), 1.30 (s, 3H, Me), and 1.23 (d, *J* = 6.4 Hz, 3H, H-6′). The readings for the ^13^C NMR spectra (100 MHz, CDCl_3_) were *δ* 109.5, 108.7, 97.3, 96.5, 71.5, 71.4, 70.9, 70.8, 67.0, 66.1, 65.6, 46.6, 33.0, 26.3, 26.2, 25.2, 24.8, and 17.2. The HRMS calculation for C_18_H_31_NO_8_ was [M + H]^+^: 390.2122, found: 390.2138.

Ethyl (E)-3-(3-(((2*S*,4*S*,5*S*,6*S*)-4-amino-5-hydroxy-6-methyltetrahydro-2*H*-pyran-2-yl)oxy)phenyl)acrylate (**2e**). 

According to the General Procedure, **1e** (12.0 mg, 0.031 mmol) was used to obtain **2e** as a colorless syrup in 74% yield. R*_f_*= 0.23 (CH_2_Cl_2_-MeOH 10:1). [α${]}_{D}^{25}$ −110.9 (c, 0.60 in CHCl_3_).The readings for the ^1^H NMR spectra (400 MHz, CDCl_3_) were *δ* 7.62 (d, *J* = 16.0 Hz, 1H, CH = CH), 7.27 (t, *J* = 7.6 Hz, 1H, Ar-H), 7.20 (br s, 1H, Ar-H), 7.14 (d, *J* = 7.6 Hz, 1H, Ar-H), 7.07 (dd, *J* = 8.4, 1.6 Hz, 1H, Ar-H), 6.39 (d, *J* = 16.0 Hz, 1H, CH = CH), 5.60 (br s, 1H, H-1), 4.24 (q, *J* = 7.2 Hz, 2H, CH_2_), 3.96 (q, *J* = 6.4 Hz, 1H, H-5), 3.48–3.44 (m, 2H, H-3, H-4), 1.86 (dd, *J* = 8.8, 2.4 Hz, 2H, H-2a, H-2b), 1.32 (t, *J* = 7.2 Hz, 3H, Me), and 1.22 (d, *J* = 6.8 Hz, 3H, H-6). The readings for the ^13^C NMR spectra (100 MHz, CDCl_3_) were *δ* 167.2, 157.5, 144.6, 136.1, 130.1, 121.9, 118.9, 118.4, 115.8, 96.1, 71.0, 67.2, 60.7, 46.4, 32.8, 17.2, and 14.5. The HRMS calculation for C_17_H_23_NO_5_ was [M + H]^+^: 322.1649, found: 322.1676.

(2*S*,3*S*,4*S*,6*R*)-4-amino-2-methyl-6-(((4*S*,5’*R*,6a*R*,6b*S*,8a*S*,8b*R*,9*S*,10*R*,11a*S*,12a*S*,12b*S*)-5’,6a,8a,9-tetramethyl-1,3,3’,4,4’,5,5’,6,6a,6b,6’,7,8,8a,8b,9,11a,12,12a,12b-icosahydrospiro[naphtho [2’,1’:4,5]indeno [2,1-*b*]furan-10,2’-pyran]-4-yl)oxy)tetrahydro-2*H*-pyran-3-ol (**2f**). 

According to the General Procedure, **1f** (20.0 mg, 0.033 mmol) was used to obtain **2f** as a white solid in 76% yield. R*_f_*= 0.32 (CH_2_Cl_2_-MeOH 10:1), m.p. 243–244 °C. [α${]}_{D}^{25}$ −154.8 (c, 1.09 in CHCl_3_). The readings for the ^1^H NMR spectra (400 MHz, CDCl_3_) were *δ* 5.31 (d, *J* = 5.2 Hz, 1H, C = CH), 5.00 (d, *J* = 3.2 Hz, 1H, H-1), 4.38 (q, *J* = 7.2 Hz, 1H), 3.94 (q, *J* = 6.4 Hz, 1H, H-5), 3.47–3.32 (m, 4H), 3.24 (d, *J* = 10.4 Hz, 1H), 2.32 (ddd, *J* = 13.2, 4.4, 1.6 Hz, 1H), 2.15 (td, *J* = 12.4, 2.4 Hz, 1H), 2.02–1.92 (m, 2H), 1.85–1.48 (m, 18H), 1.46–1.38 (m, 2H), 1.30–1.25 (m, 1H), 1.22 (d, *J* = 6.8 Hz, 3H, H-6), 1.17 (dd, *J* = 12.4, 4.4 Hz, 1H), 1.13–1.05 (m, 2H), 1.00 (s, 3H, Me), 0.96–0.89 (m, 4H), and 0.78–0.75 (m, 6H, Me). The readings for the ^13^C NMR spectra (100 MHz, CDCl_3_) were *δ* 140.9, 121.7, 109.5, 95.4, 81.0, 76.3, 71.5, 67.1, 66.1, 62.4, 56.7, 50.3, 46.6, 41.8, 40.5, 40.0, 38.9, 37.6, 37.1, 33.6, 32.3, 32.1, 31.7, 31.6, 30.5, 29.7, 29.0, 21.1, 19.6, 17.3, 17.2, 16.5, and 14.7. The HRMS calculation for C_33_H_53_NO_5_ was [M + H]^+^: 544.3997, found: 544.4011.

(2*S*,3*S*,4*S*,6*S*)-4-amino-2-methyl-6-(((2*R*,3*R*,5*R*,6*S*)-3,4,5-tris(benzyloxy)-6-methoxytetrahydro-2*H*-pyran-2-yl)methoxy)tetrahydro-2*H*-pyran-3-ol (**2g**). 

According to the General Procedure, **1g** (20.0 mg, 0.031 mmol) was used to obtain **2g** as a white solid in 72% yield. R*_f_* = 0.13 (CH_2_Cl_2_-MeOH 15:1). The readings for the ^1^H NMR spectra (600 MHz, CDCl_3_) were *δ* 7.31–7.18 (m, 15H, Ar-H), 4.91 (d, *J* = 11.4 Hz, 1H, PhCH_2_), 4.79 (d, *J* =10.8 Hz, 1H, PhCH_2_), 4.78 (d, *J* = 10.8 Hz, 1H, PhCH_2_), 4.73 (d, *J* = 12.0 Hz, 1H, PhCH_2_), 4.66 (d, *J* = 10.8 Hz, 1H, PhCH_2_), 4.60 (d, *J* = 12.6 Hz, 1H, PhCH_2_), 4.56 (d, *J* = 3.6 Hz, 1H, H-1), 4.40 (dd, *J* = 9.6, 1.8 Hz, 1H, H-1’), 4.10 (dd, *J* = 11.4, 3.6 Hz, 1H, H-6a), 3.92 (t, *J* = 9.6 Hz, 1H, H-3), 3.68–3.61 (m, 2H, H-5, H-6b), 3.54 (t, *J* = 9.6 Hz, 1H, H-4), 3.47 (dd, *J* = 9.6, 3.6 Hz, 1H, H-2), 3.42 (q, *J* = 6.6 Hz, 1H, H-5’), 3.33 (d, *J* = 1.8 Hz, 1H, H-4’), 3.30 (s, 3H, OMe), 2.97 (brs, 3H, NH_2_, OH), 2.92–2.87 (m, 1H, H-3’), 1.85–1.78 (m, 1H, H-2’a), 1.51–1.43 (m, 1H, H-2’b), and 1.19 (d, *J* = 6.6 Hz, 3H, H-6’). The readings for the ^13^C NMR spectra (150 MHz, CDCl_3_) were *δ* 139.0, 138.6, 138.4, 128.6, 128.6, 128.5, 128.3, 128.2, 128.1, 128.0, 127.9, 127.7, 100.4, 98.3, 82.2, 80.1, 77.8, 75.9, 75.2, 73.6, 71.9, 70.1, 70.1, 66.9, 55.3, 50.6, 34.4, and 17.1.

(2*S*,3*R*,4*R*,6*S*)-4-amino-6-(((2*R*,3*R*,4*S*,5*R*,6*S*)-4,5-bis(benzyloxy)-2-((benzyloxy)methyl)-6-methoxytetrahydro-2*H*-pran-3-yl)oxy)-2-methyltetrahydro-2H-pyran-3-ol (**2h**). 

According to the General Procedure, **1h** (21.0 mg, 0.032 mmol) was used to obtain **2h** as a colorless syrup in 62% yield. R*_f_* = 0.58 (CH_2_Cl_2_-MeOH 10:1). [α${]}_{D}^{25}$ −15.0 (c, 0.56 in CHCl_3_). The readings for the ^1^H NMR spectra (400 MHz, CDCl_3_) were *δ* 7.35–7.25 (m, 15H, Ar-H), 5.25 (d, *J* = 3.2 Hz, 1H, H-1’), 4.77 (d, *J* = 11.6 Hz, 2H, PhCH_2_), 4.69 (m, 2H, PhCH_2_, H-1), 4.65 (d, *J* = 11.6 Hz, 1H, PhCH_2_), 4.56 (d, *J* = 11.6 Hz, 1H, PhCH_2_), 4.65 (d, *J* = 11.6 Hz, 1H, PhCH_2_), 4.06 (d, *J* = 1.2 Hz, 1H, H-4), 3.92 (t, *J* = 10.0 Hz, 1H), 3.84 (qd, *J* = 10.0, 2.8 Hz, 2H), 3.61–3.56 (m, 3H), 3.38 (s, 3H, OMe), 3.06 (dd, *J* = 9.2, 4.4 Hz, 1H), 3.00 (m, 1H), 2.06–2.03 (m, 1H, H-2’a), 1.79 (dt, *J* = 14.4, 4.4 Hz, 1H, H-2’b), and 1.18 (d, *J* = 6.0 Hz, 3H, H-6’). The readings for the ^13^C NMR spectra (100 MHz, CDCl_3_) were *δ* 138.6, 138.5, 138.1, 128.7, 128.6, 128.6, 128.4, 128.0, 128.0, 127.8, 127.7, 99.0, 98.7, 78.9, 75.8, 74.8, 73.8, 73.5, 73.4, 71.0, 70.1, 69.6, 65.2, 55.6, 47.5, 36.4, and 18.2. The HRMS calculation for C_34_H_43_NO_8_ was [M + H]^+^: 594.3061, found: 594.3078.

(2*R*,3*R*,4*R*,6*R*)-4-amino-6-(((2*S*,6*S*,7*R*,8*R*,8a*S*)-7-iodo-6-methoxy-2-phenylhexahydropyrano [3,2-*d*][1,3]dioxin-8-yl)oxy)-2-methyltetrahydro-2-pyran-3-ol (**2i**). 

According to the General Procedure, **1i** (19.0 mg, 0.033 mmol) was used to obtain **2i** as a white solid in 79% yield. R*_f_* = 0.36 (CH_2_Cl_2_-MeOH 10:1), m.p. 89–90 °C. [α${]}_{D}^{25}$ +22.8 (c, 1.48 in CHCl_3_). The readings for the ^1^H NMR spectra (400 MHz, CDCl_3_) were *δ* 7.43–7.39 (m, 2H, Ar-H), 7.37–7.30 (m, 3H, Ar-H), 5.57 (s, 1H, PhCHO_2_), 5.18 (d, *J* = 3.2 Hz, 1H, H-1’), 5.05 (s, 1H, H-1), 4.36 (d, *J* = 4.4 Hz, 1H, H-2), 4.25 (dd, *J* = 9.6, 4.0 Hz, 1H, H-6a), 4.02–3.95 (m, 2H, H-4, H-5′), 3.90 (td, *J* = 10.0, 4.0 Hz, 1H, H-5), 3.84 (t, *J* = 10.0 Hz, 1H, H-6b), 3.48 (dd, *J* = 9.6, 4.4 Hz, 1H, H-3), 3.45 (br s, 1H, H-4’), 3.36 (s, 3H, OMe), 3.30 (ddd, *J* = 12.0, 4.8, 2.8 Hz, 1H, H-3’), 1.74 (dd, *J* = 13.2, 4.8 Hz, 1H, H-2’a), 1.65 (td, *J* = 12.4, 3.6 Hz, 1H, H-2’b), and 1.25 (d, *J* = 6.8 Hz, 3H, H-6’). The readings for the ^13^C NMR spectra (100 MHz, CDCl_3_) were *δ* 137.5, 129.2, 128.4, 126.2, 104.0, 101.8, 99.3, 80.8, 71.6, 70.9, 68.9, 67.4, 64.8, 55.3, 46.5, 35.2, 32.6, and 17.2. The HRMS calculation for C_20_H_28_INO_7_ was [M + H]^+^: 522.0983, found: 522.0996.

(2*R*,3*R*,4*R*,6*S*)-4-amino-6-(((3*S*,8*S*,9*S*,10*R*,13*R*,14*S*,17*R*)-10,13-dimethyl-17-((*R*)-6-methylheptan-2-yl)-2,3,4,7,8,9,10,11,12,13,14,15,16,17-tetradecahydro-1*H*-cyclopenta[a]phenanthren-3-yl)oxy)-2-methyltetrahydro-2*H*-pyran-3-ol (**2j**). 

According to the General Procedure, **1j** (24.0 mg, 0.042 mmol) was used to obtain **2j** as a white solid in 81% yield. R*_f_* = 0.26 (CH_2_Cl_2_-MeOH 10:1), m.p. 185–186 °C. [α${]}_{D}^{25}$ +54.0 (c, 1.62 in CHCl_3_). The readings for the ^1^H NMR spectra (400 MHz, CDCl_3_) were *δ* 5.32 (d, *J* = 4.8 Hz, 1H, C = CH), 4.99 (d, *J* = 3.2 Hz, 1H, H-1), 3.96 (q, *J* = 6.4 Hz, 1H, H-5), 3.44 (m, 1H,), 3.39 (d, *J* = 2.4 Hz, 1H, H-4), 3.25 (ddd, *J* = 11.6, 4.4, 2.8 Hz, 1H, H-3), 2.32–2.21 (m, 2H), 2.00–1.92 (m, 2H), 1.84–1.77 (m, 4H), 1.71–1.63 (m, 2H), 1.61–1.37 (m, 4H), 1.51–1.36 (m, 5H), 1.36–1.28 (m, 3H), 1.23 (d, *J* = 6.4 Hz, 4H, H-6), 1.20–0.99 (m, 8H), 0.98–0.92 (m, 5H), 0.89 (d, *J* = 6.4 Hz, 3H, Me), 0.85 (d, *J* = 1.6 Hz, 3H, Me), 0.83 (d, *J* =1.6 Hz, 3H, Me), and 0.65 (s, 3H, Me). The readings for the ^13^C NMR spectra (100 MHz, CDCl_3_) were *δ* 141.2, 121.9, 95.2, 76.3, 71.4, 66.1, 57.0, 56.4, 50.4, 46.6, 42.6, 40.4, 40.0, 39.7, 37.3, 37.0, 36.4, 36.0, 33.6, 32.2, 32.1, 28.4, 28.2, 28.1, 24.5, 24.1, 23.0, 22.8, 21.3, 19.6, 18.9, 17.2, and 12.1. The HRMS calculation for C_33_H_57_NO_3_ was [M + H]^+^: 516.4411, found: 516.4422.

(2*S*,3*S*,4*S*,6*S*)-4-amino-6-(4-methoxynaphthalen-1-yl)-2-methyltetrahydro-2*H*-pyran-3-ol (**2k**). 

According to the General Procedure, **1k** (20.0 mg, 0.057 mmol) was used to obtain **2k** as a white solid in 78% yield. R*_f_* = 0.29 (CH_2_Cl_2_-MeOH 10:1), m.p. 112–113 °C. [α${]}_{D}^{25}$ −86.6 (c, 0.56 in CHCl_3_). The readings for the ^1^H NMR spectra (400 MHz, CDCl_3_) were *δ* 8.28 (d, *J* = 8.0 Hz, 1H, Ar-H), 7.98 (d, *J* = 8.4 Hz, 1H, Ar-H), 7.53–7.43 (m, 3H, Ar-H), 6.76 (d, *J* = 8.0 Hz, 1H, Ar-H), 5.00 (d, *J* = 10.4 Hz, 1H, H-1), 3.97 (s, 3H, OMe), 3.82 (q, *J* = 6.4 Hz, 1H, H-5), 3.52 (br s, 1H, H-4), 3.18 (d, *J* = 10.4 Hz, 1H, H-3), 1.96–1.84 (m, 2H, H-2a, H-2b), and 1.39 (d, *J* = 6.4 Hz, 3H, H-6). The readings for the ^13^C NMR spectra (100 MHz, CDCl_3_) were *δ* 155.5, 131.8, 129.3, 126.8, 126.0, 125.1, 123.6, 123.3, 122.8, 103.3, 75.7, 75.6, 71.5, 55.7, 52.2, 35.7, and 17.9. The HRMS calculation for C_17_H_21_NO_3_ was [M + H]^+^: 288.1594, found: 288.1597.

(2*S*,3*S*,4*S*,6*S*)-4-amino-6-(6-hydroxy-2,3,4-trimethoxyphenyl)-2-methyltetrahydro-2*H*-pyran-3-ol (**2l**). 

According to the General Procedure, **1l** (20.0 mg, 0.053 mmol) was used to obtain **2l** as a white solid in 77% yield. R*_f_* = 0.24 (CH_2_Cl_2_-MeOH 10:1), m.p. 178–179 °C. [α${]}_{D}^{25}$ −71.0 (c, 0.67 in CHCl_3_). The readings for the ^1^H NMR spectra (400 MHz, CDCl_3_) were *δ* 6.23 (s, 1H, Ar-H), 4.90 (dd, *J* = 11.6, 2.8 Hz, 1H, H-1), 3.86 (s, 3H, OMe), 3.78 (s, 3H, OMe), 3.74 (s, 3H, OMe), 3.65 (q, *J* = 6.4 Hz, 1H, H-5), 3.45 (d, *J* = 2.4 Hz, 1H, H-4), 3.16 (d, *J* = 10.4 Hz, 1H, H-3), 1.89 (q, *J* = 12.0 Hz, 1H, H-2a), 1.61 (dq, *J* = 13.2, 3.2 Hz, 1H, H-2b), and 1.36 (d, *J* = 6.4 Hz, 3H, H-6). The readings for the ^13^C NMR spectra (100 MHz, CDCl_3_) were *δ* 153.7, 152.4, 150.1, 135.1, 111.5, 97.3, 75.3, 73.6, 69.8, 61.5, 61.1, 56.1, 50.7, 33.9, and 17.8. The HRMS calculation for C_15_H_23_NO_6_ was [M + H]^+^: 314.1598, found: 314.1625.

5-((2*S*,4*S*,5*S*,6*S*)-4-amino-5-hydroxy-6-methyltetrahydro-2*H*-pyran-2-yl)-9-hydroxy-8-methoxy-3,4-dihydroanthracen-1(2*H*)-one (**2m**). 

According to the General Procedure, **1m** (15.0 mg, 0.035 mmol) was used to obtain **2m** as a yellow solid in 91% yield. R*_f_* = 0.27 (CH_2_Cl_2_-MeOH 10:1), m.p. 166–167 °C. [α${]}_{D}^{25}$ −154.0 (c, 0.82 in CHCl_3_). The readings for the ^1^H NMR spectra (400 MHz, CDCl_3_) were *δ* 7.66 (d, *J* = 8.4 Hz, 1H, Ar-H), 7.11 (s, 1H, Ar-H), 6.77 (d, *J* = 8.4 Hz, 1H, Ar-H), 4.87 (d, *J* = 10.4 Hz, 1H, H-1), 3.98 (s, 3H, OMe), 3.80 (q, *J* = 6.4 Hz, 1H, H-5), 3.52 (d, *J* = 2.0 Hz, 1H, H-4), 3.18 (dt, *J* = 11.6, 3.6 Hz, 1H, H-3), 2.99 (t, *J* = 6.0 Hz, 2H, CH_2_), 2.74 (t, *J* = 6.4 Hz, 2H, CH_2_), 2.13–2.06 (m, 2H, CH_2_), 1.91 (dd, *J* = 13.2, 2.4 Hz, 1H, H-2a), 1.81–1.72 (m, 3H, H-2b, NH_2_), and 1.39 (d, *J* = 6.8 Hz, 3H, H-6). The readings for the ^13^C NMR spectra (100 MHz, CDCl_3_) were *δ* 204.8, 166.5, 159.9, 139.5, 137.3, 129.1, 128.6, 115.5, 112.0, 111.8, 105.3, 75.7, 75.4, 71.4, 56.4, 52.1, 39.2, 35.6, 31.0, 23.0, and 17.9. The HRMS calculation for C_21_H_25_NO_5_ was [M + H]^+^: 372.1805, found: 372.1790.

(3*S*,4*R*,8*S*,9*S*,10*R*,13*R*,14*S*,17*R*)-4-amino-10,13-dimethyl-17-((*R*)-6-methylheptan-2-yl)-2,3,4,7,8,9,10,11,12,13,14,15,16,17-tetradecahydro-1*H*-cyclopenta[*a*]phenanthren-3-ol (**7a**). 

To a solution of **6a** (23.0 mg, 0.050 mmol, 1.0 equiv) in THF (0.5 mL), Ph(CO)CO_2_Et(0.055 mmol, 1.1 equiv) and P(NMe_2_)_3_ (0.060 mmol, 1.2 equiv) were added sequentially. After stirring for 45 min at room temperature, DBU (0.200 mmol, 4.0 equiv) and H_2_O (0.5 mL) were added sequentially, and the mixture was stirred for 4 h at 65 °C. The mixture was extracted with CH_2_Cl_2_ after removing the THF by concentration. The organic layer was washed with saturated NaHCO_3_ and brine, dried over Na_2_SO_4_, and concentrated in vacuo. The crude residue was purified using column chromatography on silica gel (dichloromethane-methanol gradient elution, with 1% NH_3_·H_2_O) to obtain **7a** as a white solid in 70% yield. R*_f_* = 0.40 (CH_2_Cl_2_-MeOH 10:1), m.p. 158–159℃. [α${]}_{D}^{25}$ −41.3 (c, 0.76 in CHCl_3_). The readings for the ^1^H NMR spectra (400 MHz, CDCl_3_) were *δ* 5.54 (s, 1H, C = CH), 3.52–3.46 (m, 2H), 2.42 (br s, 3H, OH, NH2), 2.06–1.96 (m, 2H), 1.84–1.72 (m, 3H), 1.65–1.50 (m, 4H), 1.42–1.23 (m, 8H), 1.13–1.03 (m, 12H), 0.89 (d, *J* = 6.4 Hz, 3H, Me), 0.85 (d, *J* = 1.6 Hz, 3H, Me), 0.83 (d, *J* = 1.6 Hz, 3H, Me), and 0.65 (s, 3H, Me). The readings for the ^13^C NMR spectra (100 MHz, CDCl_3_) were *δ* 127.2, 71.5, 57.3, 56.3, 50.5, 42.5, 39.9, 39.7, 36.8, 36.4, 36.3, 36.0, 32.4, 32.1, 28.4, 28.2, 25.9, 24.5, 24.1, 23.0, 22.8, 21.9, 20.7, 18.9, and 12.1. The HRMS calculation for C_27_H_47_NO was [M + H]^+^: 402.3730, found: 402.3717.

*tert*-butyl 3-(1-amino-3-hydroxypropyl)-1*H*-indole-1-carboxylate (**7b**).

To a solution of **6b** (20.0 mg, 0.079 mmol, 1.0 equiv) in THF (0.79 mL), Ph(CO)CO_2_Et(0.158 mmol, 2.0 equiv) and P(NMe_2_)_3_ (0.166 mmol, 2.1 equiv) were added sequentially. After stirring for 45 min at room temperature, DBU (0.474 mmol, 6.0 equiv) and H_2_O (0.79 mL) were added sequentially, and the mixture was stirred for 4 h at 65 °C. The mixture was extracted with CH_2_Cl_2_ after removing the THF by concentration. The organic layer was washed with saturated NaHCO_3_ and brine, dried over Na_2_SO_4_, and concentrated in vacuo. The crude residue was purified using column chromatography on silica gel (dichloromethane-methanol gradient elution, with 1% NH_3_·H_2_O) to obtain **7b** as a yellow oil in 87% yield. According to the above procedure, **6b** (352.4 mg, 1.0 mmol) was used to obtain **7b** as a yellow oil in 82% yield. R*_f_* = 0.25 (CH_2_Cl_2_-MeOH 10:1). The readings for the ^1^H NMR spectra (400 MHz, CDCl_3_) were *δ* 8.12 (d, *J* = 7.2 Hz, 1H, Ar-H), 7.54 (d, *J* = 8.4 Hz, 2H, Ar-H, C = CH), 7.30 (t, *J* = 7.2 Hz, 1H, Ar-H), 7.21 (t, *J* = 7.2 Hz, 1H, Ar-H), 4.47 (dd, *J* = 7.6, 4.0 Hz, 1H), 3.84 (t, *J* = 5.2 Hz, 2H), 3.18 (s, 3H, OH, NH_2_), 2.10–1.98 (m, 2H), and 1.64 (s, 9H, (CH_3_)_3_C). The readings for the ^13^C NMR spectra (100 MHz, CDCl_3_) were *δ* 149.9, 136.0, 128.7, 124.9, 122.8, 122.0, 119.2, 115.8, 84.1, 62.2, 48.9, 37.8, and 28.4. The HRMS calculation for C_16_H_22_N_2_O_3_ was [M + H]^+^: 291.1703, found: 291.1691.

## 4. Conclusions

In conclusion, the investigation described above has led to the development of a practical method to smoothly convert cyclic sulfamidates into amino alcohols under mild conditions. This highly efficient deprotection method is initiated with the Kukhtin–Ramirez reaction. It exhibited operational simplicity, which provided a solution to the deprotection problem encountered in our synthesis of rare amino sugar. In addition, this approach allows the construction of valuable building blocks and structurally complex compounds containing amino alcohol motifs.

## Figures and Tables

**Table 1:** Reaction development.

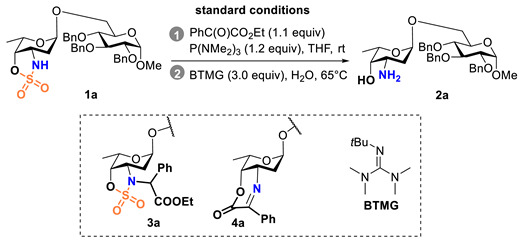		
Entry	Variation from Standard Conditions	Yield *^a^*
1	Step 1 only	**3a**, 95%
2	Without H_2_O	**4a**, 79%
3	Standard conditions	**2a**, 88%
4	DBU instead of BTMG	**2a**, 86%
5	KOH instead of BTMG	**2a**, 80%
6	K_2_CO_3_ instead of BTMG	**2a**, 16%
7	Et_3_N instead of BTMG	**2a**, trace
8	Ambersep^®^ 900 (OH) instead of BTMG	**2a**, 80%

*^a^* Yield of isolated product.

## Data Availability

Data are included within the manuscript or the Supplementary Data File.

## Supplementary Materials

The following supporting information can be downloaded at https://www.mdpi.com/article/10.3390/molecules28010182/s1: synthesis of compounds **1a**–**1m**, **6a**–**6b**, and 1,2-dicarbonyl reagents (**S3**–**S5**) [70,71]; Table S1: screening of 1,2-dicarbonyl compounds; ^1^H NMR spectra for **6a**–**6b** and **S3**–**S5**; ^1^H and ^13^C NMR spectra for compounds **2a**–**2m**, **3a**, and **4a**; 2D HSQC and COSY NMR spectra for compounds **2a**, **3a**, and **4a**.
