# Supplementary material for: Kukhtin–Ramirez-Reaction-Inspired Deprotection of Sulfamidates for the Synthesis of Amino Sugars
Source: Molecules. 2022 Dec 25;28(1):182. doi: 10.3390/molecules28010182 (PMC9822045; doi:10.3390/molecules28010182)
Supplement: Supplementary file 1 [file molecules-28-00182-s001.zip › molecules-2100361-supplementary.pdf]

Supporting Information

Kukhtin-Ramirez Reaction Inspired Deprotection of Sulfamidates for the Synthesis of Amino Sugars

Ting Li [1,#], Bingbing Xu [1,#], Dengxian Fu [1], Qian Wan [1], and Jing Zeng [1,2]*

[1] Hubei Key Laboratory of Natural Medicinal Chemistry and Resource Evaluation, School of Pharmacy, Huazhong University of Science and Technology, Wuhan 430030, China

[2] Shandong Provincial Key Laboratory of Carbohydrate Chemistry and Glycobiology, Shandong University, Qingdao 266237, China

[#] T.L. and B.X. contributed equally

* Corresponding Authors

Table of Contents

1. Preparation of Disaccharides 1a-1m……….…………………..……..…..…..S2

2. Preparation of non-sugar substrates 6a-6b………...………………….…..…..S2

3. Preparation of 1,2-dicarbonyl compounds S3-S5…….………….………........S4

4. Screening of other1,2-dicarbonyl compounds...…S6

5. Reference………………………………………………………….…….…...S7

6. Copies of NMR spectra…………………………………..……..………..S8

1. Preparation of Disaccharides 1a-1m

Disaccharides **1a-1m** were prepared according to our previously reported procedures and their analytical data were in accordance with that previously described [1].

2. Preparation of non-sugar substrates 6a-6b

Preparation of (-)-4,5β-(cholest-5-enyl)-1,2,3-oxathiazole-2,2-dioxide (6a)

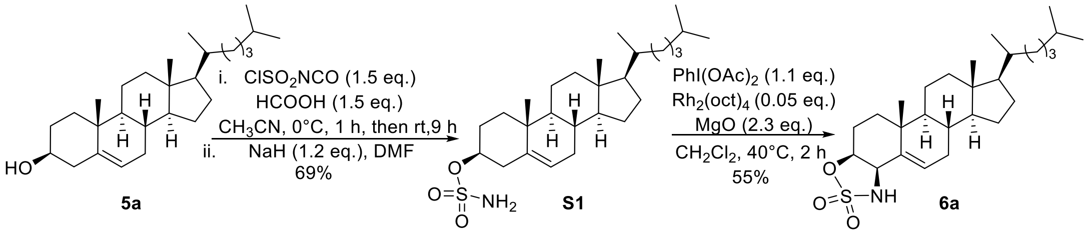

Preparation of $ClSO_2NH_2$ solution (2 M in MeCN): Formic acid (392 μL, 4.50 mmol) was added dropwise to neat $ClSO_2NCO$ (170 μL, 4.50 mmol) at 0 °C with rapid stirring. After vigorously stirring for 5 min at 0 °C, MeCN (2.3 mL, C = 2.0 M) was added, and the reaction stirred for 1 h at 0 °C then room temperature overnight.

Sulfamate ester formation: A 50 mL round-bottom flask equipped with stir bar and rubber septum was charged with 60% NaH (144.0 mg, 3.60 mmol) and DMF (3.0 mL, C = 1.0 M) and cooled to 0 °C. A solution of **5a** (1.2 g, 3.00 mmol) in DMF (2.4 mL, C = 1.25 M) was slowly added. The reaction was stirred at room temperature for 1 h, after which it was cooled again to 0 °C. The freshly prepared solution of $ClSO_2NH_2$ in CH_3CN was then added dropwise via syringe, and the reaction was stirred at room temperature. Upon complete consumption of starting material as monitored by TLC, the reaction was quenched with H_2O until the mixture turned clear and then extracted with EtOAc. The organic layer was washed with water and brine, dried over Na_2SO_4 and concentrated *in vacuo*. The residue was purified by silica gel chromatography to give **S1** (960.0 mg, 69%) as a white solid.

Intramolecular amination: To a solution of **S1** (400.0 mg, 0.86 mmol) in CH_2Cl_2 (5.4 mL, C = 0.16 M), MgO (79.8 mg, 1.98 mmol), $PhI(OAc)_2$ (306.0 mg, 0.95 mmol),

and $Rh_2(oct)_4$ (33.4 mg, 0.043 mmol) were added sequentially. The suspension was stirred vigorously for 2 h at 40 °C. The reaction was cooled to room temperature, diluted with CH_2Cl_2 and filtered through a pad of Celite. The filter cake was rinsed with CH_2Cl_2 and the combined filtrates were evaporated under reduced pressure. The residue was purified by silica gel chromatography to give **6a** (222.0 mg, 55%) as a white solid. R_f = 0.29 (petroleum ether-EtOAc 5:1). ^1H NMR (400 MHz, $CDCl_3$) δ 5.80 (app. t, J = 2.4 Hz, 1H), 4.72 (dt, J = 10.8, 6.0 Hz, 1H,), 4.50 (t, J = 5.6 Hz, 1H), 4.30 (d, J = 4.8 Hz, 1H), 2.25 (dq, J = 14.0, 3.2 Hz, 1H), 2.12 (dt, J = 18.0, 4.4 Hz, 1H), 2.06-1.99 (m, 2H), 1.89 (dt, J = 14.0, 4.0 Hz, 1H), 1.85-1.77 (m, 1H), 1.64-1.43 (m, 7H), 1.41-1.22 (m, 5H), 1.18 (s, 3H), 1.15-1.03 (m, 6H), 1.00-0.93 (m, 2H), 0.89 (d, J = 6.4 Hz, 3H), 0.84 (dd, J = 6.4, 1.6 Hz, 6H), 0.67 (s, 3H). Analytical data for **6a** was essentially the same as those reported previously [2].

Preparation of 3-(N-Boc-Indolyl)-1,2,3-oxathiazole-2,2-dioxide (6b)

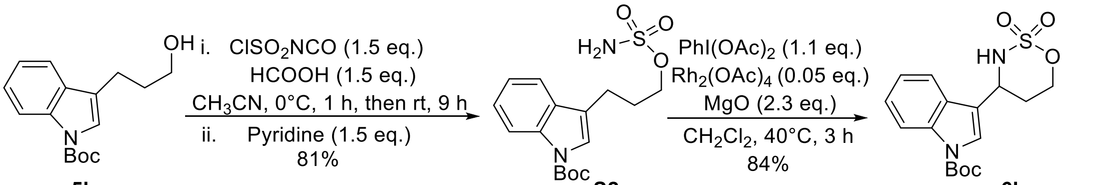

Formic acid (0.30 mL, 8.13 mmol) was added dropwise to neat $ClSO_2NCO$ (0.70 μL, 8.13 mmol) at 0 °C with rapid stirring. After vigorously stirring for 5 min at 0 °C, CH_2Cl_2 (8.1 mL, C = 1.0 M) was added, and the reaction stirred for 1 h at 0 °C then room temperature overnight. After cooled to 0 °C, a solution of **5b** (1.49 g, 5.42 mmol) and pyridine (0.65 mL, 8.13 mmol) in CH_2Cl_2 (10.8 mL, C = 0.5 M) was slowly added. The reaction was then stirred at room temperature. Upon complete consumption of starting material as monitored by TLC, the reaction was quenched with H_2O until the mixture turned clear and then extracted with EtOAc. The organic layer was washed with water and brine, dried over Na_2SO_4 and concentrated *in vacuo*. The residue was purified by silica gel chromatography to give **S2** (1.56 g, 81%) as a white solid.

To a solution of **S2** (886.0 mg, 2.5mmol) in CH_2Cl_2 (12.5 mL, C = 0.2 M), MgO (231.7 mg, 7.75 mmol), $PhI(OAc)_2$ (913.3 mg, 2.75 mmol), and $Rh_2(OAc)_4$ (55.0 mg, 0.125 mmol) were added sequentially. The suspension was stirred vigorously for 2 h at 40 °C. The reaction was cooled to room temperature, diluted with CH_2Cl_2, and filtered through a pad of Celite. The filter cake was rinsed with CH_2Cl_2 and the combined filtrates were evaporated under reduced pressure. The residue was purified by silica gel chromatography to give **6b** (739.0 mg, 84%) as a white solid. White solid. R_f = 0.46 (petroleum ether-EtOAc 3:1). ^{1}H NMR (400 MHz, $CDCl_3$) δ 8.10 (d, J = 8.0 Hz, 1H, Ar-H), 7.70 (d, J = 7.6 Hz, 1H, Ar-H), 7.54 (s, 1H, C=CH), 7.37-7.32 (m, 1H, Ar-H), 7.28-7.26 (m, 1H, Ar-H), 5.14 (td, J = 11.2, 2.0 Hz, 1H), 4.92 (td, J = 12.4, 2.4 Hz, 1H), 4.65 (ddd, J = 11.6, 4.8, 1.6 Hz, 1H), 4.25 (d, J = 10.0 Hz, 1H), 2.42-2.29 (m, 1H), 2.16-2.11 (m, 1H), 1.65 (s, 9H, $C(CH_3)_3$). Analytical data for **6b** was essentially the same as those reported previously [3].

3. Preparation of 1,2-dicarbonyl compounds

Preparation of N,N-diethyl-2-oxo-2-phenylacetamide (S3)

To a solution of phenylglyoxylic acid (100 mg, 0.67 mmol) and DABCO (75.2 mg, 0.67 mmol) in dry DCE (6.7 mL, C = 0.1 M) was added diethylcarbamyl chloride (127 μL, 1.00 mmol). The reaction mixture was stirred at 60 °C for 12 h. After the reaction was completed, the mixture was neutralized with saturated Na_2CO_3 and then extracted with CH_2Cl_2. The organic layer was washed with water and brine, dried over Na_2SO_4 and concentrated *in vacuo*. The residue was purified by silica gel chromatography to give **S3** (129 mg, 94%) as yellow oil. R_f = 0.33 (petroleum ether-EtOAc 2:1). ^{1}H NMR (400 MHz, $CDCl_3$) δ 7.93-7.90 (m, 2H, Ar-H), 7.63-7.60 (m, 1H, Ar-H), 7.51-7.46 (m, 2H, Ar-H), 3.54 (q, J = 7.2 Hz, 2H, CH_2), 3.22 (q, J = 7.2 Hz, 2H, CH_2), 1.27 (t, J = 7.2 Hz, 3H, Me), 1.14 (t, J = 7.2 Hz, 3H, Me) Analytical data for **S3** were essentially the same as those reported previously [4].

1-Methyl-1H-indole-2,3-dione (S4)

Isatin (100.0 mg, 0.68 mmol) was dissolved in DMF (1.4 mL, C = 0.5 M) at 0 °C, and sodium hydride (32.6 mg, 0.82 mmol, 60%) was added slowly into the mixture. After stirred for 30 min, CH_3I (50 μL, 0.82 mmol) was added slowly, and the mixture was stirred for 2 h at 0 °C and then 1 h at room temperature. After being quenched with saturated $NaHCO_3$, the mixture was extracted with EtOAc. The organic layer was washed with water and brine, dried over Na_2SO_4 and concentrated *in vacuo*. The residue was purified by silica gel chromatography to give **S4** (107 mg, 98%) as a red solid. R_f = 0.33 (petroleum ether-EtOAc 2:1). ^{1}H NMR (400 MHz, $CDCl_3$) δ 7.60-7.54 (m, 2H, Ar-H), 7.09 (t, J = 7.6 Hz, 1H, Ar-H), 6.87 (d, J = 8.0 Hz, 1H, Ar-H), 3.22 (s, 3H, Me). Analytical data for **S4** were essentially the same as those reported previously [5].

1-Tosylindoline-2,3-dione (S5)

Isatin (100.0 mg, 0.68 mmol) was dissolved in CH_2Cl_2 (1.0 mL, C = 0.7 M) at 0 °C under Ar atmosphere, and Et_3N (104 μL, 0.75 mmol) was added. After stirred for 20 min, tosyl chloride (129.6 mg, 0.68 mmol) was added, and the mixture was stirred for 1 h at 0 °C and then 3 h at room temperature. The solvent was removed under reduced pressure, and the residue was washed with CH_3OH to give N-Tosyl protected isatin **S5** (103 mg, 50%) as a yellow solid. R_f= 0.23 (petroleum ether-acetone 3:1). ^{1}H NMR (400 MHz, DMSO-d_6) δ 7.97 (d, J = 8.4 Hz, 2H, Ar-H), 7.86 (d, J = 8.4 Hz, 1H, Ar-H), 7.76 (td, J = 7.6, 1.2 Hz, 1H, Ar-H), 7.68 (dd, J = 7.6, 1.2 Hz, 1H, Ar-H), 7.48 (d, J = 8.0 Hz, 2H, Ar-H), 7.32 (td, J = 7.6, 0.8 Hz, 1H, Ar-H), 2.40 (s, 3H, Me). Analytical data for **S5** were essentially the same as those reported previously [6].

4. Screening of other 1,2-dicarbonyl compounds

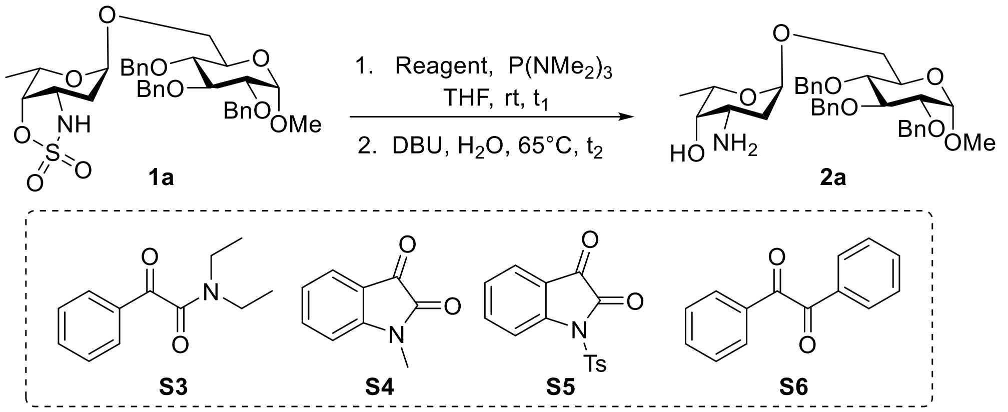

Entry	**Reagent**	$P(NMe_2)_3$	t_1	DBU	t_2	Yield of **2b**
1	**S3** (1.1 eq.)	1.2 eq.	1.5 h	/	/	N.R.
2	**S4** (1.1 eq.)	1.2 eq.	1.5 h	/	/	N.R.
3	**S5** (1.1 eq.)	1.2 eq.	1.5 h	/	/	N.R.
4	**S6** (3.0 eq.)	3.2 eq.	8 h	3.0 eq.	overnight	<10%

The reaction with 1,2-dicarbonyl compounds **S3**-**S5** furnished Kukhtin-Ramirez adducts, but these adducts did not react with **1a** to produce N-H insertion products in the first step. In these several cases, sugar **1a** was fully recovered.

5. Reference

[1] (a) Zeng, J.; Wang, R.; Yao, W.; Zhang, S.; Sun, G.; Liao, Z.; Meng, L.; Wan, Q. Diversified synthesis and α-selective glycosylation of 3-amino-2,3,6-trideoxy sugars. *Org. Chem. Front.* **2018**, *5*, 3391-3395. (b) Fu, D.; Zhang, S.; Xu, B.; Peng, P.; Wan, Q.; Zeng, J. Selective reduction leading to 3,5-*cis*-3-aminosugars: Synthesis and stereoselective glycosylation. *J. Org. Chem*. **2022**. DOI: 10.1021/acs.joc.2c02364 (accepted).

[2] Paradine, S. M.; White, M. C. Iron-catalyzed intramolecular allylic C-H amination. *J. Am. Chem.Soc.* **2012**, *134*, 2036-2039.

[3] Espino, C. G.; When, P. M.; Chow, J.; Du Bois, J. Synthesis of 1,3-difunctionalized amine derivatives through selective C-H bond oxidation. *J. Am. Chem. Soc.* **2001**, *123*, 6935-6936.

[4] Zhang, X.; Wang, L. TBHP/I_2-promoted oxidative coupling of acetophenones with amines at room temperature under metal-free and solvent-free conditions for the synthesis of α-ketoamides. *Green Chem.* **2012**, *14*, 2141-2145.

[5] Ryu, H.; Seo, J.; Ko, H. M. Synthesis of Spiro[oxindole-3,2′-pyrrolidine] derivatives from benzynes and azomethine ylides through 1,3-dipolar cycloaddition reactions. *J. Org. Chem.* **2018**, *83*, 14102-14109.

[6] Yu, T. T.; Nizalapur, S.; Ho, K. K. K.; Yee, E.; Berry, T.; Cranfield, C. G.; Willcox, M.; Black, D. S.; Kumar, N. Design, synthesis and biological evaluation of N-sulfonylphenyl glyoxamide-based antimicrobial peptide mimics as novel antimicrobial agents. *Chemistryselect* **2017**, *2*, 3452-3461.

6. Copies of NMR spectra

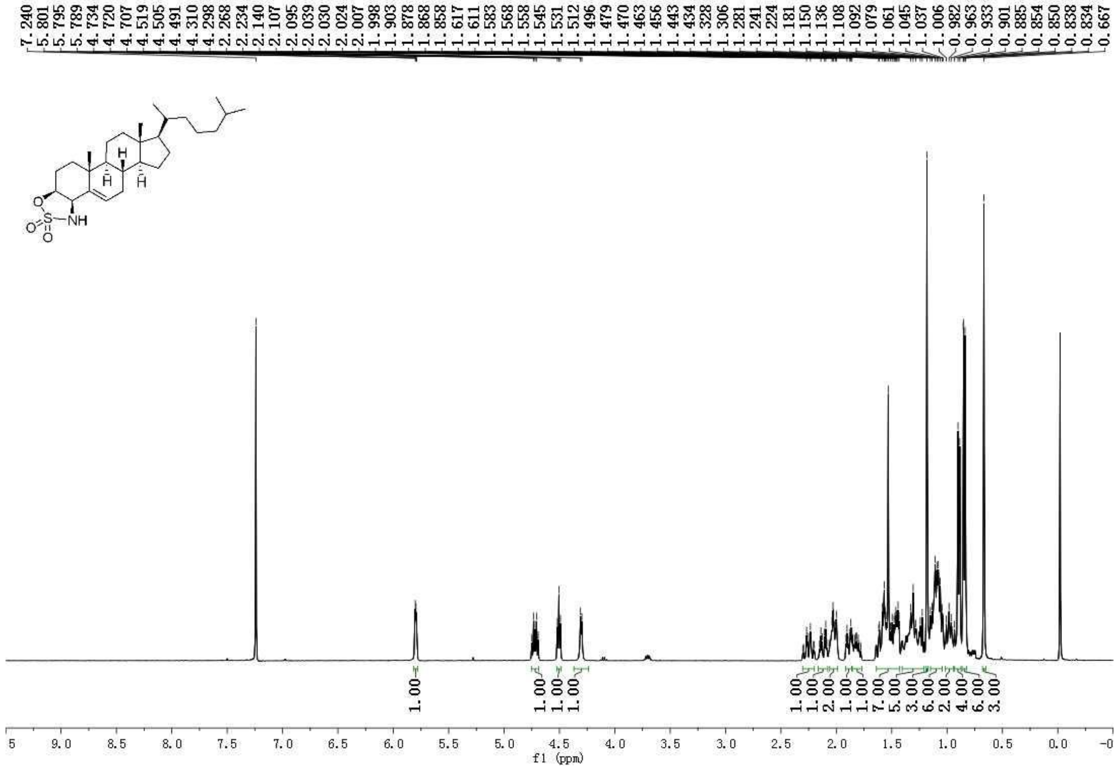

Figure S1. ^{1}H NMR spectrum of **6a** ($CDCl_3$, 400 MHz)

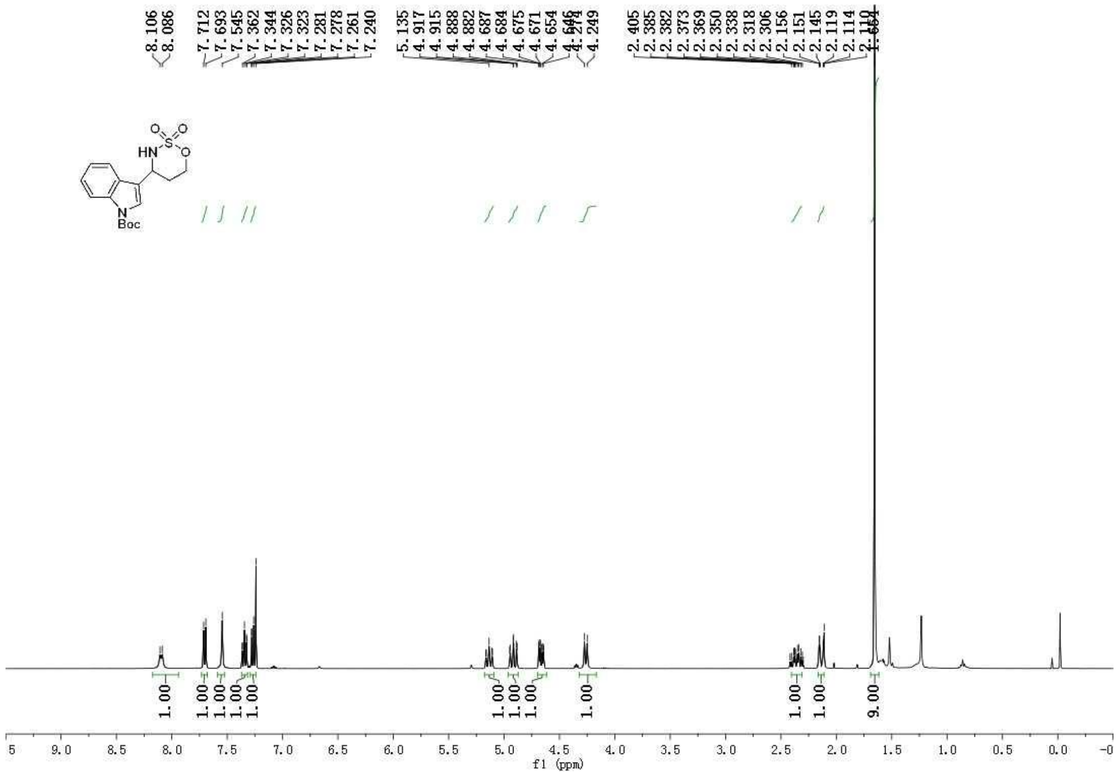

Figure S2. ^{1}H NMR spectrum of **6b** ($CDCl_3$, 400 MHz)

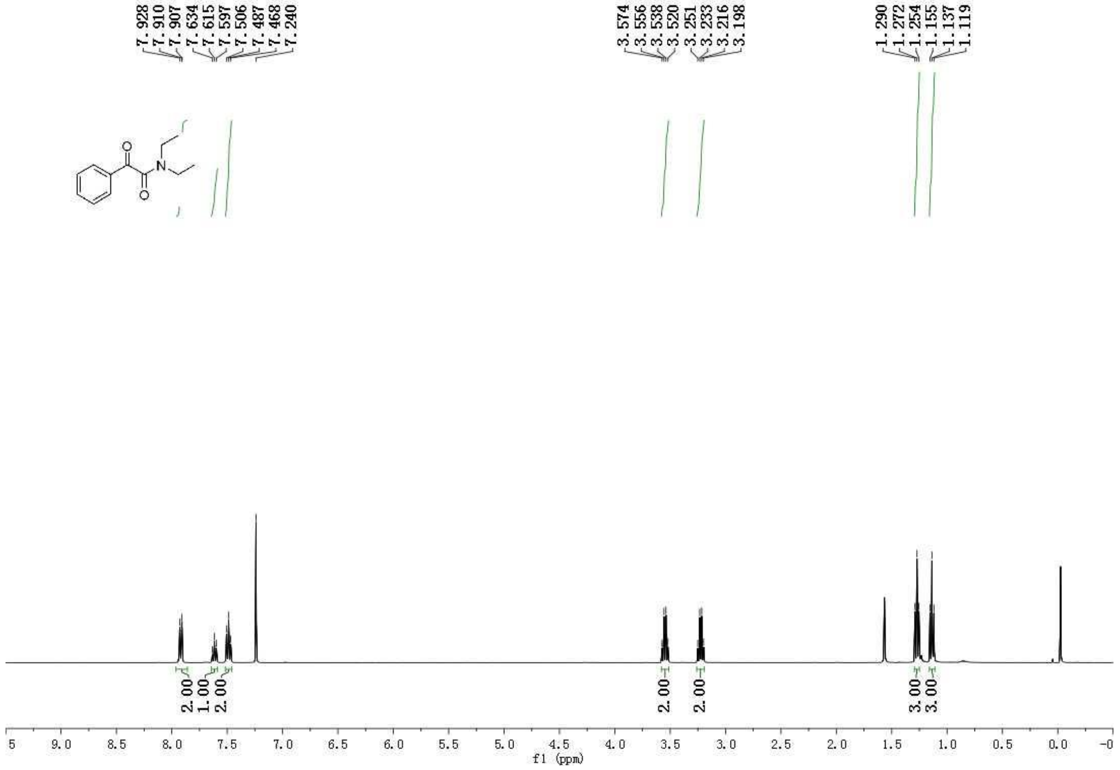

Figure S3. ^{1}H NMR spectrum of **S3** ($CDCl_3$, 400 MHz)

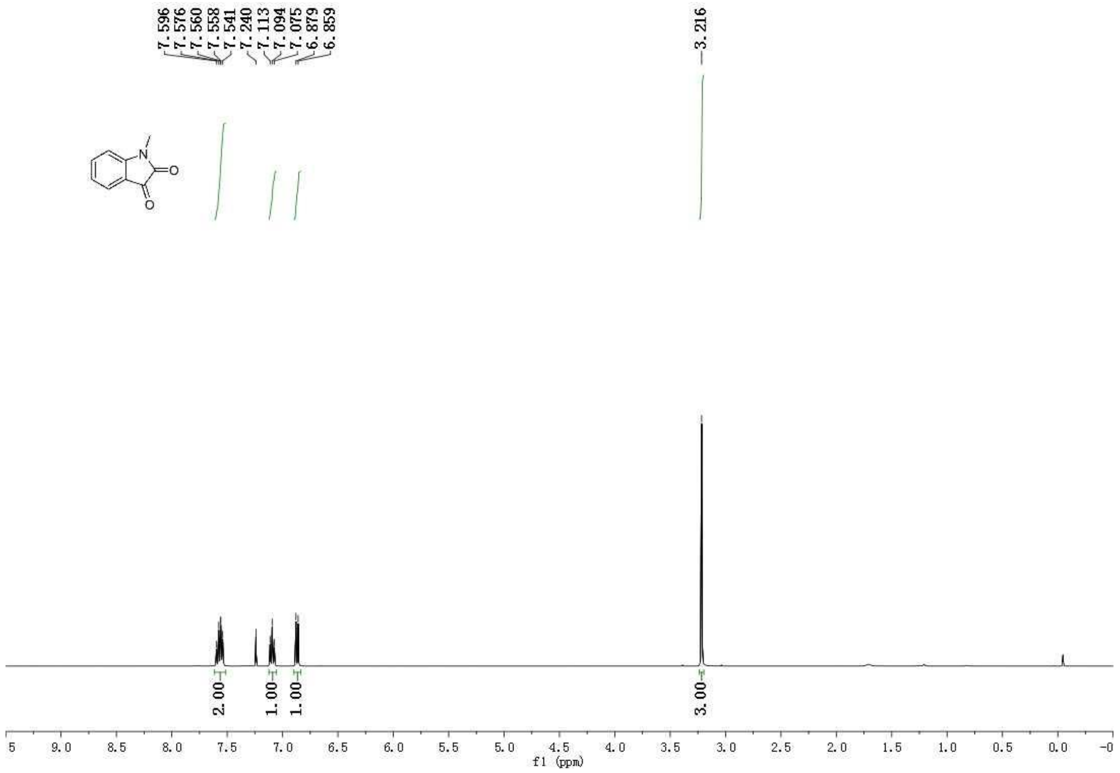

Figure S4. ^{1}H NMR spectrum of **S4** ($CDCl_3$, 400 MHz)

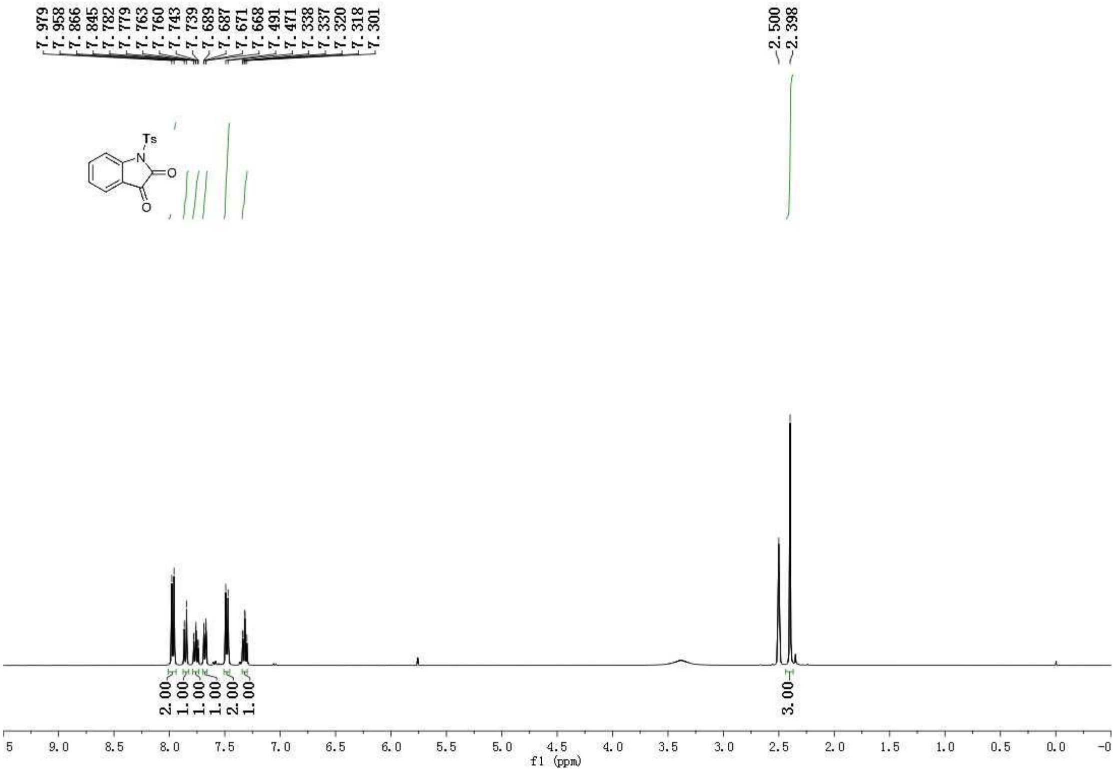

Figure S5. ^{1}H NMR spectrum of **S5** ($CDCl_3$, 400 MHz)

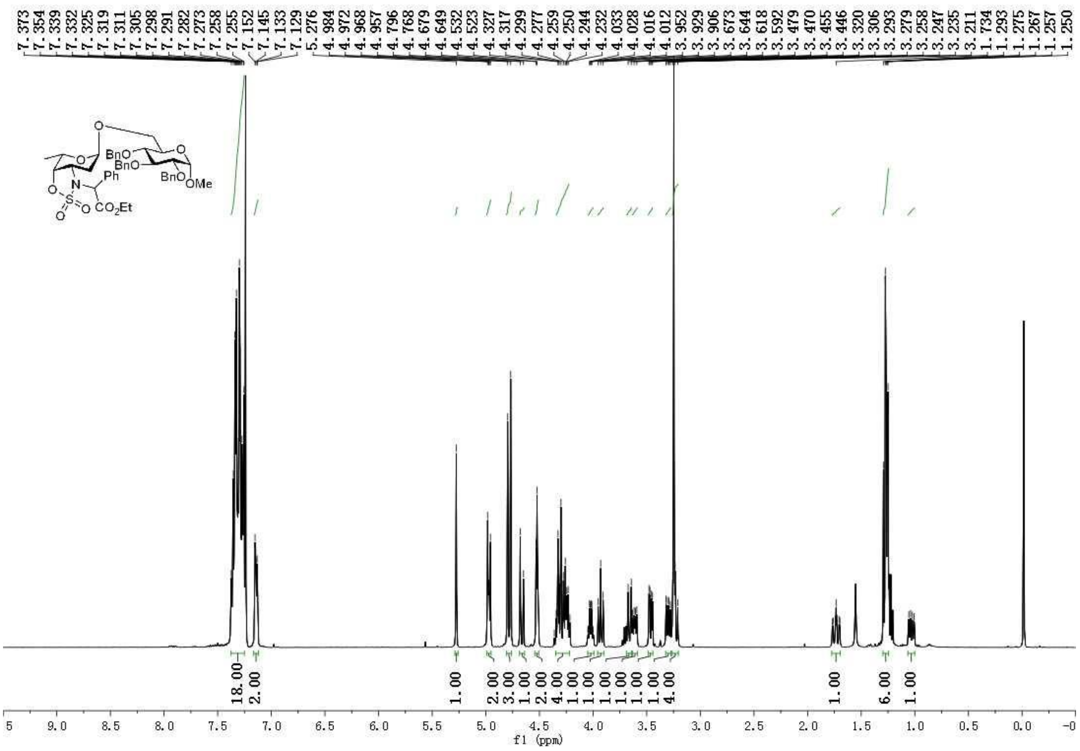

Figure S6. ^{1}H NMR spectrum of **3a** ($CDCl_3$, 400 MHz)

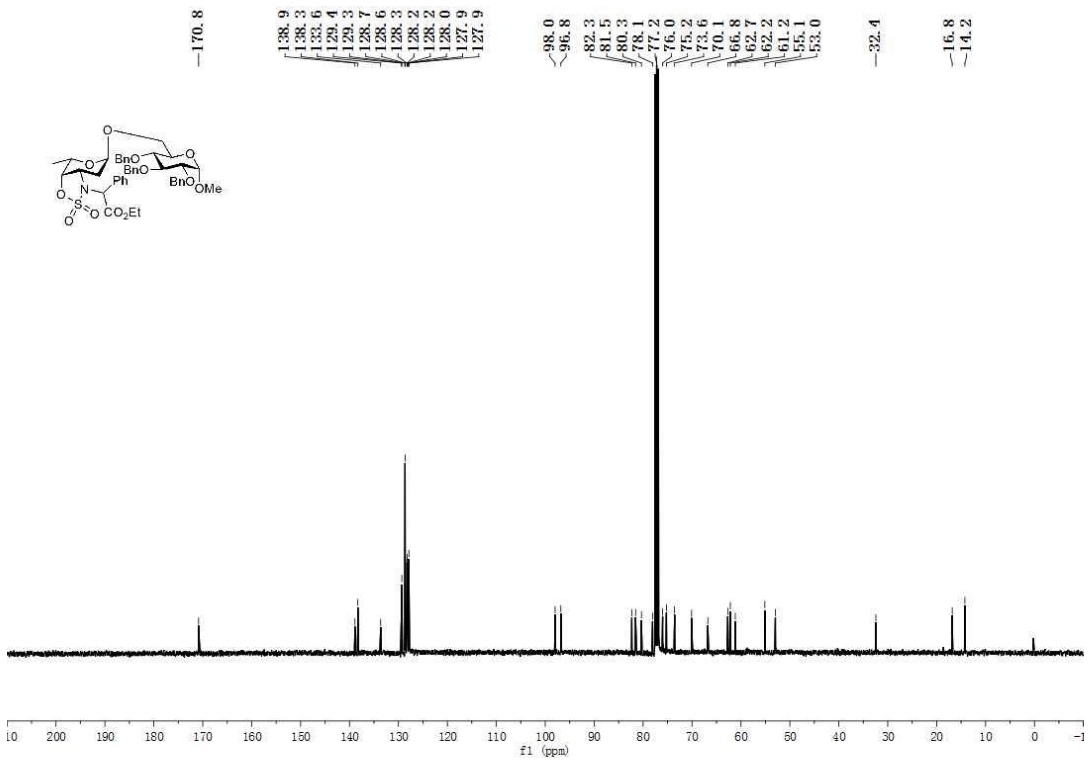

Figure S7. ^{13}C NMR spectrum of **3a** ($CDCl_3$, 100 MHz)

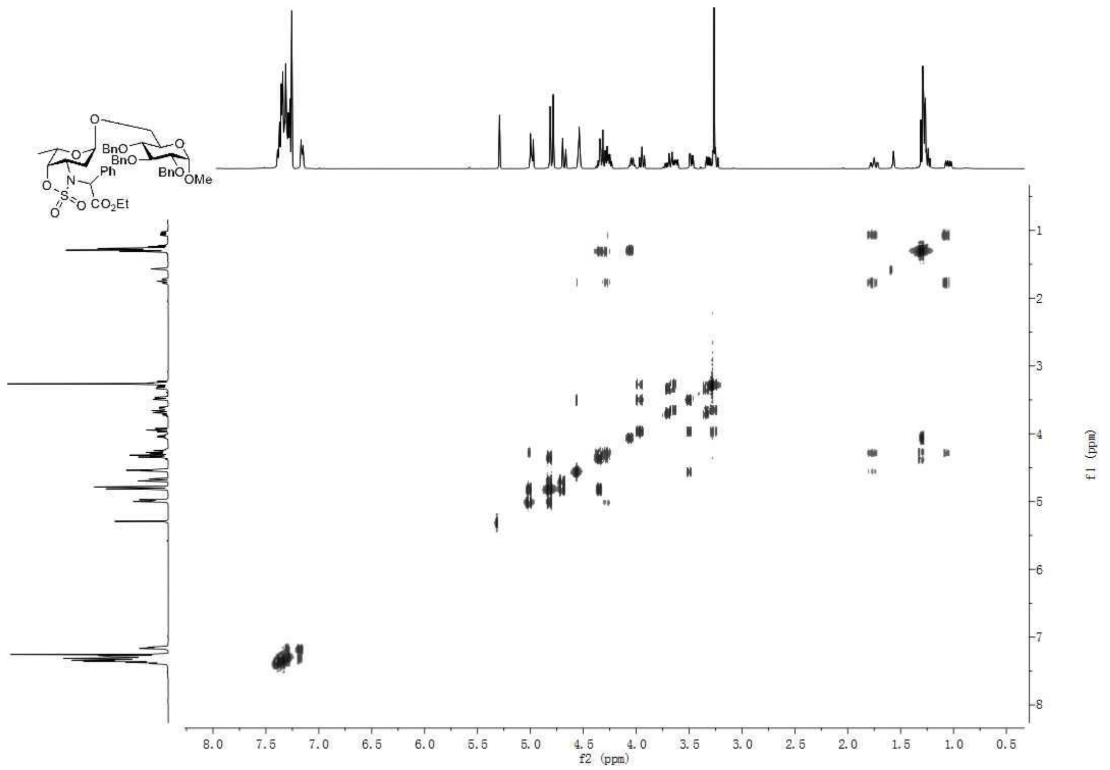

Figure S8. ^{1}H-^{1}H COSY spectrum of **3a** ($CDCl_3$, 400 MHz)

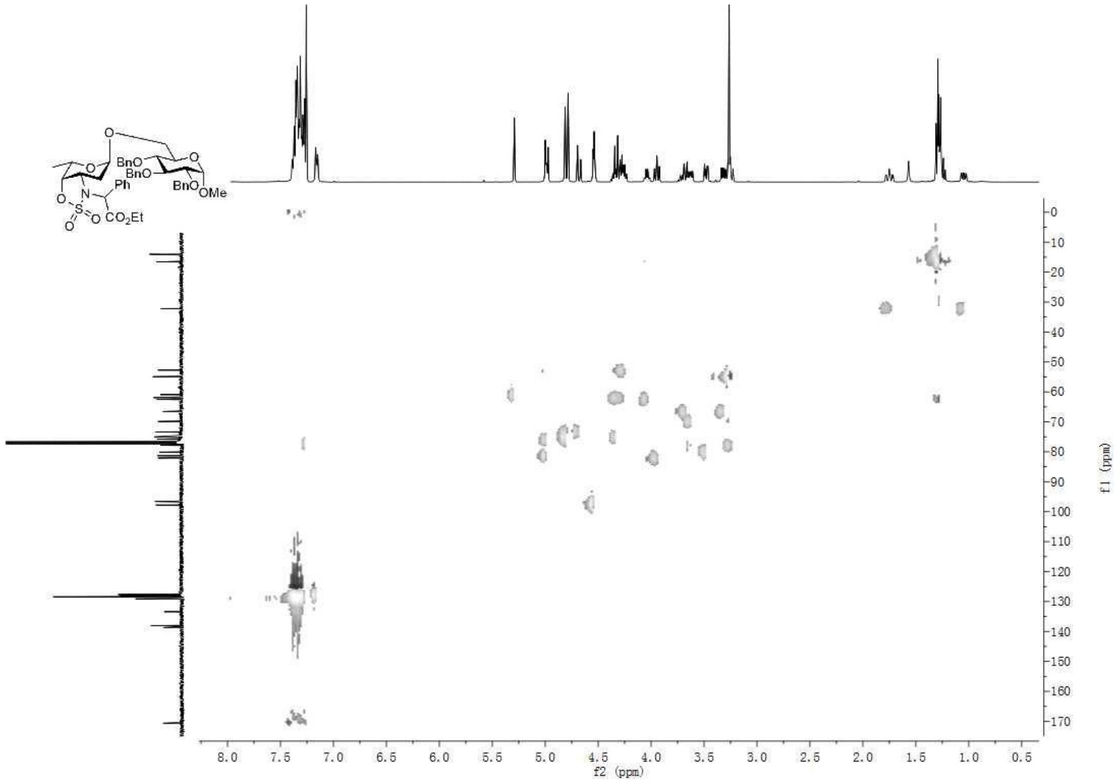

Figure S9. HSQC spectrum of **3a** ($CDCl_3$)

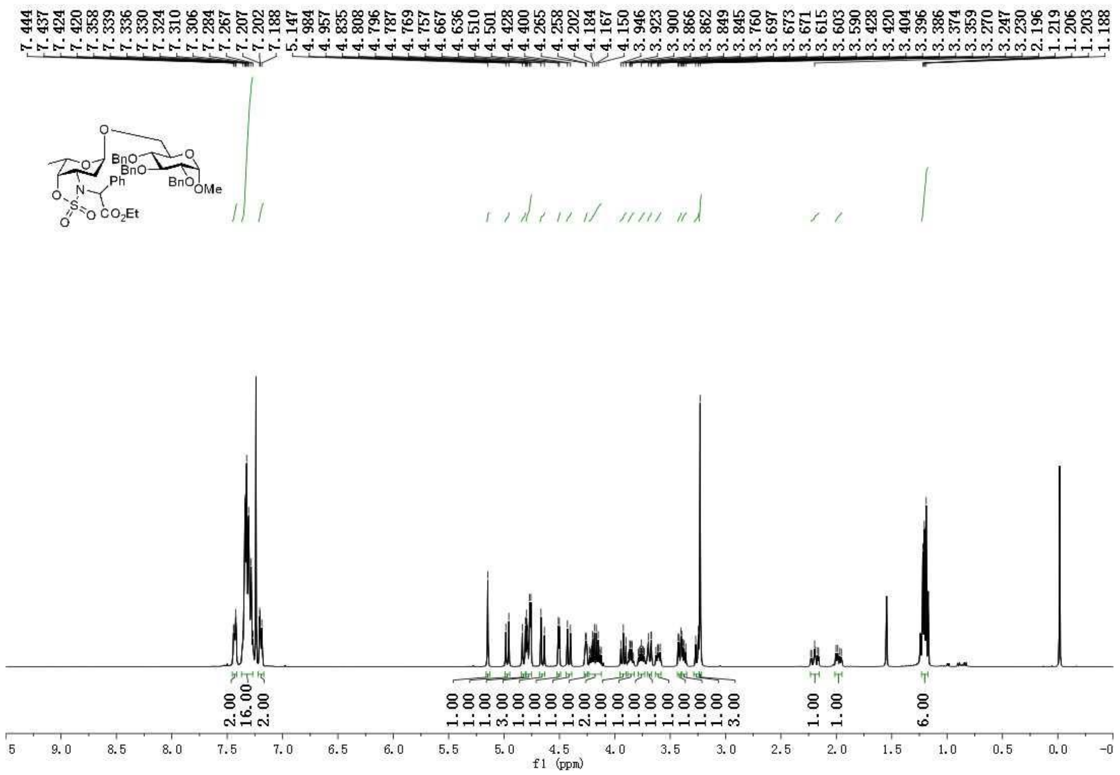

Figure S10. ^{1}H NMR spectrum of **3a′** ($CDCl_3$, 400 MHz)

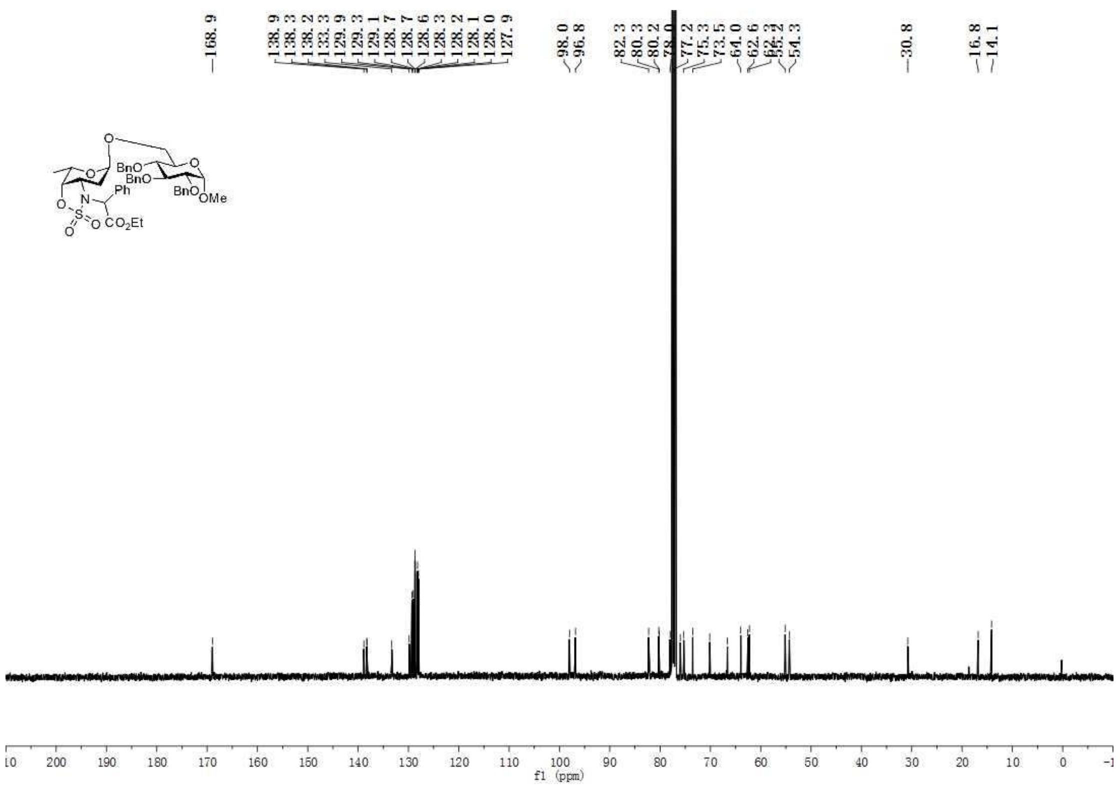

Figure S11. ^{13}C NMR spectrum of **3a′** ($CDCl_3$, 100 MHz)

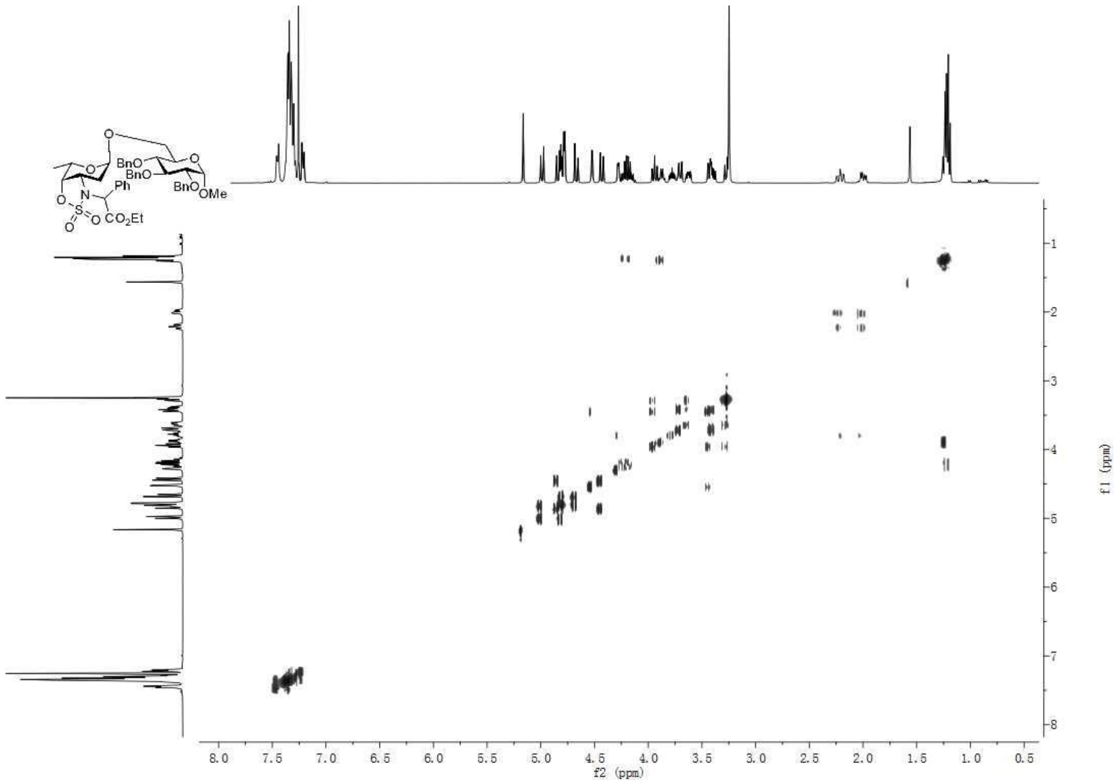

Figure S12. ^{1}H-^{1}H COSY spectrum of **3a′** ($CDCl_3$, 400 MHz)

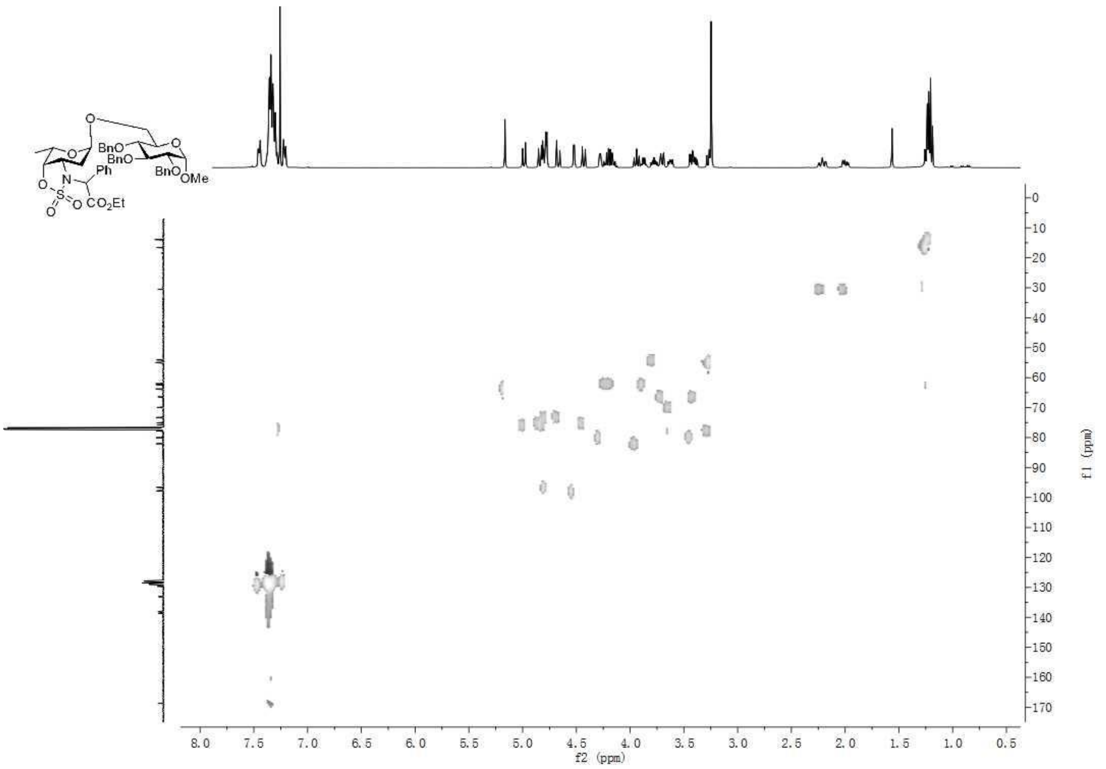

Figure S13. HSQC spectrum of **3a′** ($CDCl_3$)

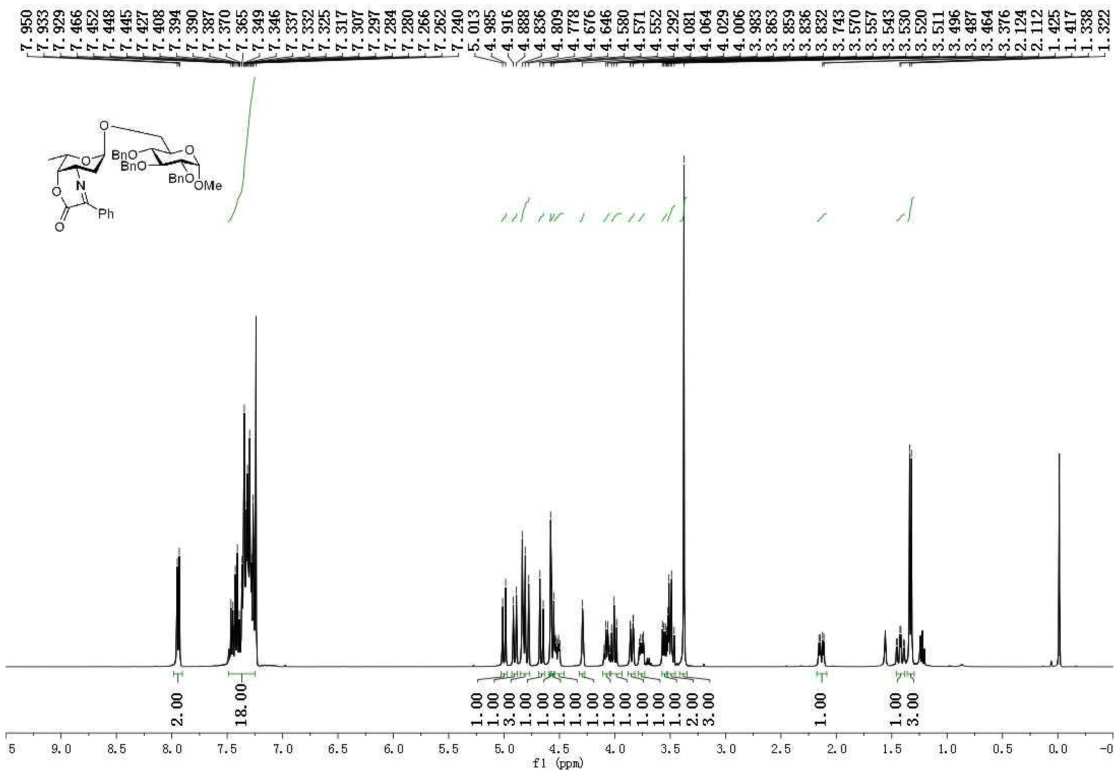

Figure S14. ^{1}H NMR spectrum of **4a** ($CDCl_3$, 400 MHz)

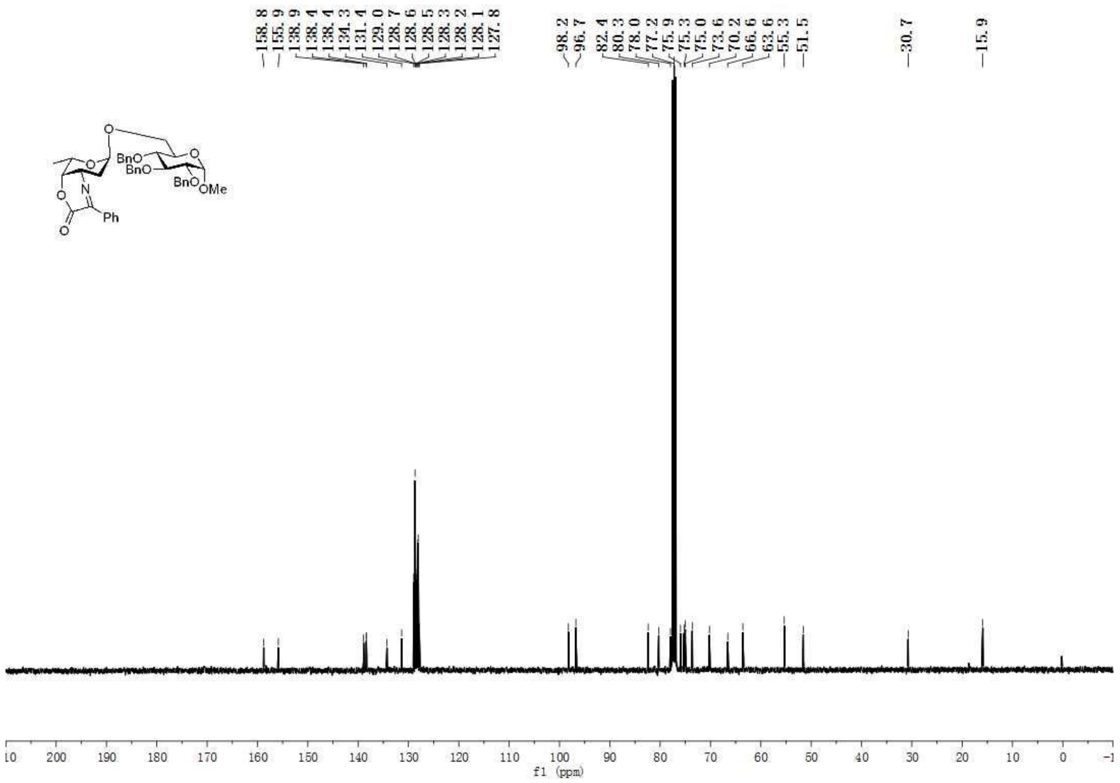

Figure S15. ^{13}C NMR spectrum of **4a** ($CDCl_3$, 100 MHz)

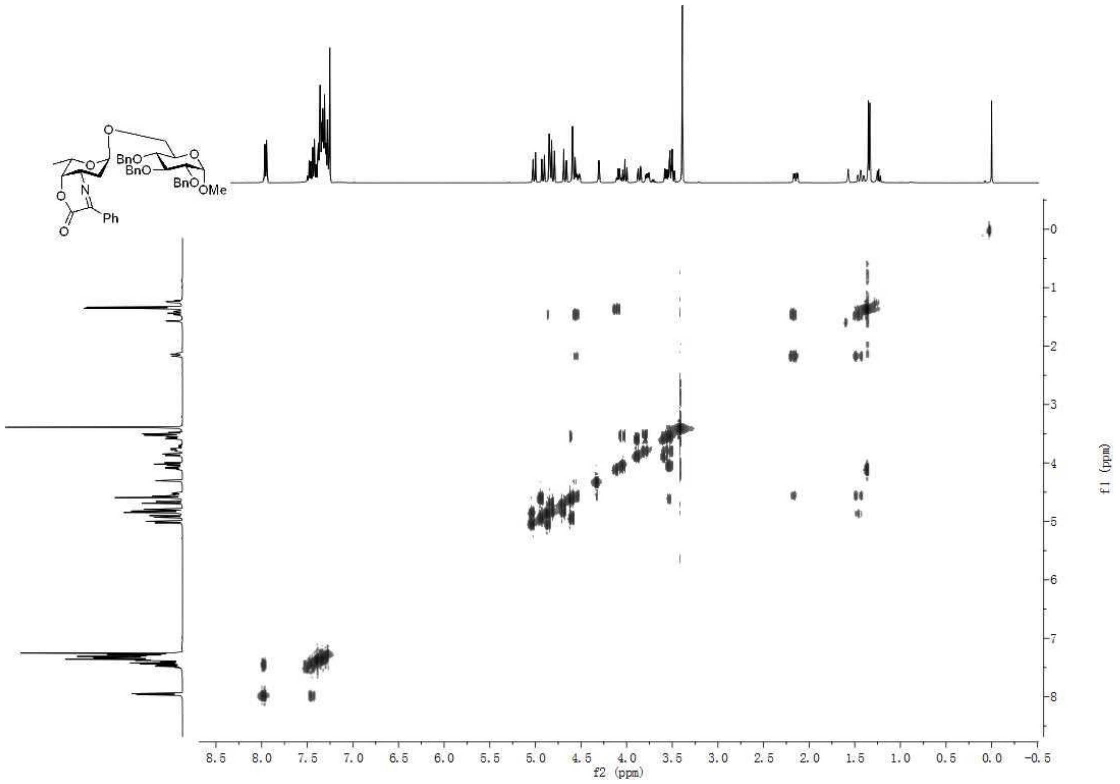

Figure S16. ^{1}H-^{1}H COSY spectrum of **4a** ($CDCl_3$, 400 MHz)

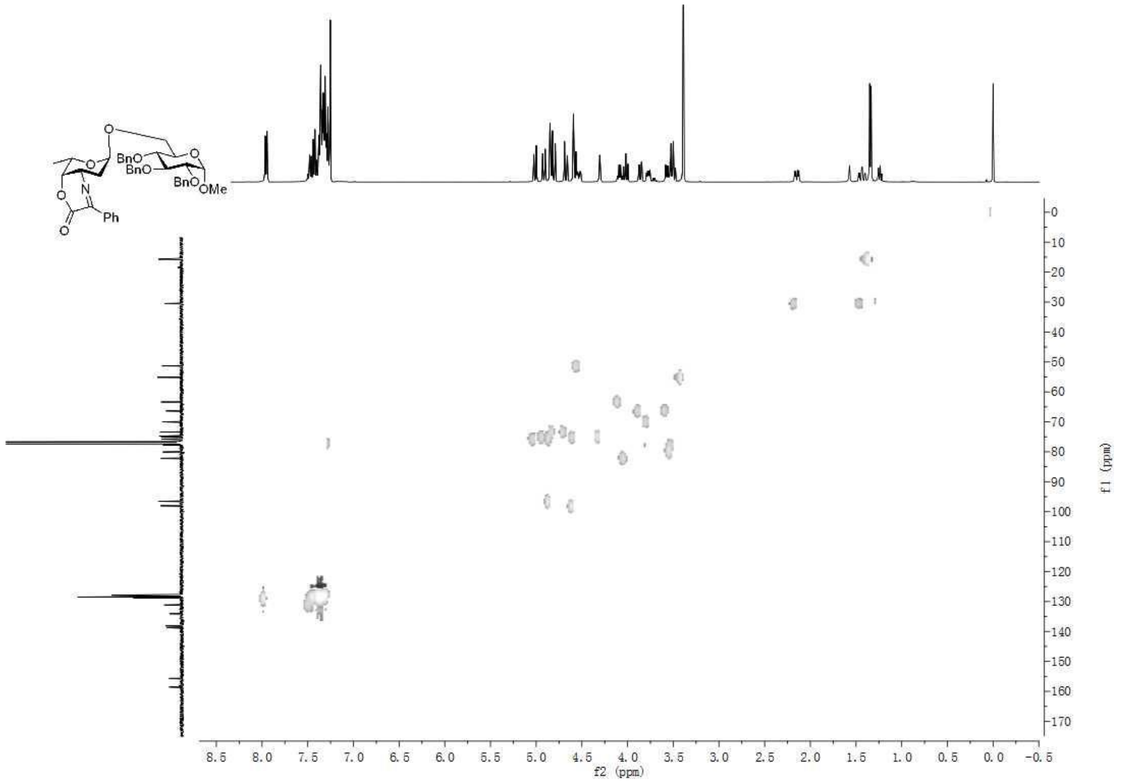

Figure S17. HSQC spectrum of **4a** ($CDCl_3$)

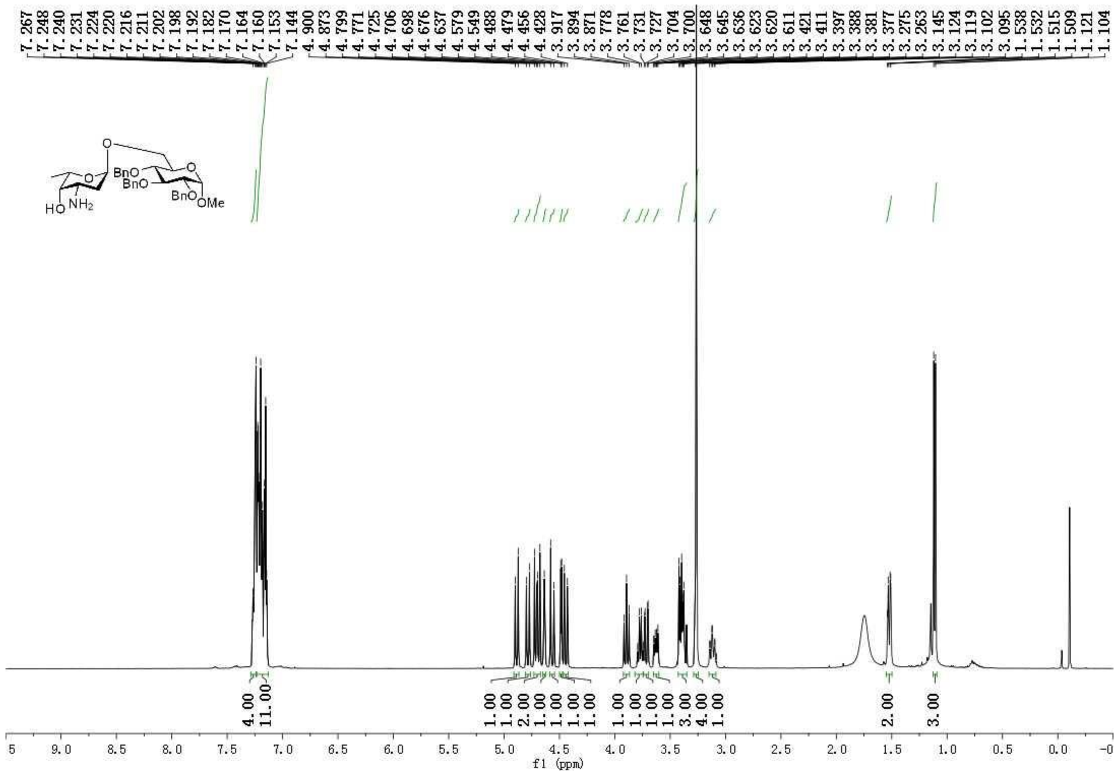

Figure S18. ^{1}H NMR spectrum of **2a** ($CDCl_3$, 400 MHz)

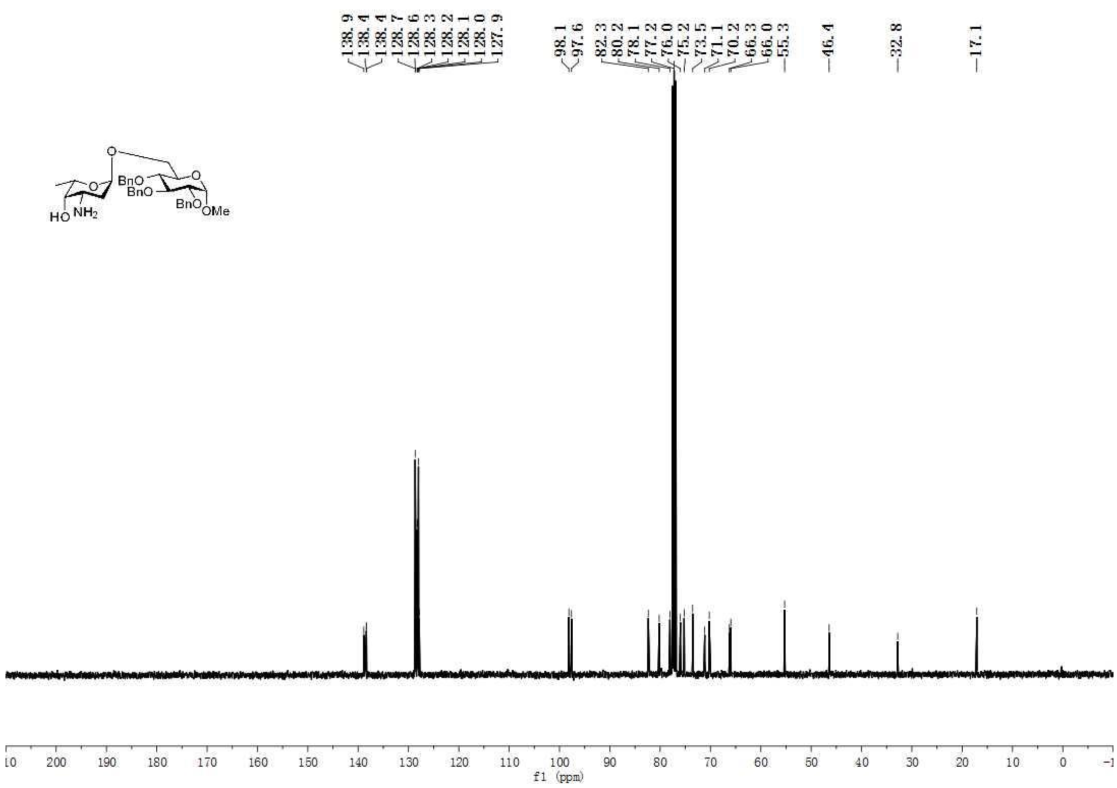

Figure S19. ^{13}C NMR spectrum of **2a** ($CDCl_3$, 100 MHz)

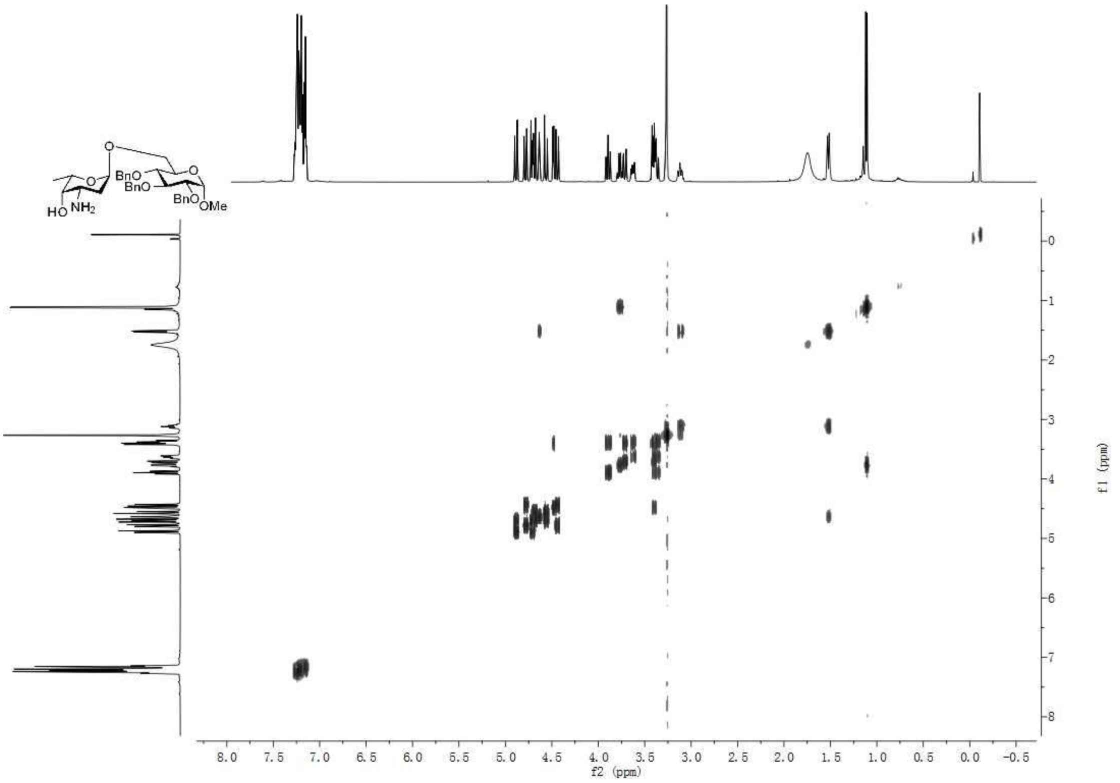

Figure S20. ^{1}H-^{1}H COSY spectrum of **2a** ($CDCl_3$, 400 MHz)

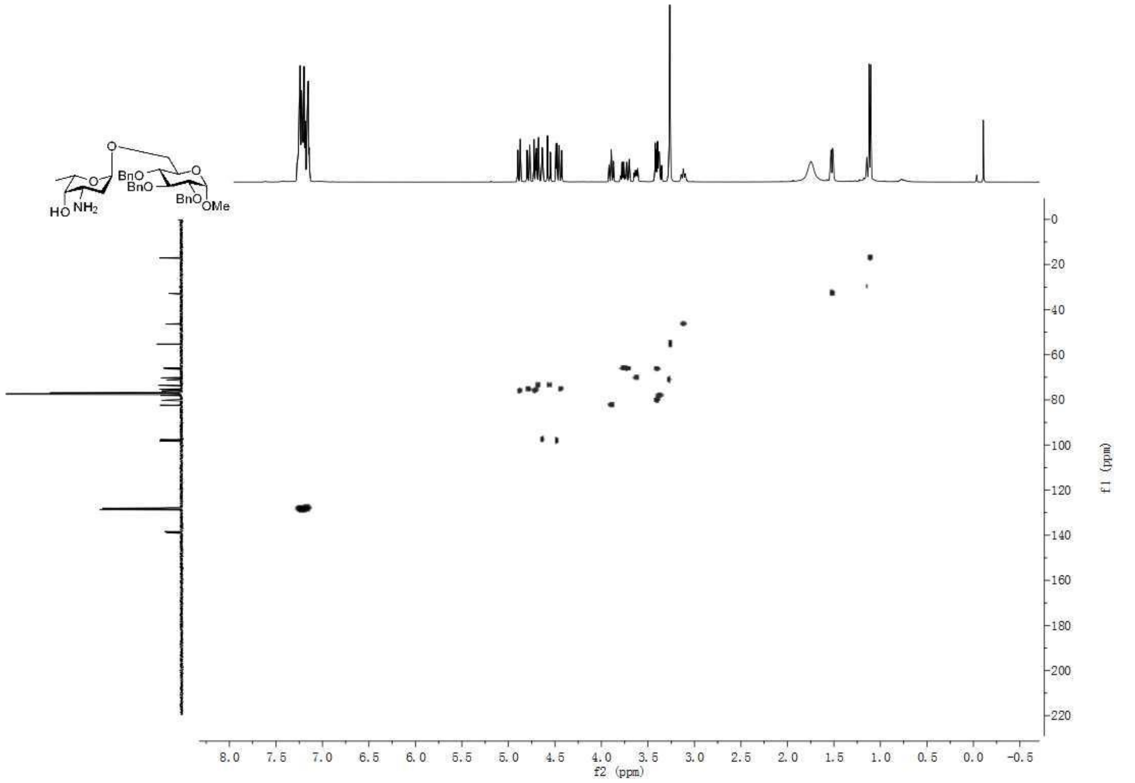

Figure S21. HSQC spectrum of **2a** ($CDCl_3$)

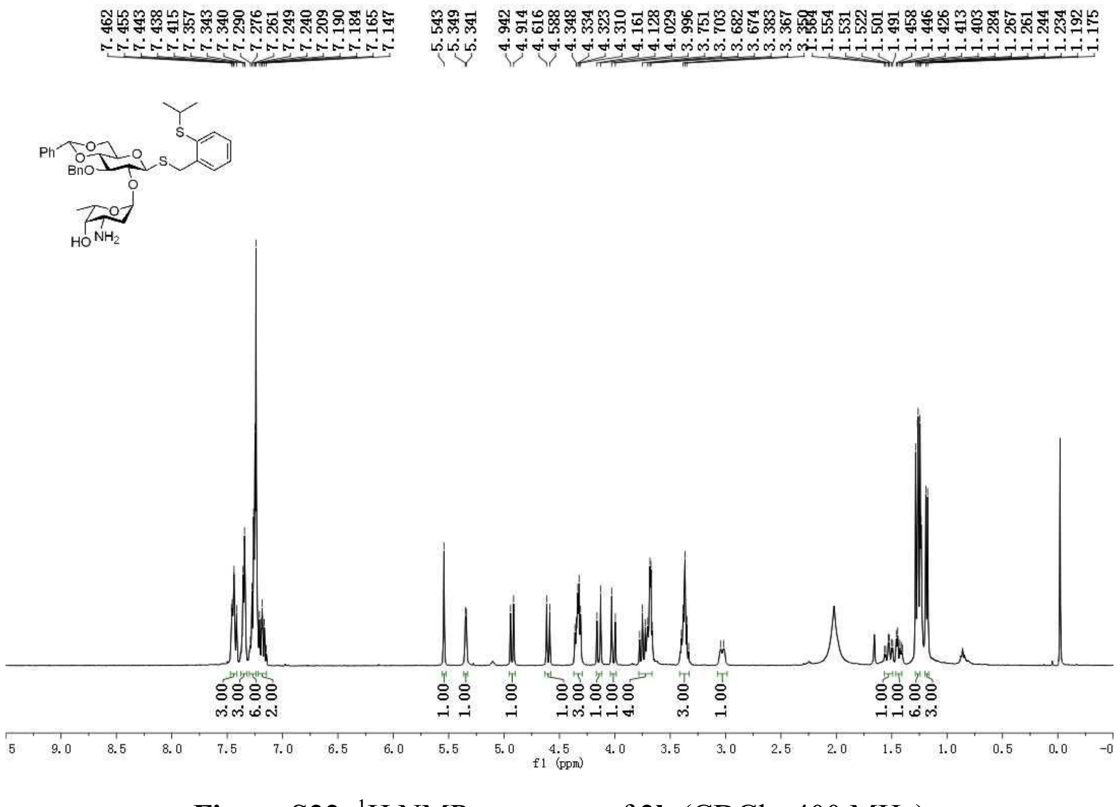

Figure S22. ^{1}H NMR spectrum of **2b** ($CDCl_3$, 400 MHz)

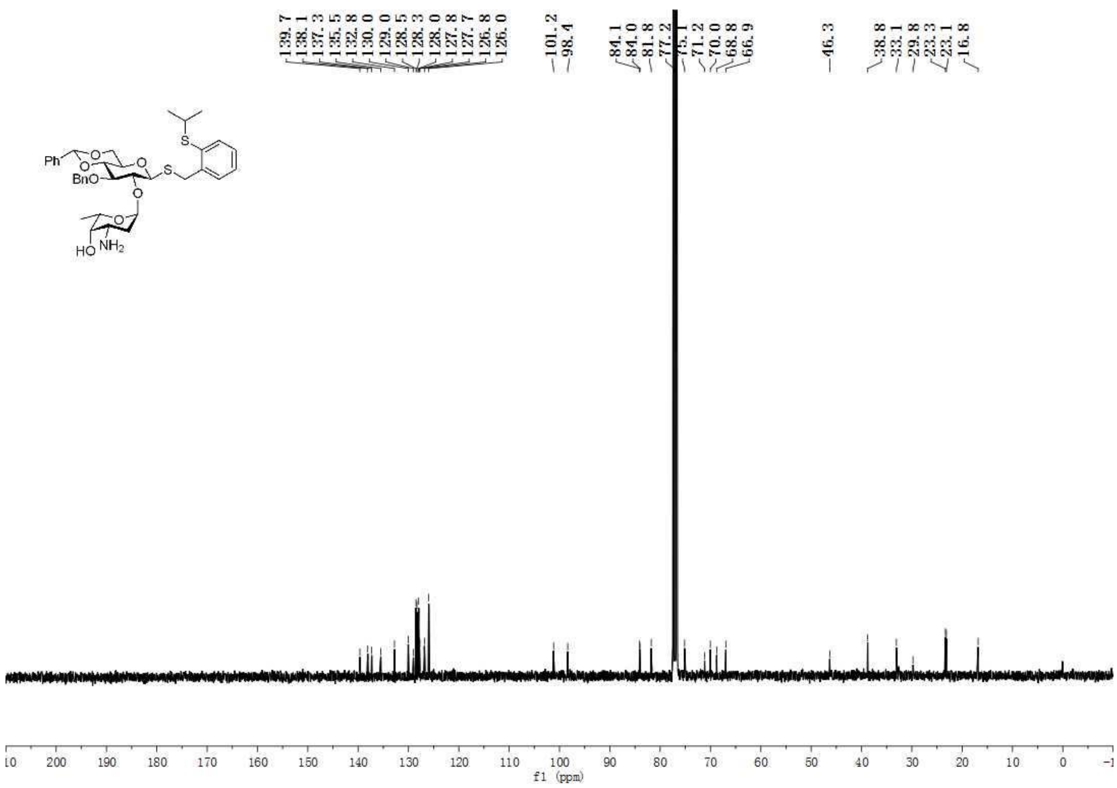

Figure S23. ^{13}C NMR spectrum of **2b** ($CDCl_3$, 100 MHz)

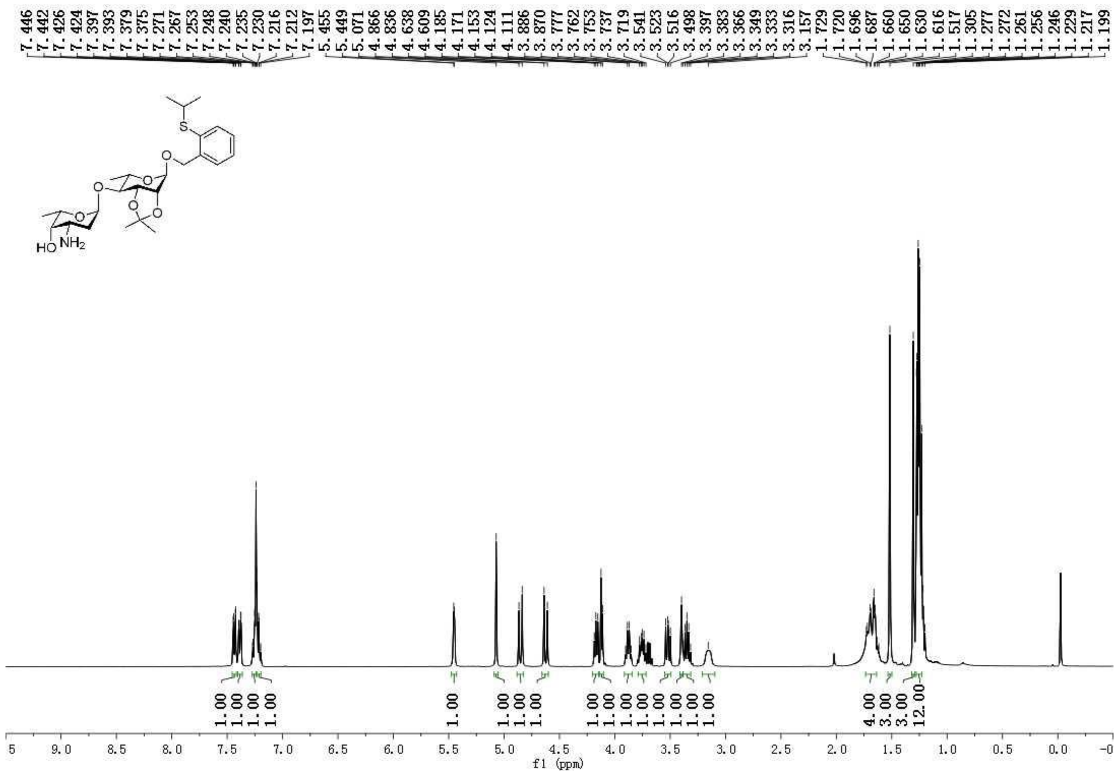

Figure S24. ^{1}H NMR spectrum of **2c** ($CDCl_3$, 400 MHz)

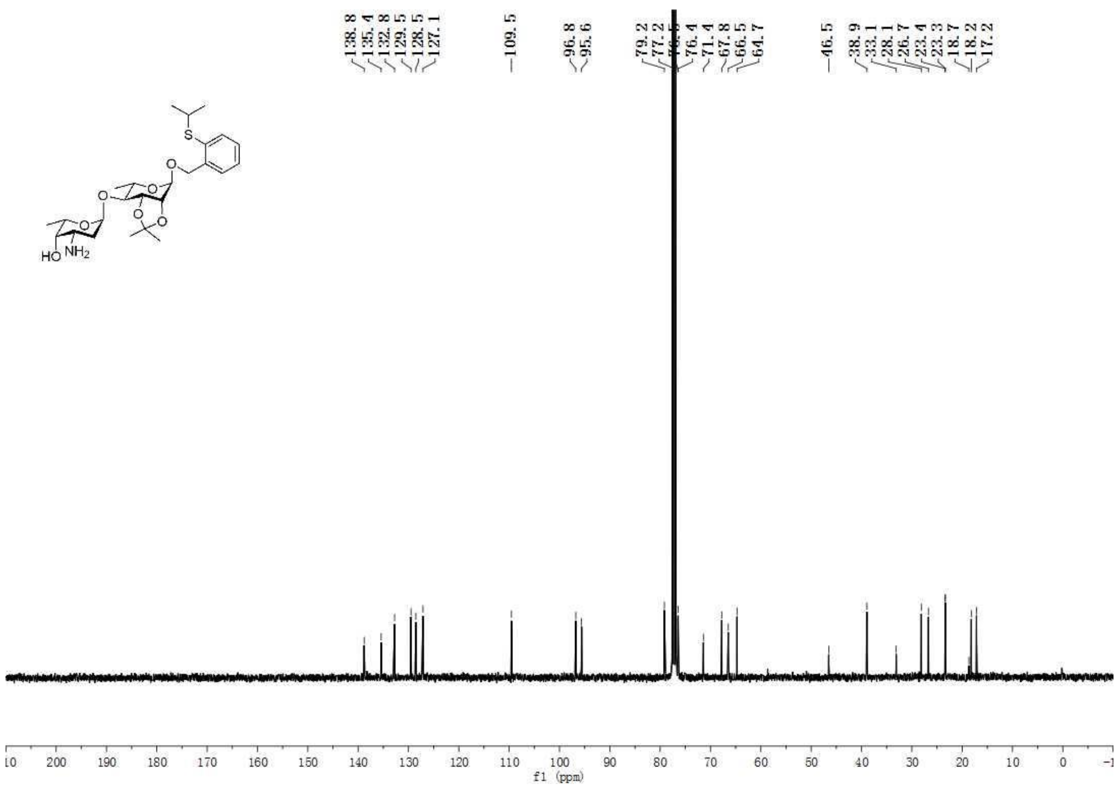

Figure S25. ^{13}C NMR spectrum of **2c** ($CDCl_3$, 100 MHz)

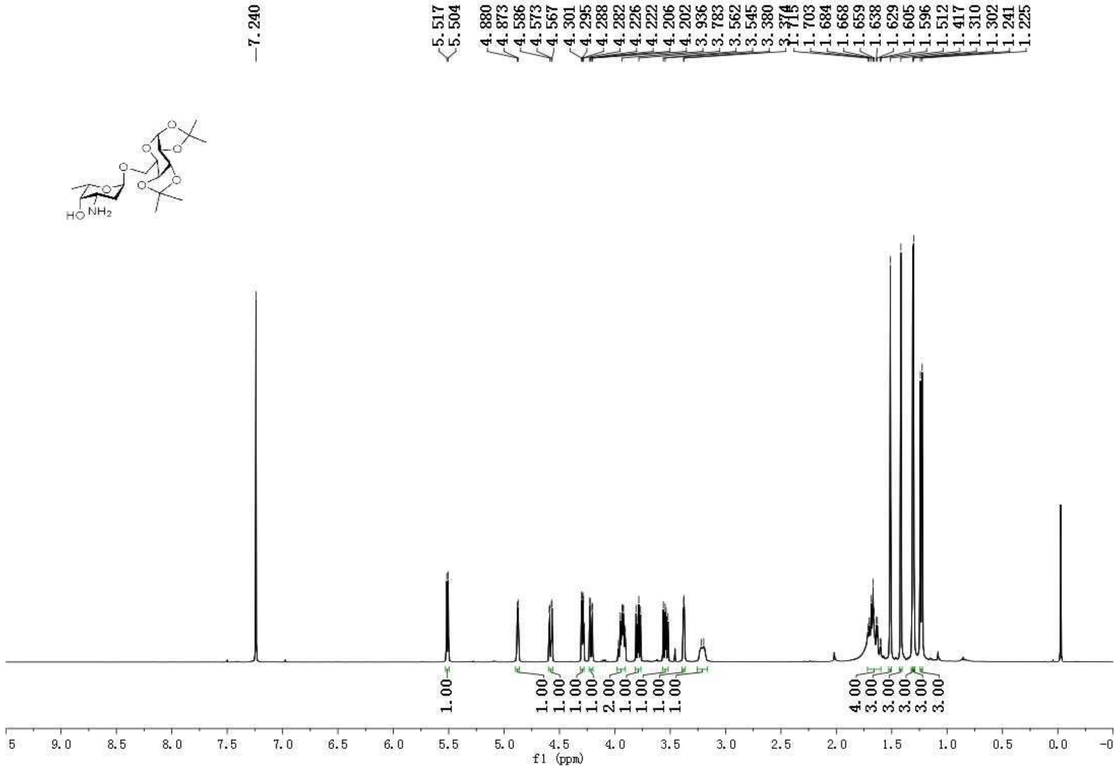

Figure S26. ^{1}H NMR spectrum of **2d** ($CDCl_3$, 400 MHz)

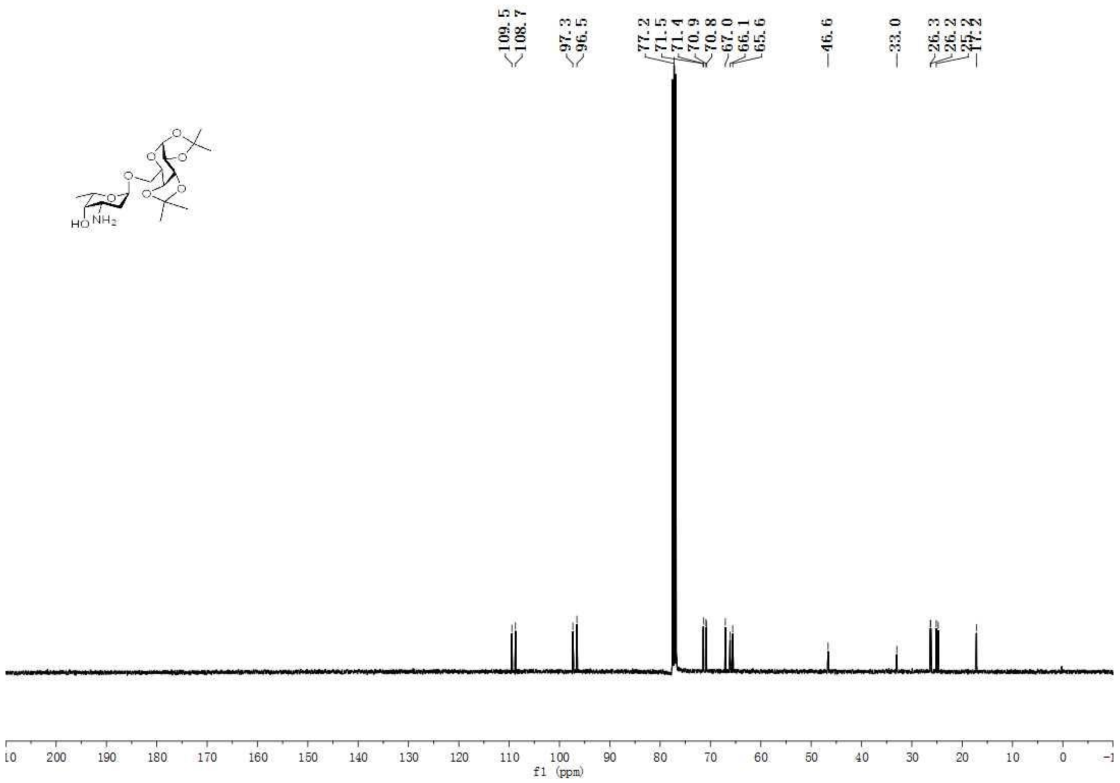

Figure S27. ^{13}C NMR spectrum of **2d** ($CDCl_3$, 100 MHz)

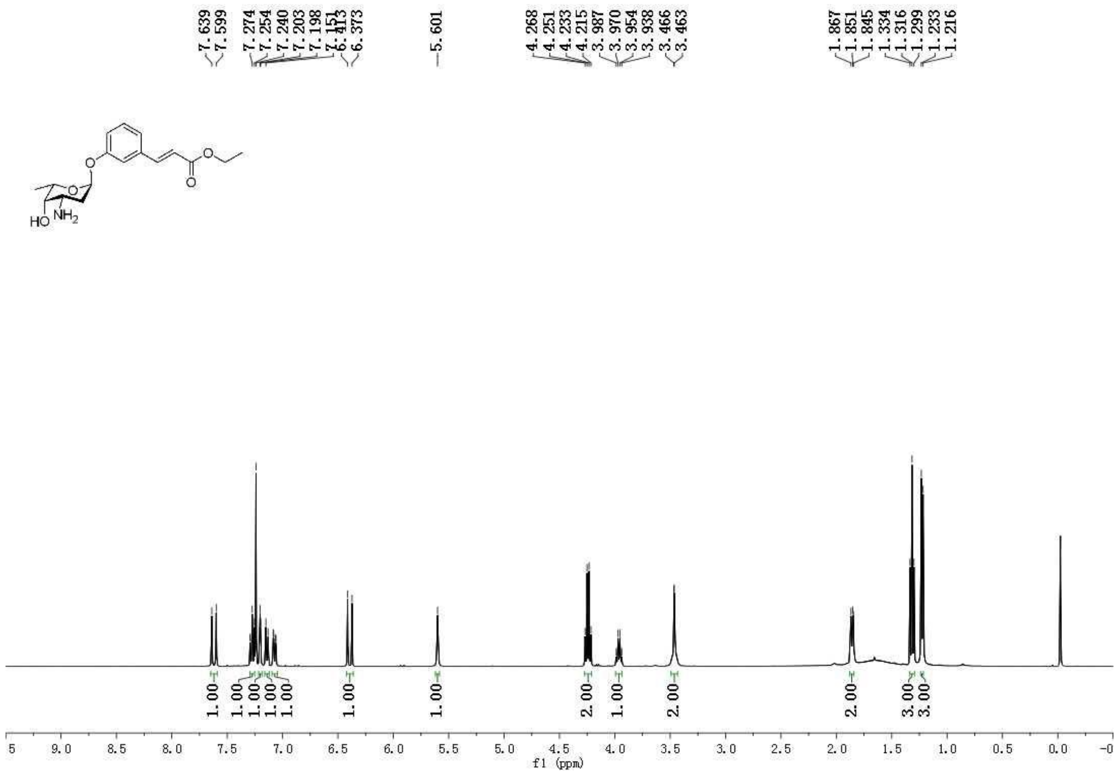

Figure S28. ^{1}H NMR spectrum of **2e** ($CDCl_3$, 400 MHz)

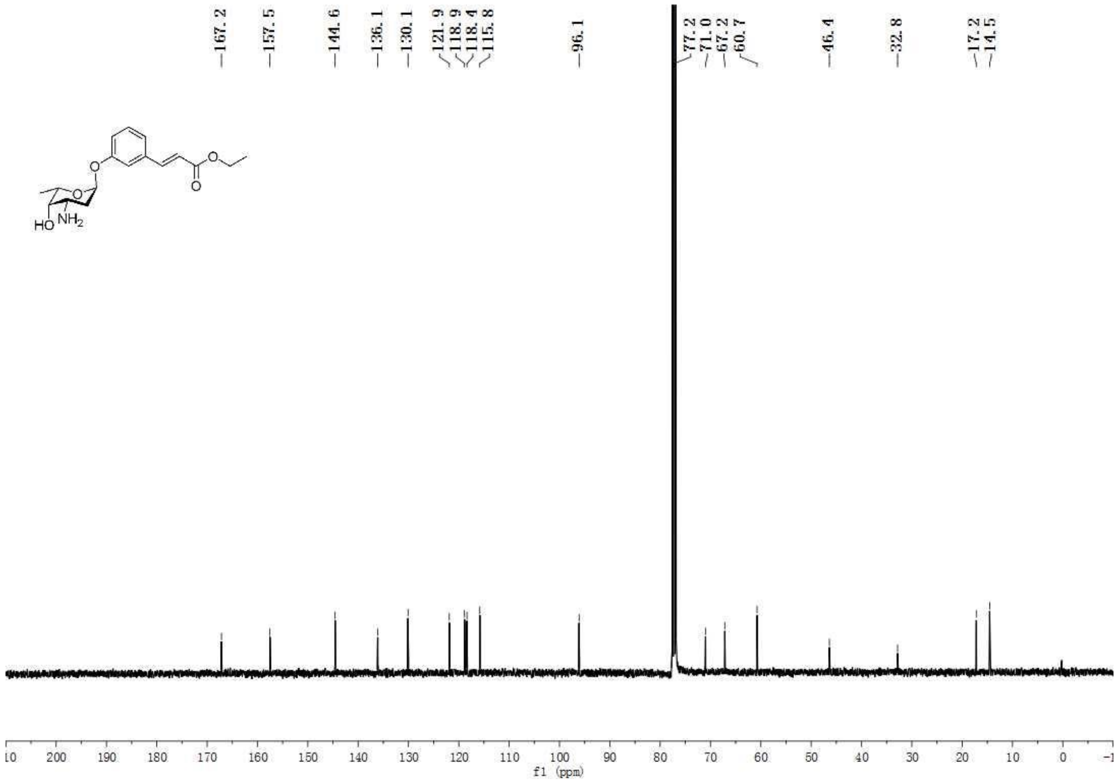

Figure S29. ^{13}C NMR spectrum of **2e** ($CDCl_3$, 100 MHz)

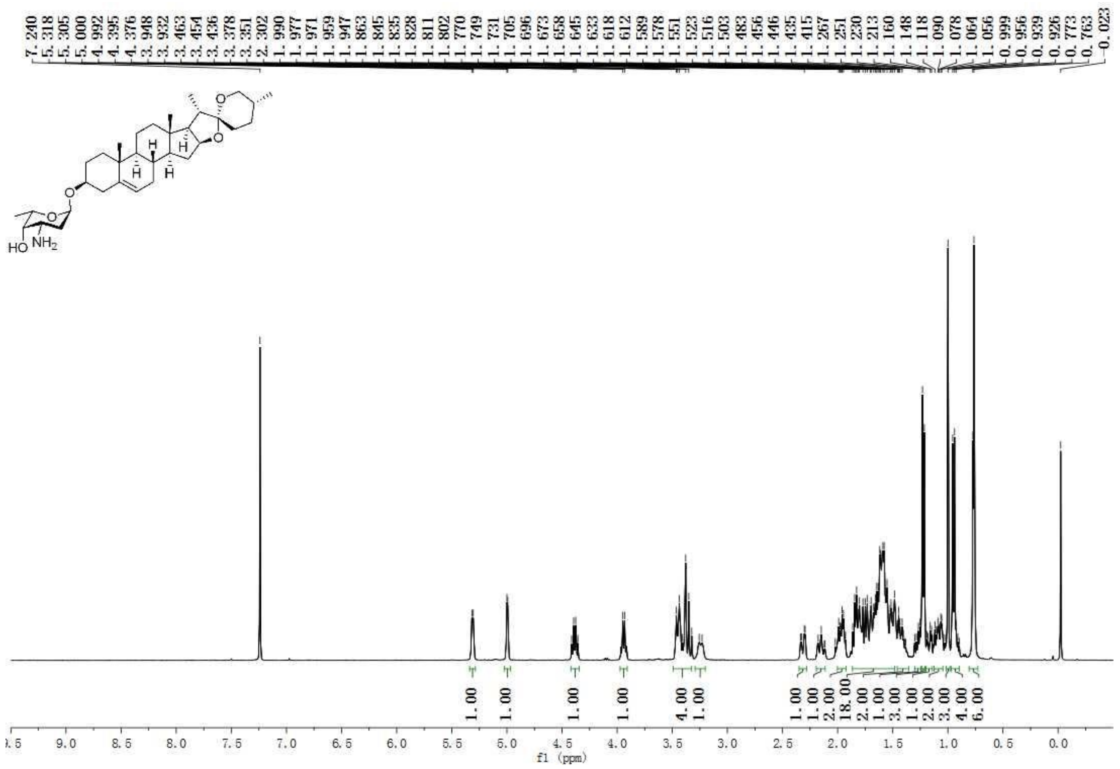

Figure S30. ^{1}H NMR spectrum of **2f** ($CDCl_3$, 400 MHz)

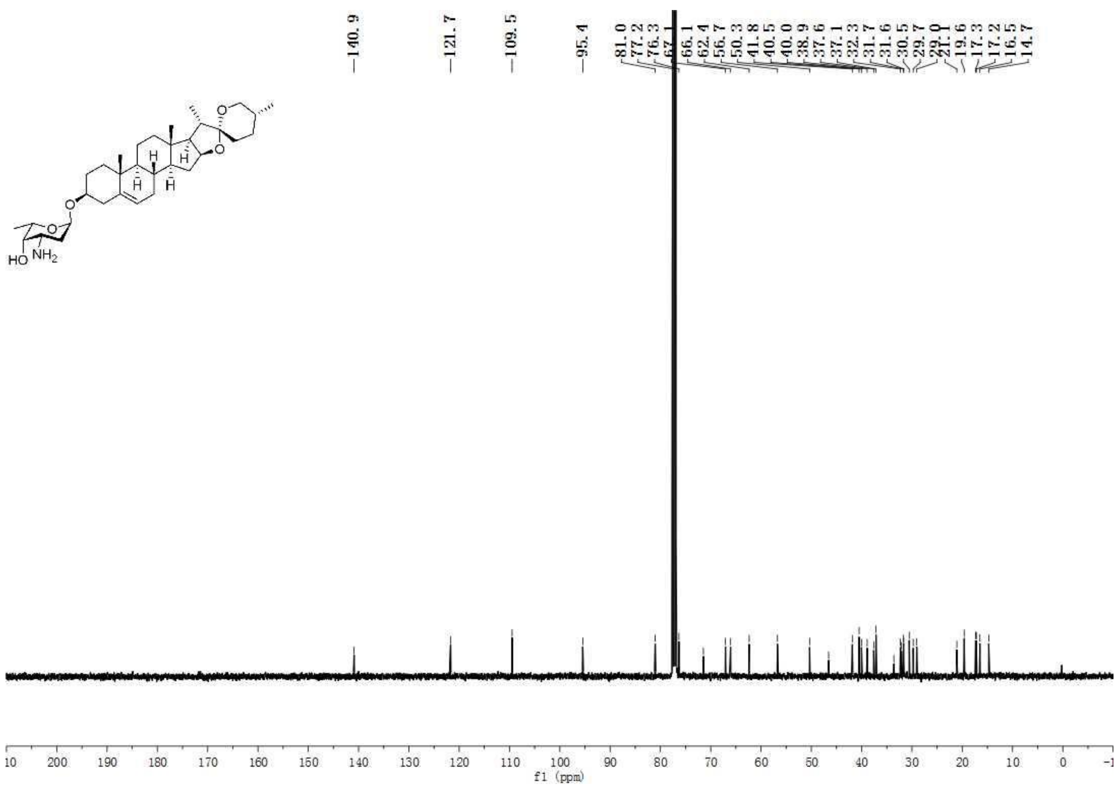

Figure S31. ^{13}C NMR spectrum of **2f** ($CDCl_3$, 100 MHz)

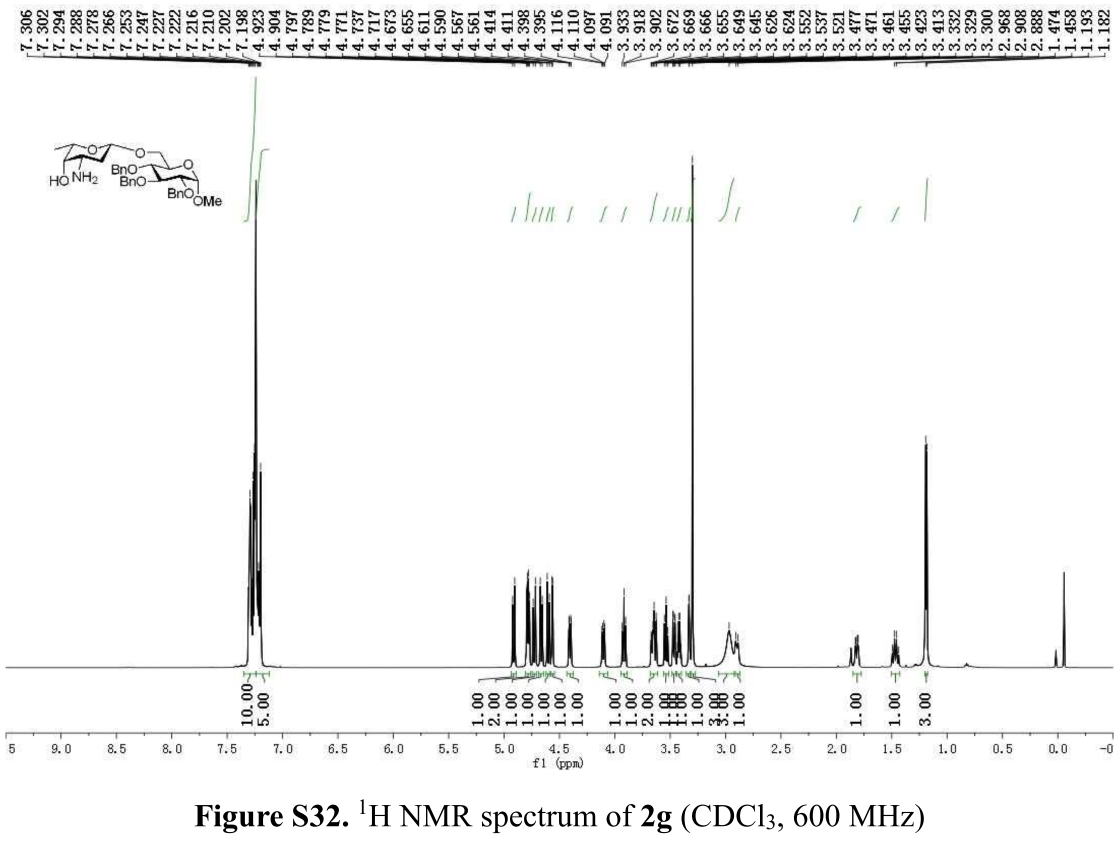

Figure S32. ^{1}H NMR spectrum of **2g** ($CDCl_3$, 600 MHz)

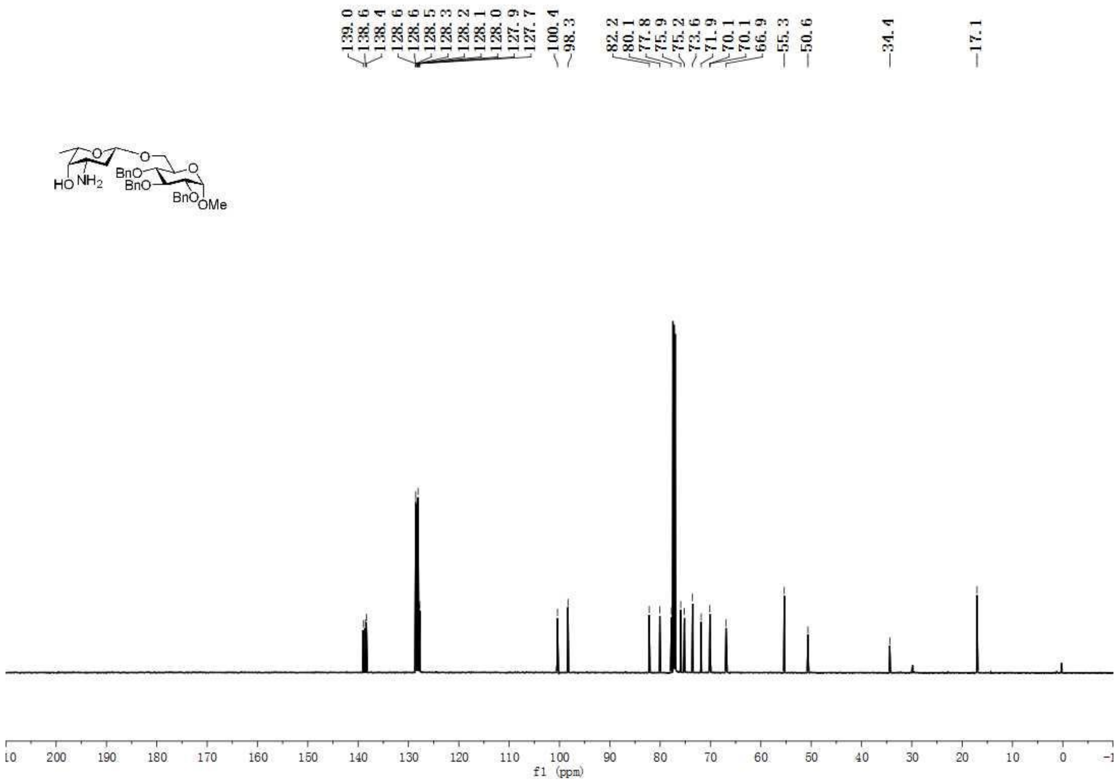

Figure S33. ^{13}C NMR spectrum of **2g** ($CDCl_3$, 125 MHz)

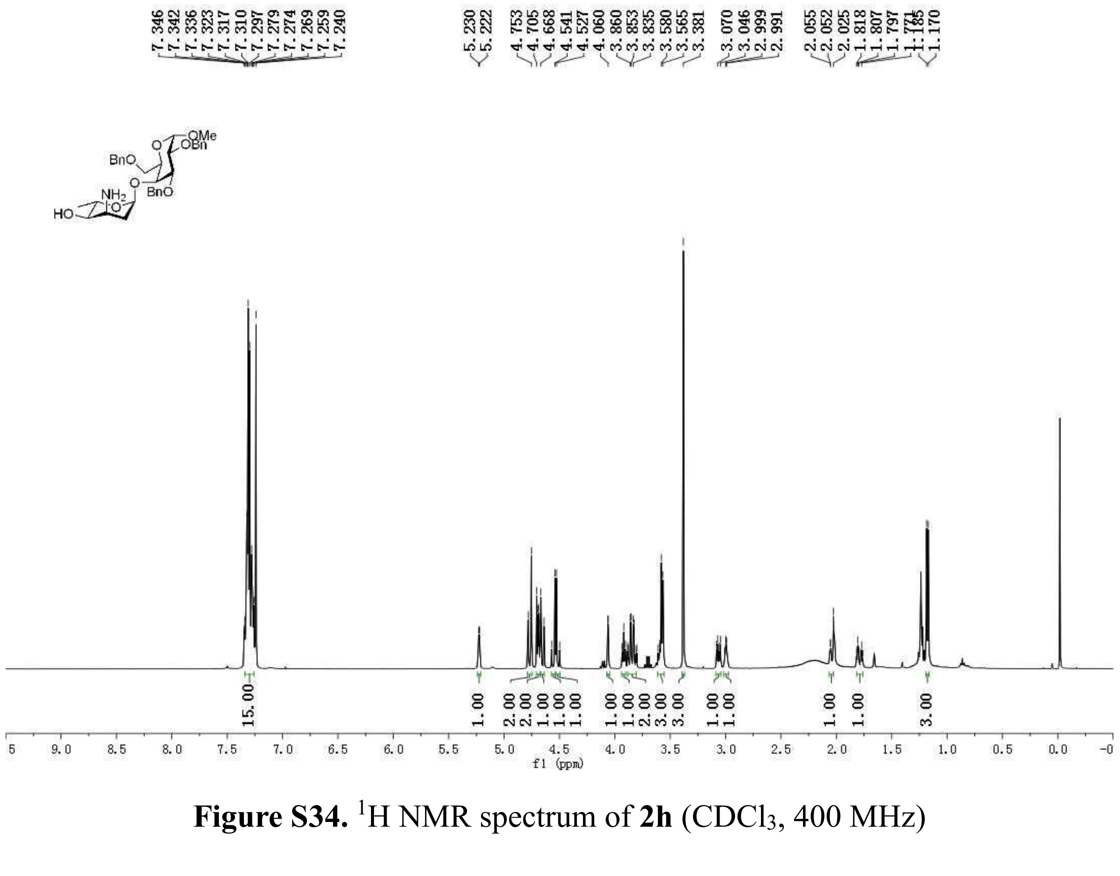

Figure S34. ^{1}H NMR spectrum of **2h** ($CDCl_3$, 400 MHz)

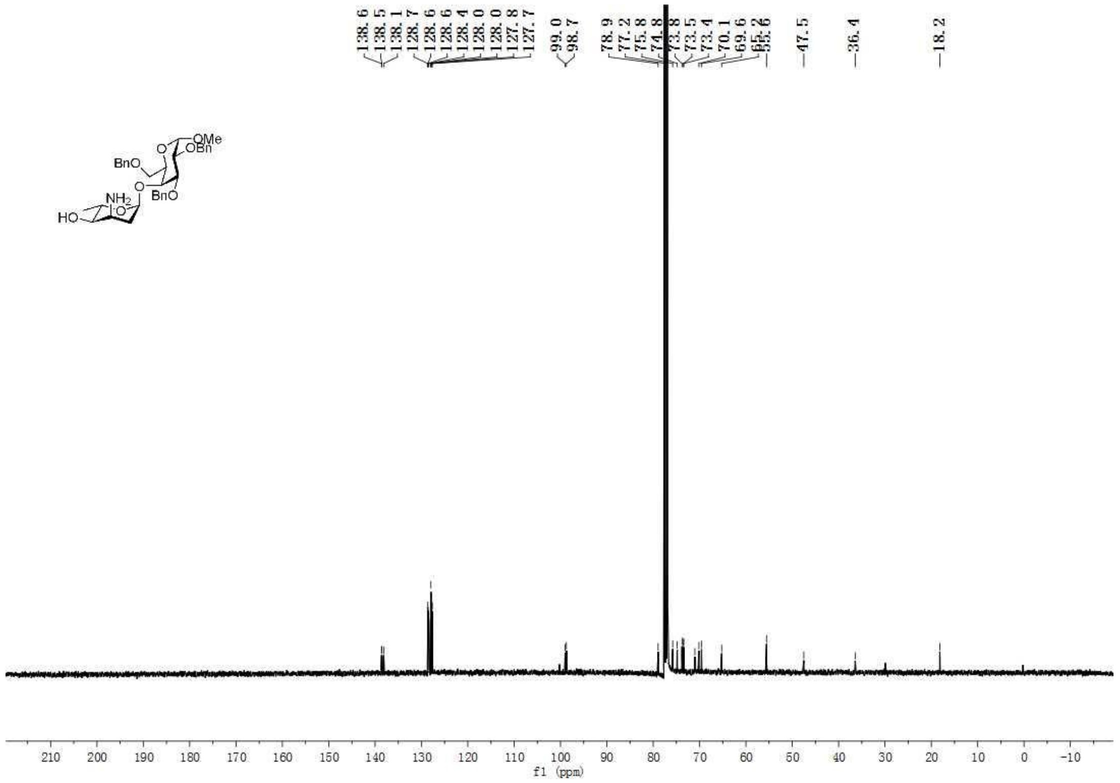

Figure S35. ^{13}C NMR spectrum of **2h** ($CDCl_3$, 100 MHz)

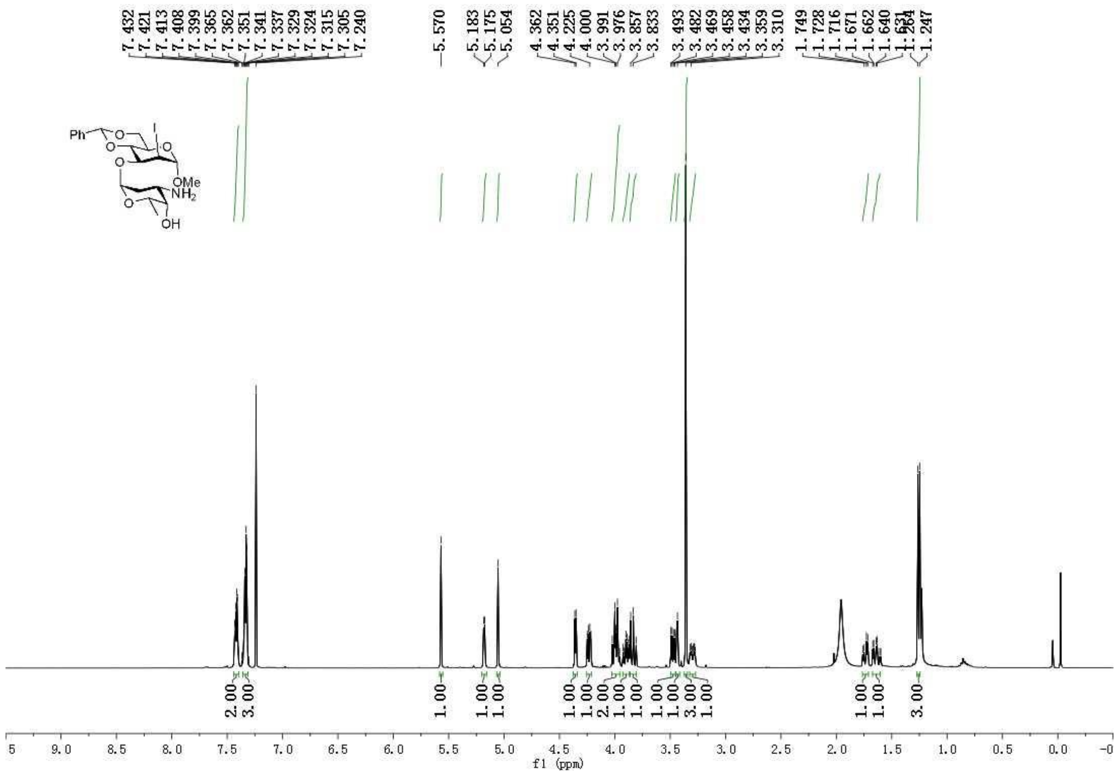

Figure S36. ^{1}H NMR spectrum of **2i** ($CDCl_3$, 400 MHz)

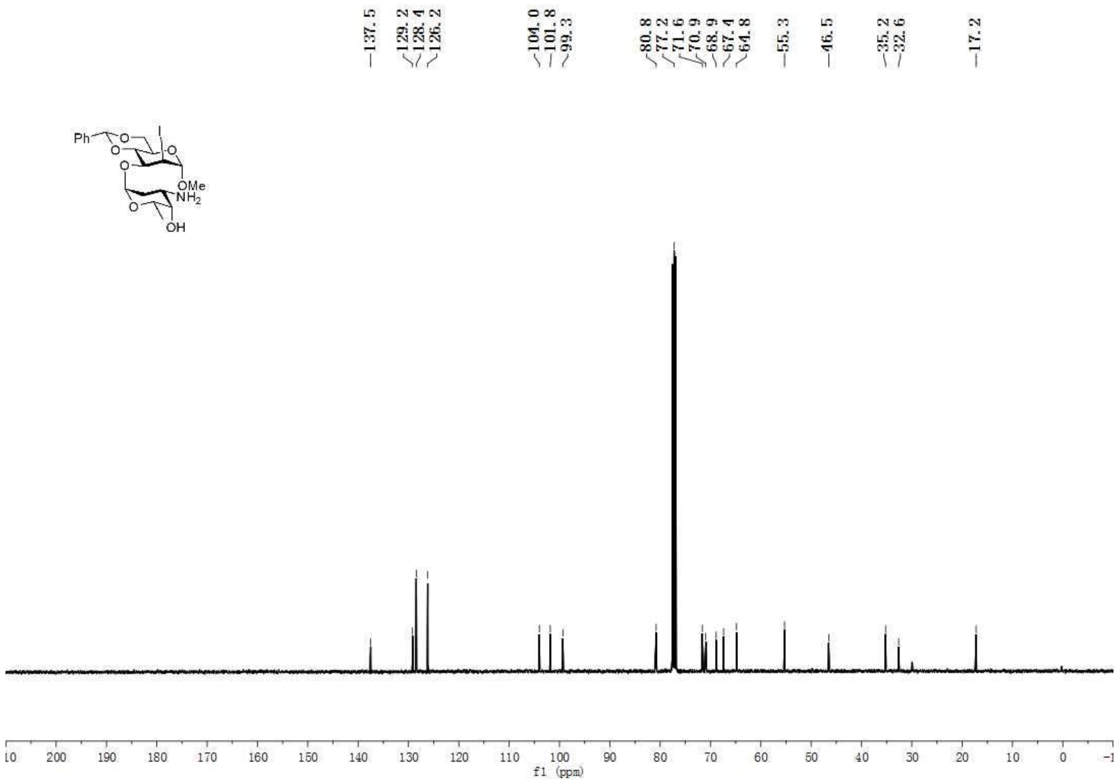

Figure S37. ^{13}C NMR spectrum of **2i** ($CDCl_3$, 100 MHz)

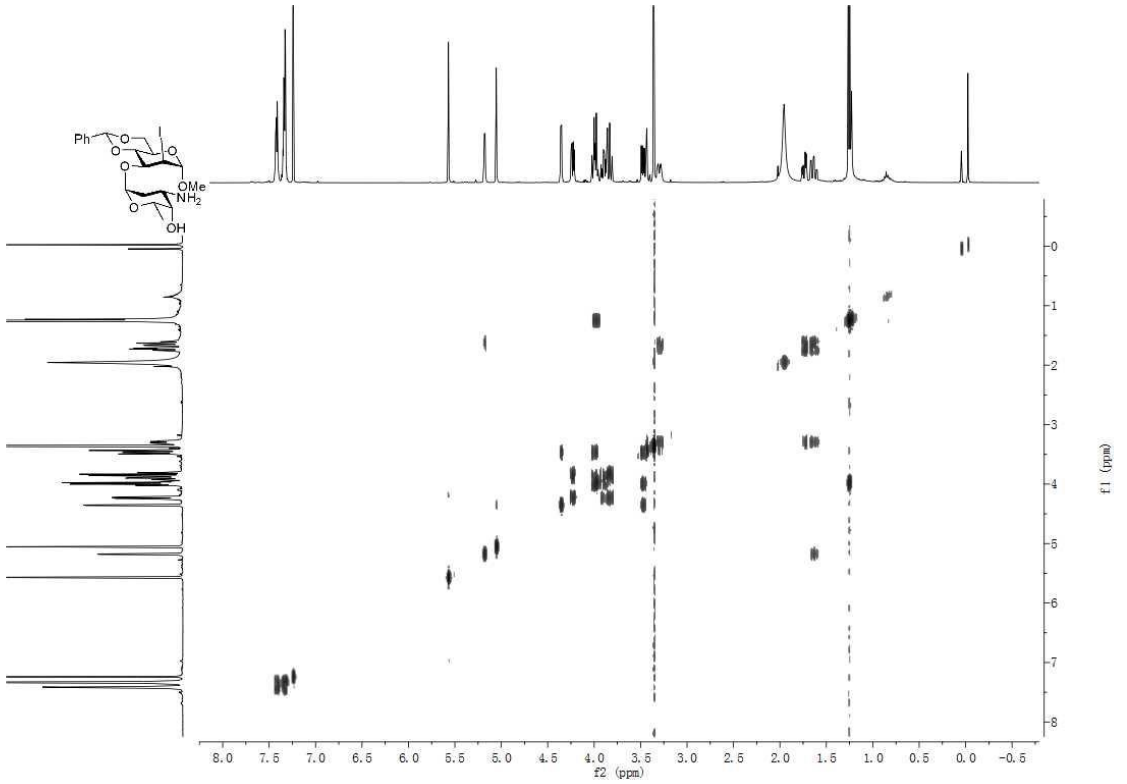

Figure S38. ^1H-^1H COSY spectrum of **2i** ($CDCl_3$, 400 MHz)

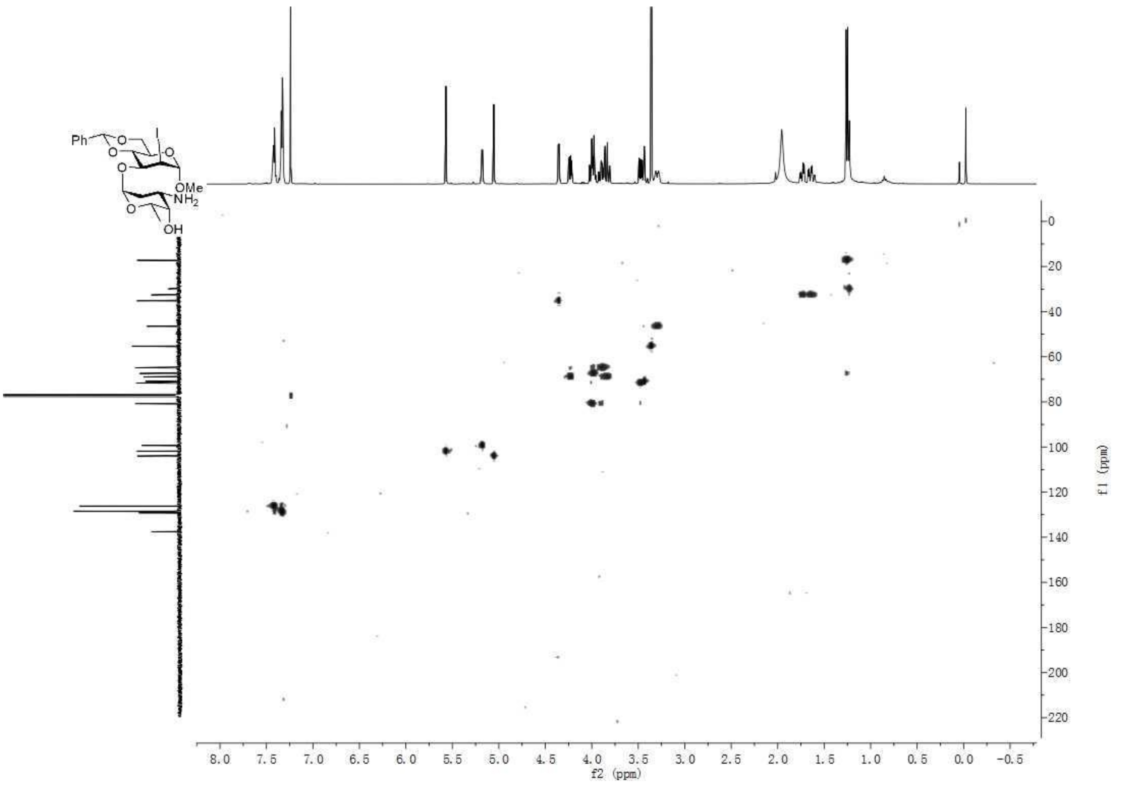

Figure S39. HSQC spectrum of **2i** ($CDCl_3$)

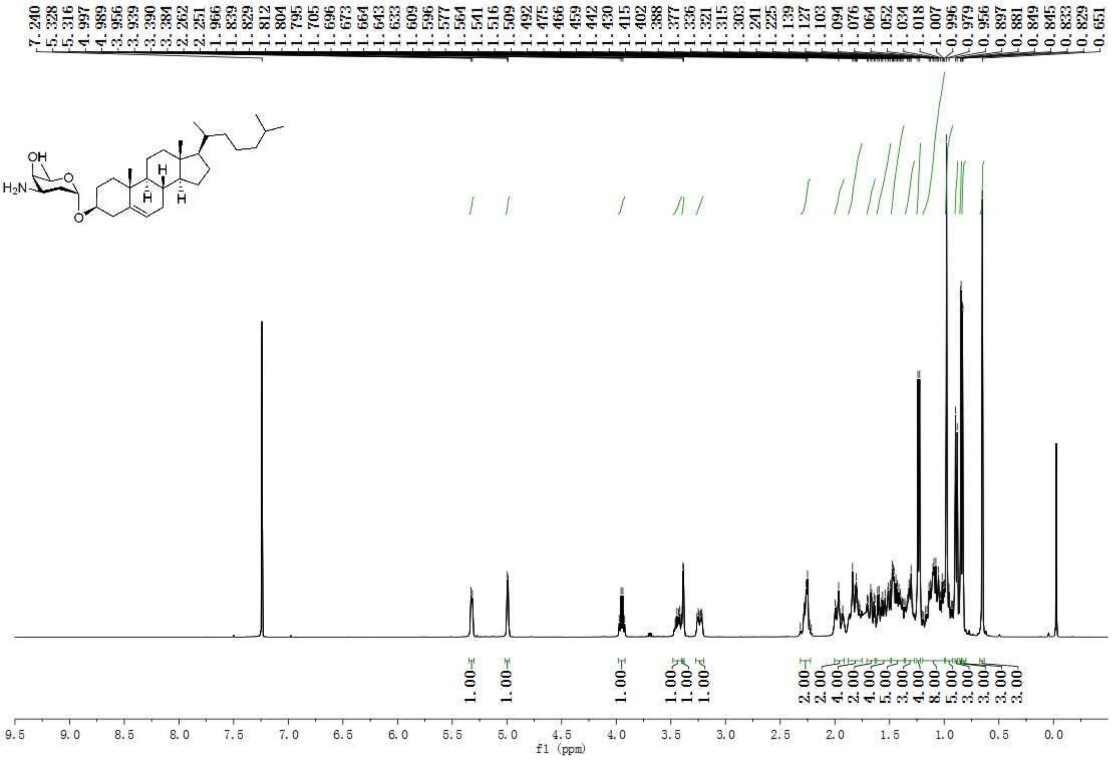

Figure S40. ^1H NMR spectrum of **2j** ($CDCl_3$, 400 MHz)

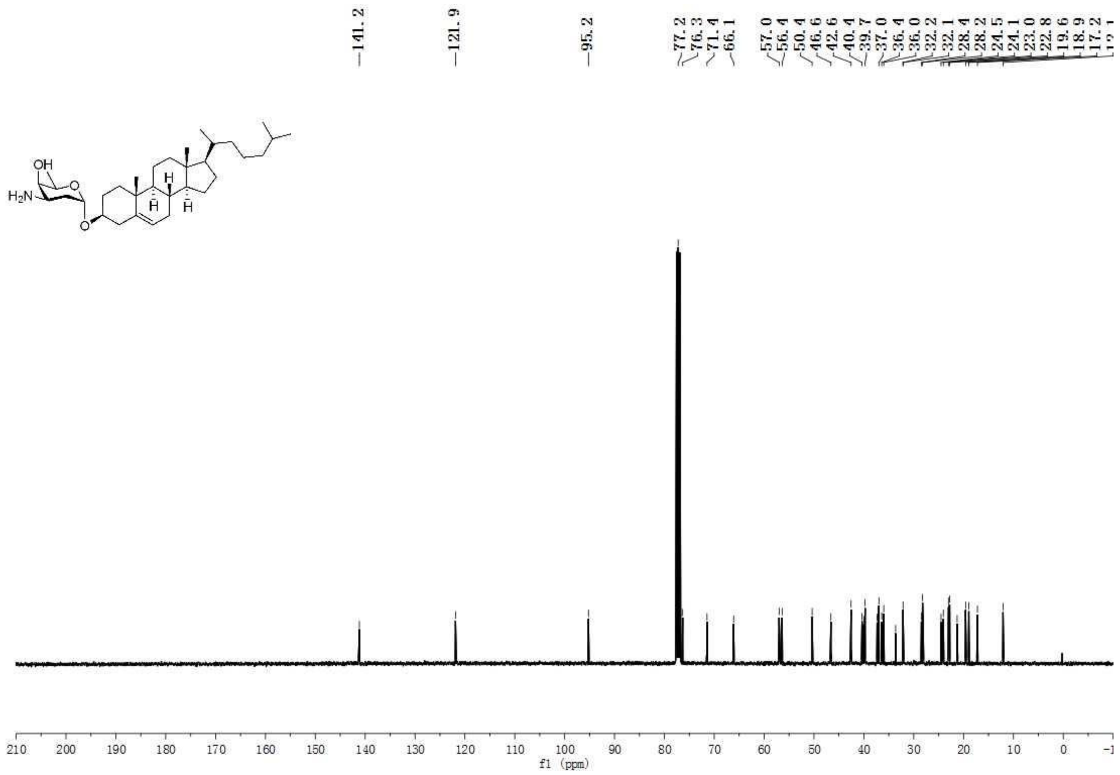

Figure S41. ^{13}C NMR spectrum of **2j** ($CDCl_3$, 100 MHz)

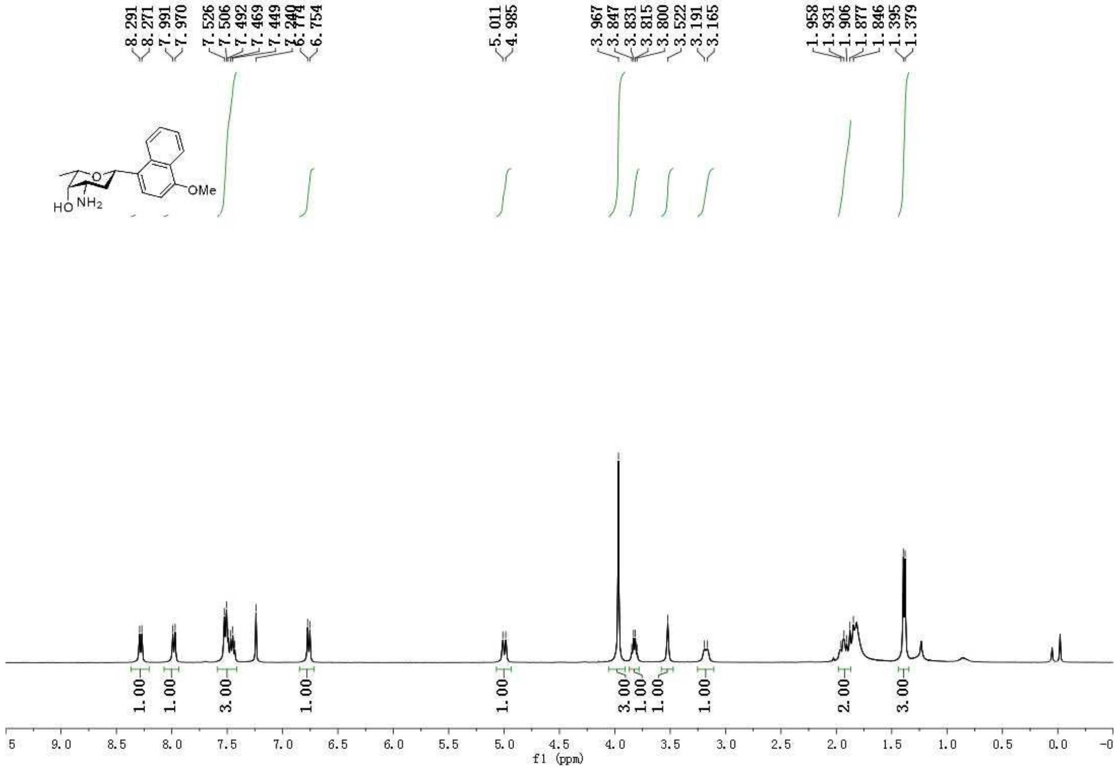

Figure S42. ^{1}H NMR spectrum of **2k** ($CDCl_3$, 400 MHz)

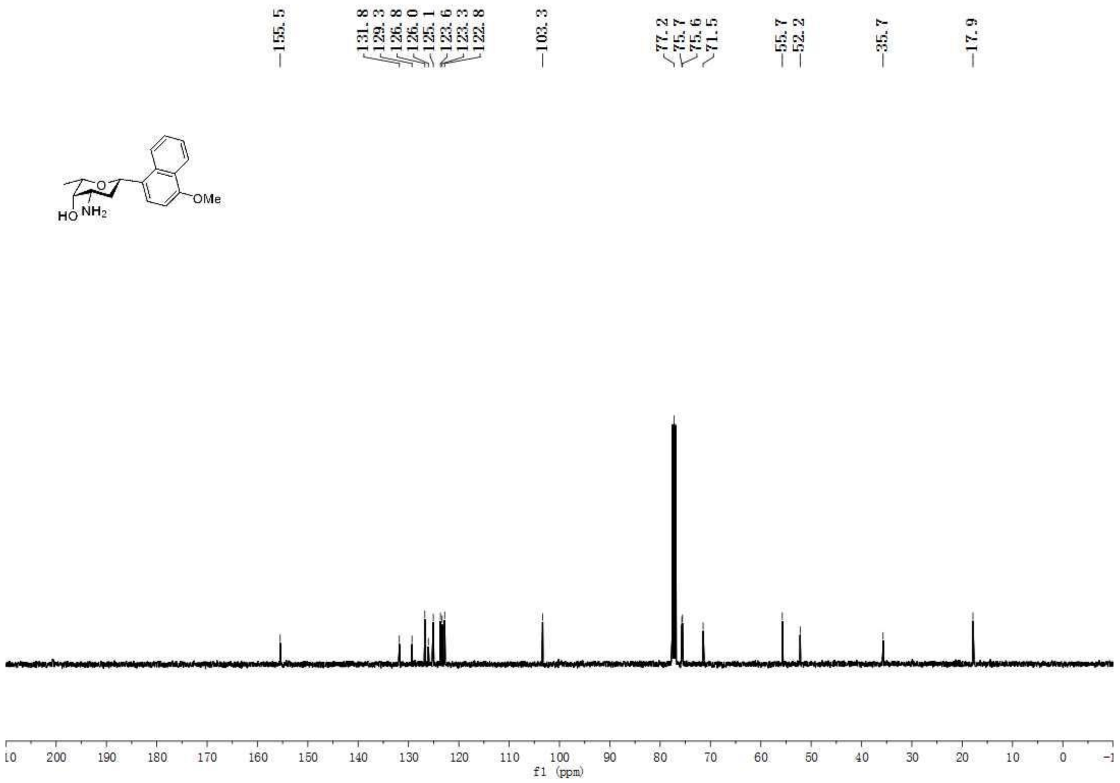

Figure S43. ^{13}C NMR spectrum of **2k** ($CDCl_3$, 100 MHz)

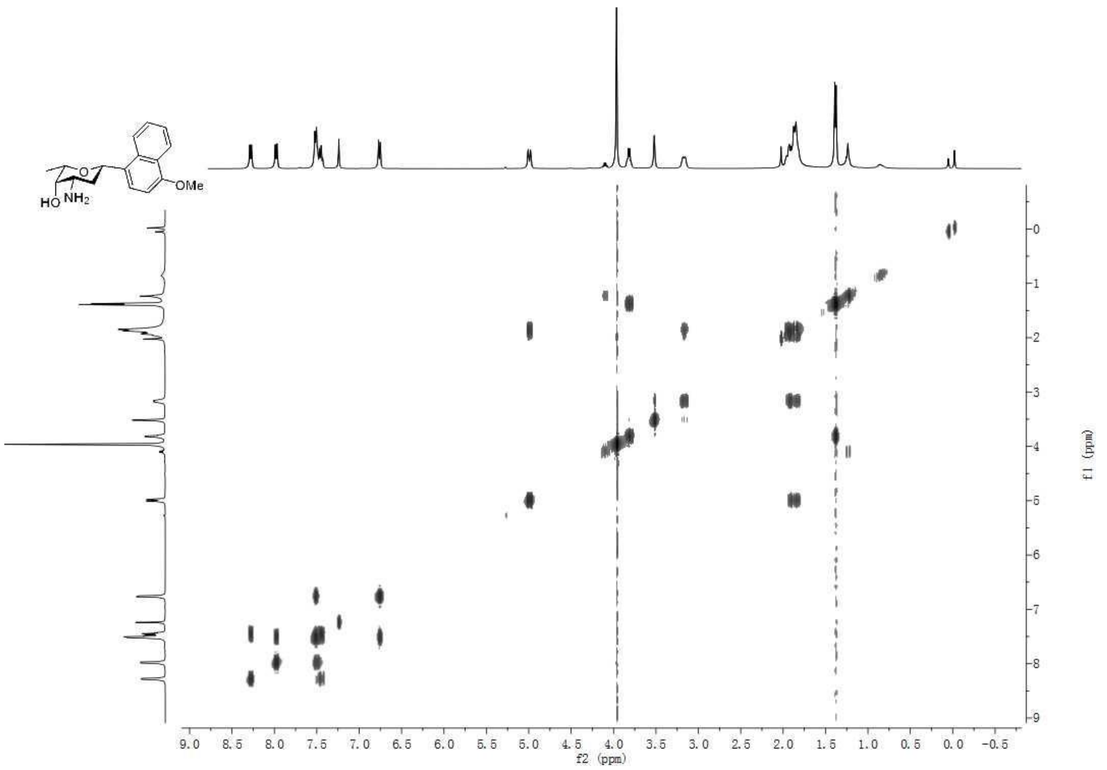

Figure S44. ^{1}H-^{1}H COSY spectrum of **2k** ($CDCl_3$, 400 MHz)

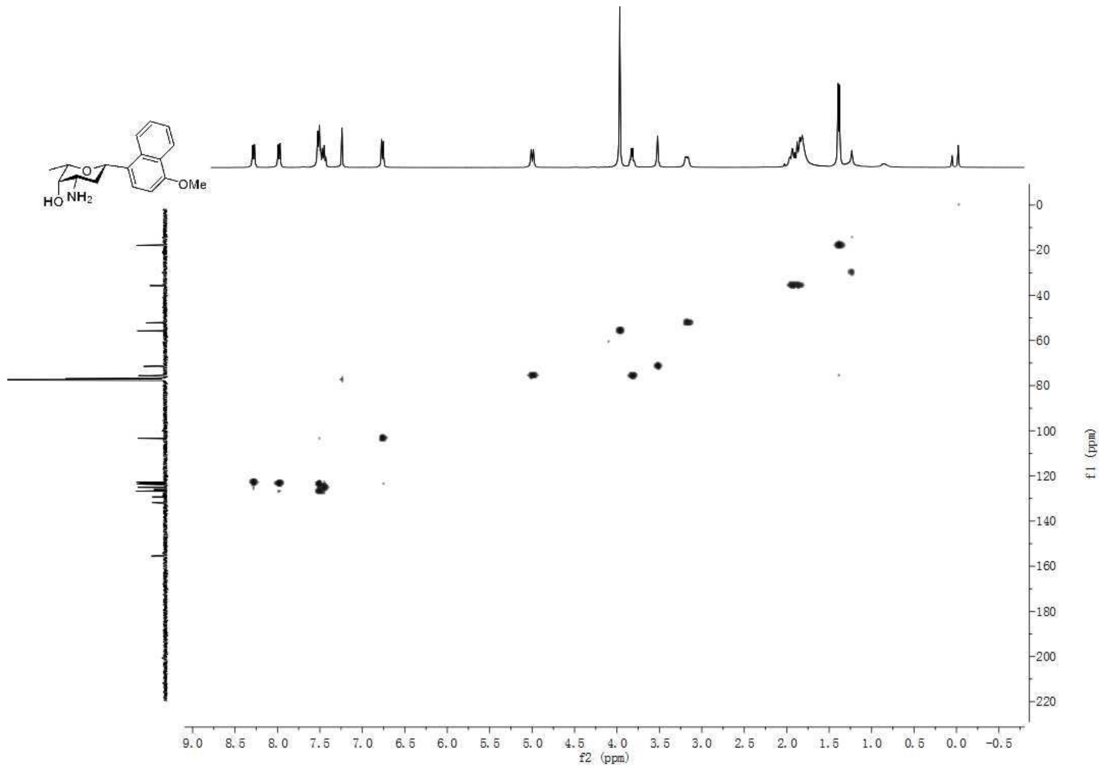

Figure S45. HSQC spectrum of **2k** ($CDCl_3$)

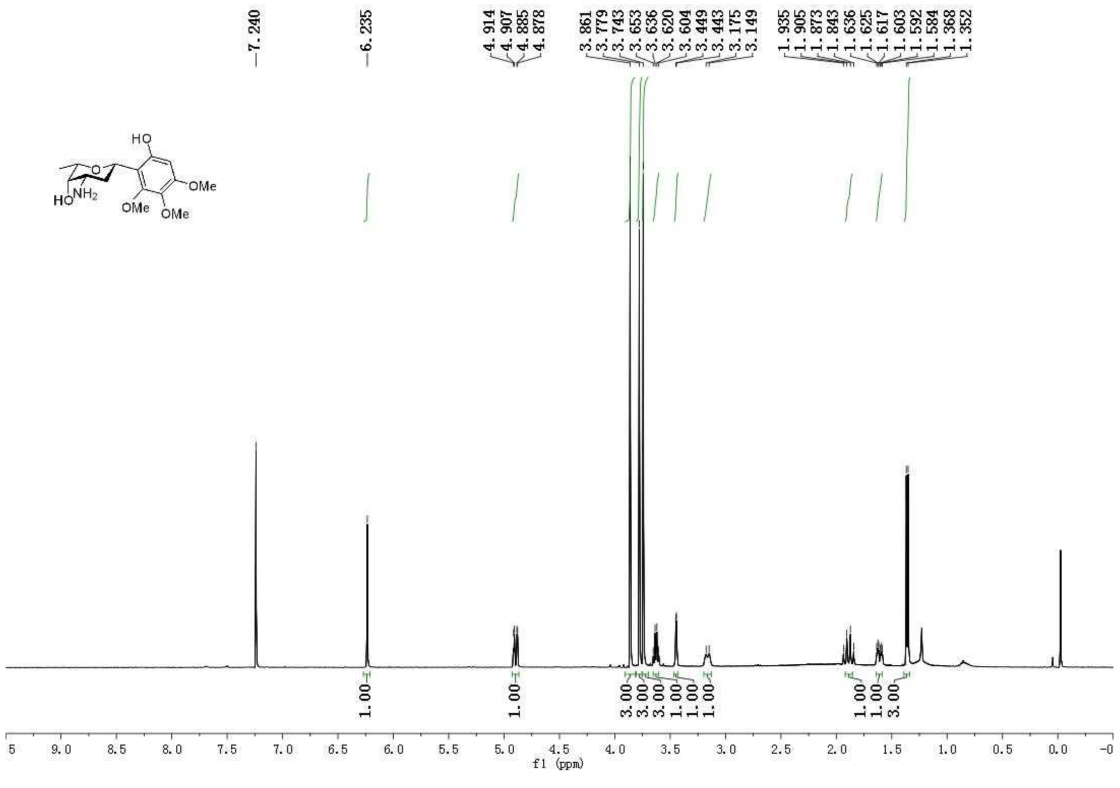

Figure S46. ^{1}H NMR spectrum of **2l** ($CDCl_3$, 400 MHz)

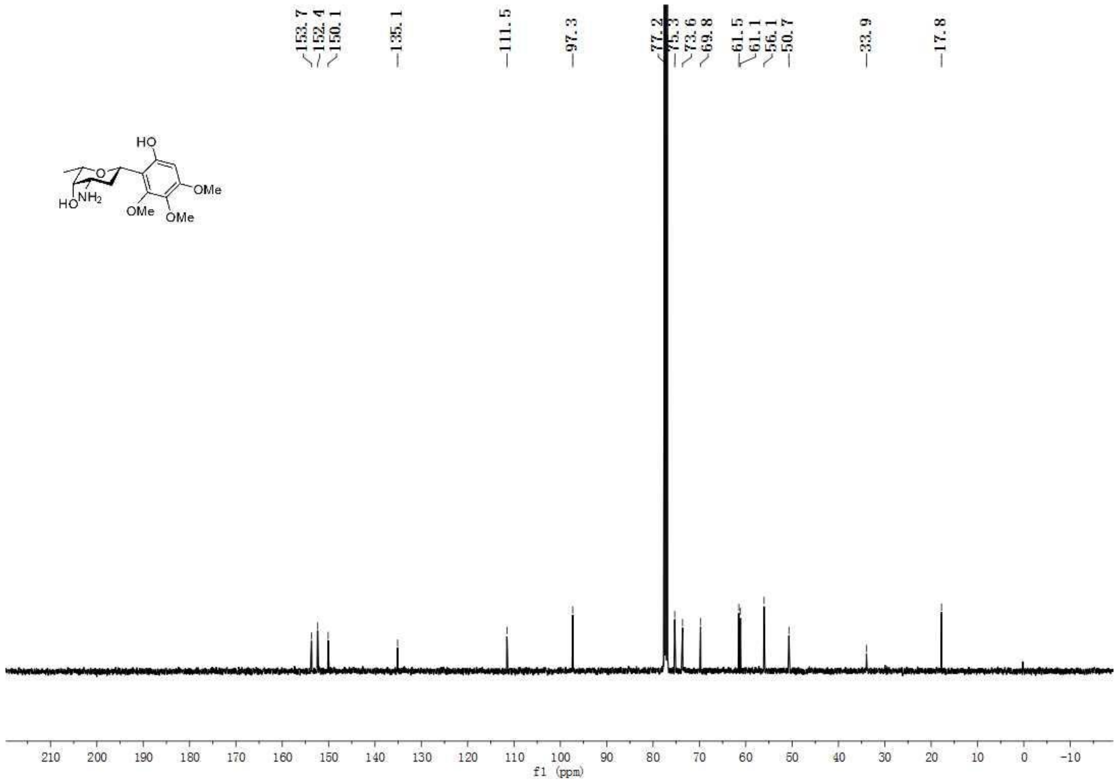

Figure S47. ^{13}C NMR spectrum of **2l** ($CDCl_3$, 100 MHz)

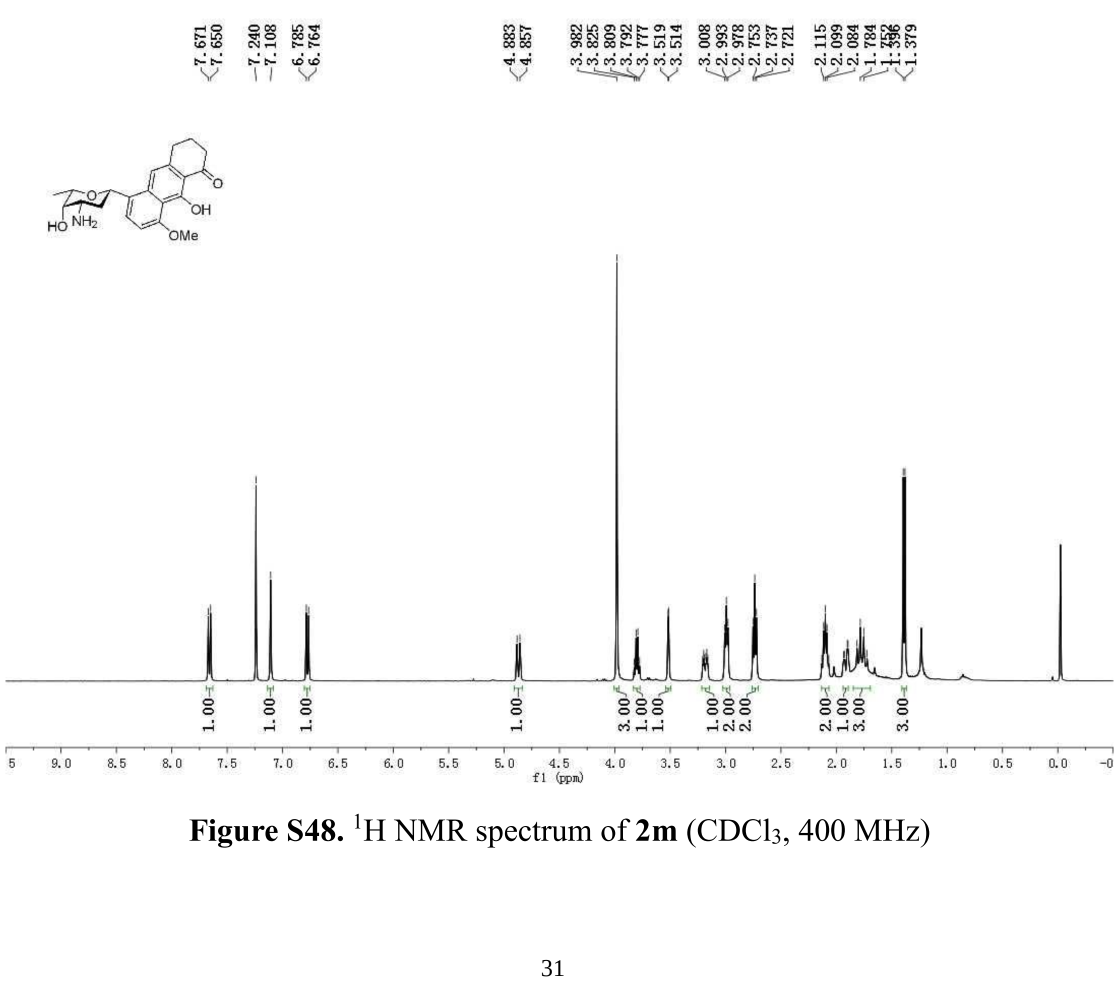

Figure S48. ^{1}H NMR spectrum of **2m** ($CDCl_3$, 400 MHz)

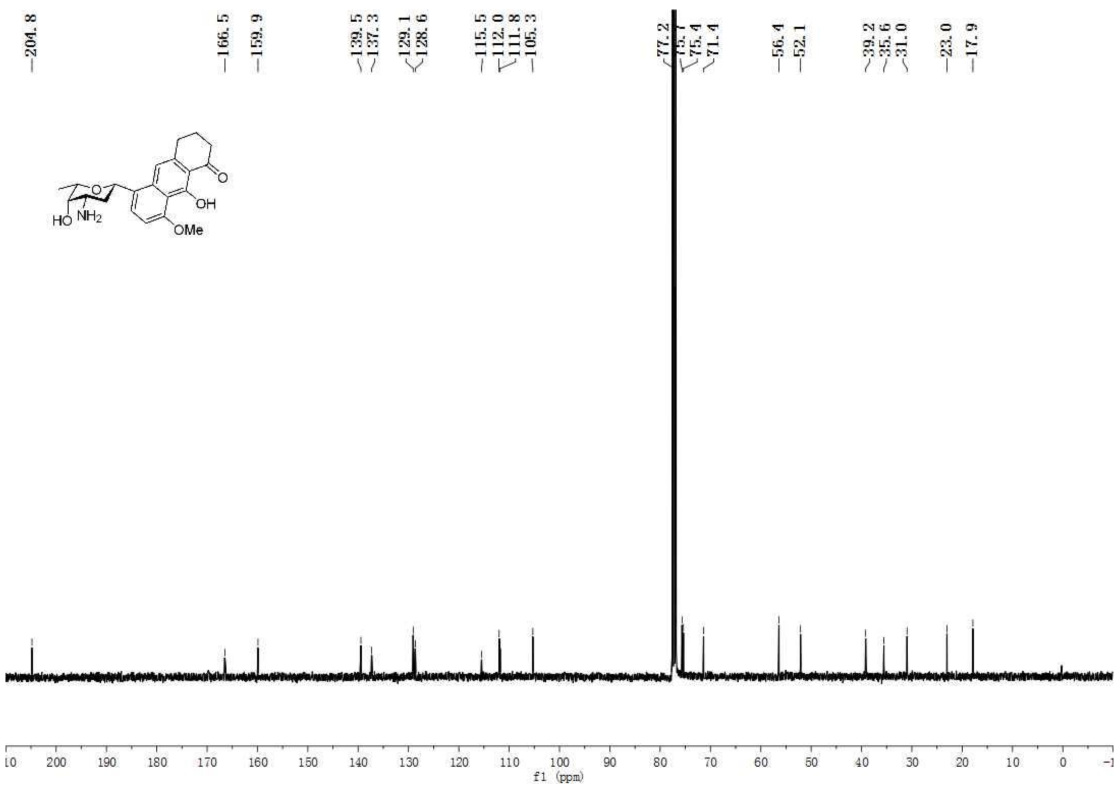

Figure S49. ^{13}C NMR spectrum of **2m** ($CDCl_3$, 100 MHz)

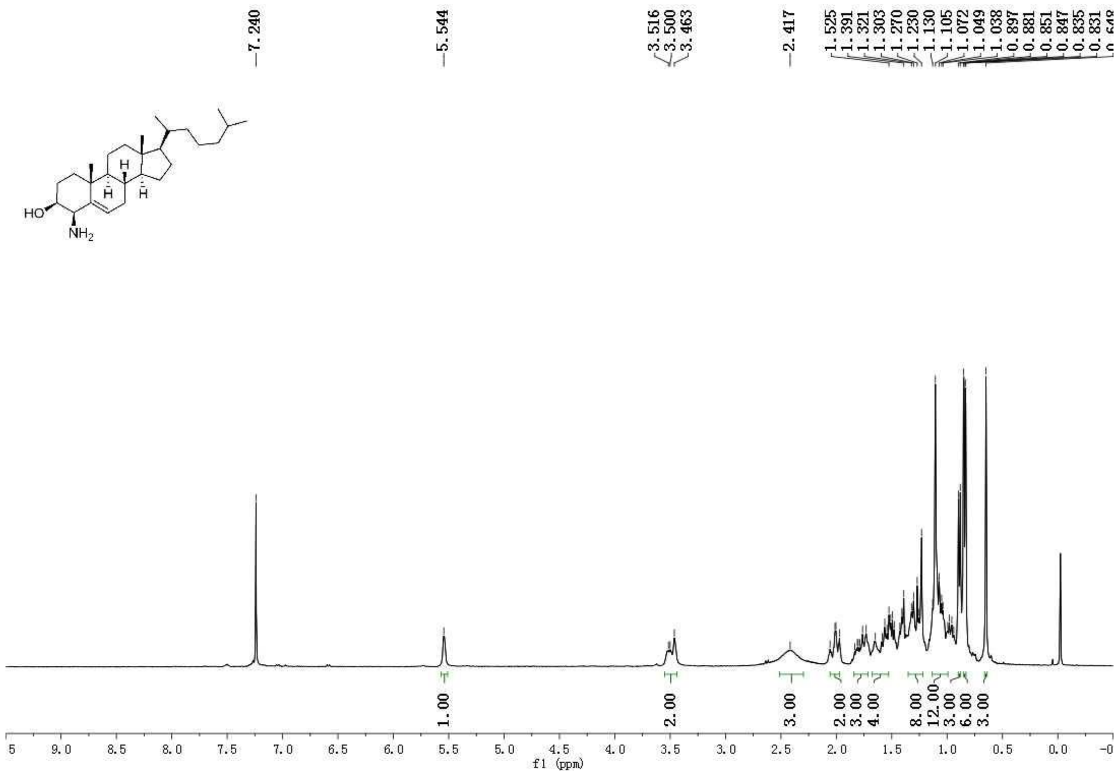

Figure S50. ^{1}H NMR spectrum of **7a** ($CDCl_3$, 400 MHz)

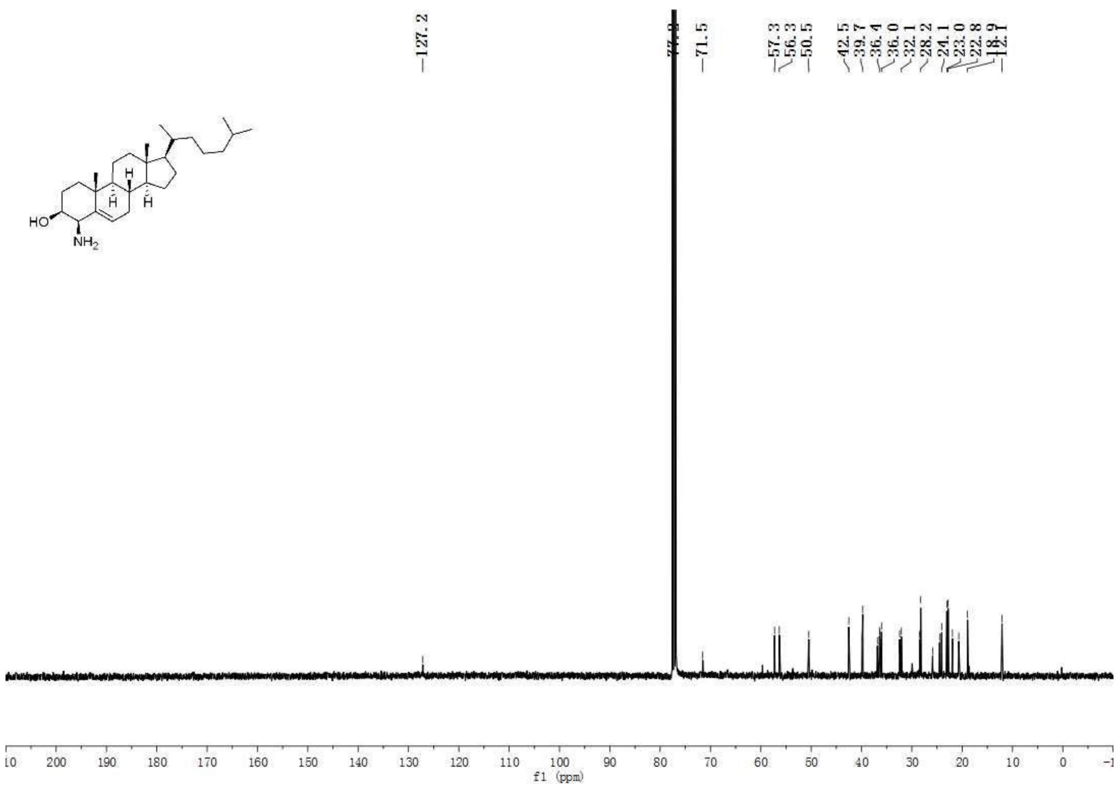

Figure S51. ^{13}C NMR spectrum of **7a** ($CDCl_3$, 100 MHz)

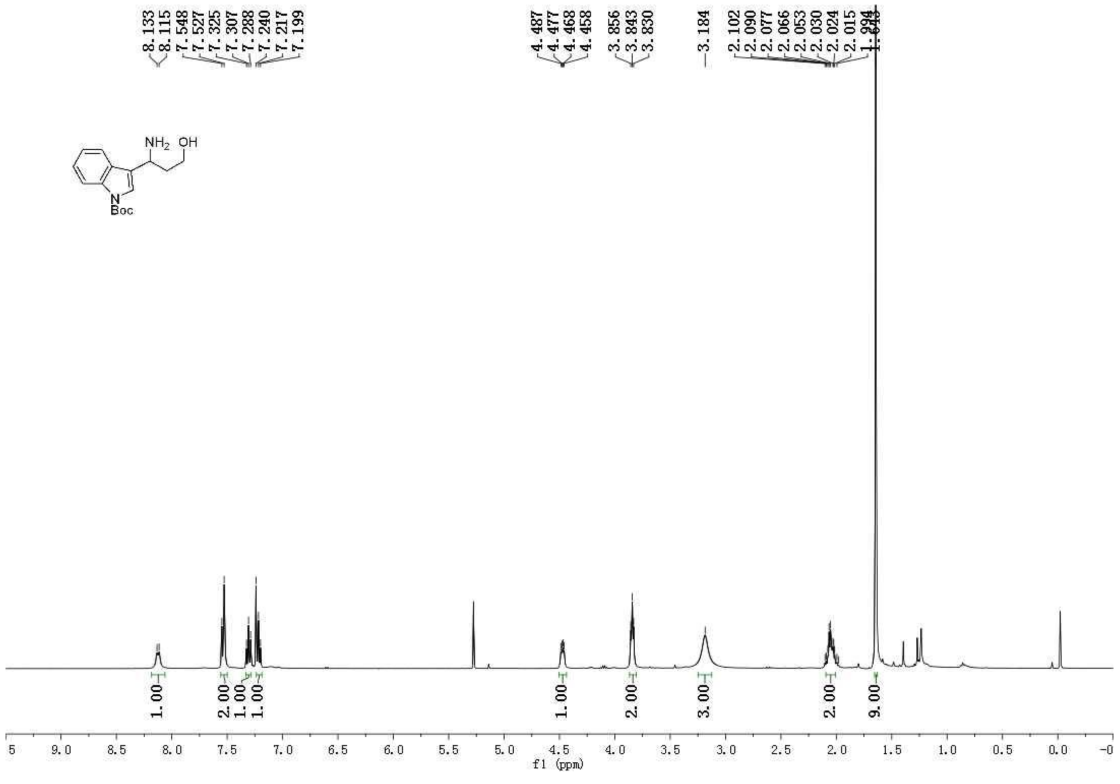

Figure S52. ^{1}H NMR spectrum of **7b** ($CDCl_3$, 400 MHz)

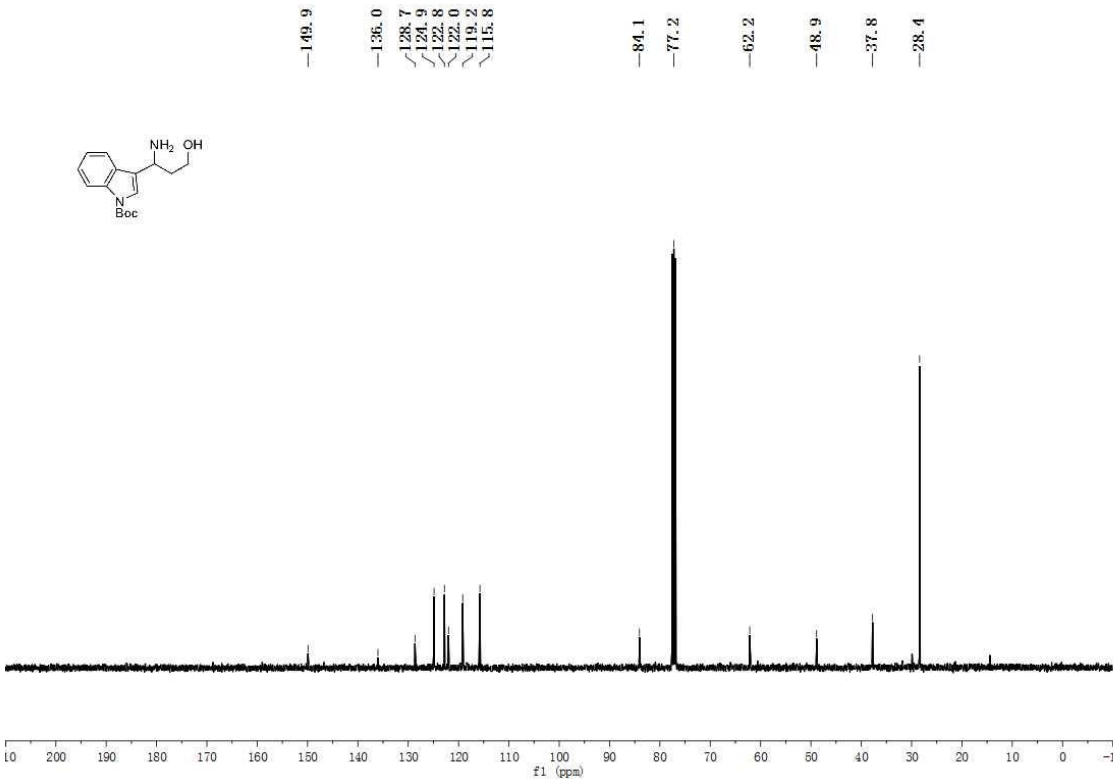

Figure S53. ^{13}C NMR spectrum of **7b** ($CDCl_3$, 100 MHz)
